# Regioselective Annulation of 6-Carboxy-Substituted Pyrones as a Two-Carbon Unit in Formal [4 + 2] Cycloaddition Reactions

**DOI:** 10.1021/acs.joc.4c01044

**Published:** 2024-06-13

**Authors:** Zachary A. Kohanov, Suzzudul Islam Shuvo, Andrew N. Lowell

**Affiliations:** † Department of Chemistry, Virginia Polytechnic Institute and State University (Virginia Tech), Blacksburg, Virginia 24061, United States; ‡ Center for Emerging, Zoonotic, and Arthropod-borne Pathogens, Virginia Polytechnic Institute and State University (Virginia Tech), Blacksburg, Virginia 24061, United States; § Faculty of Health Sciences, Virginia Polytechnic Institute and State University (Virginia Tech), Blacksburg, Virginia 24061, United States

## Abstract

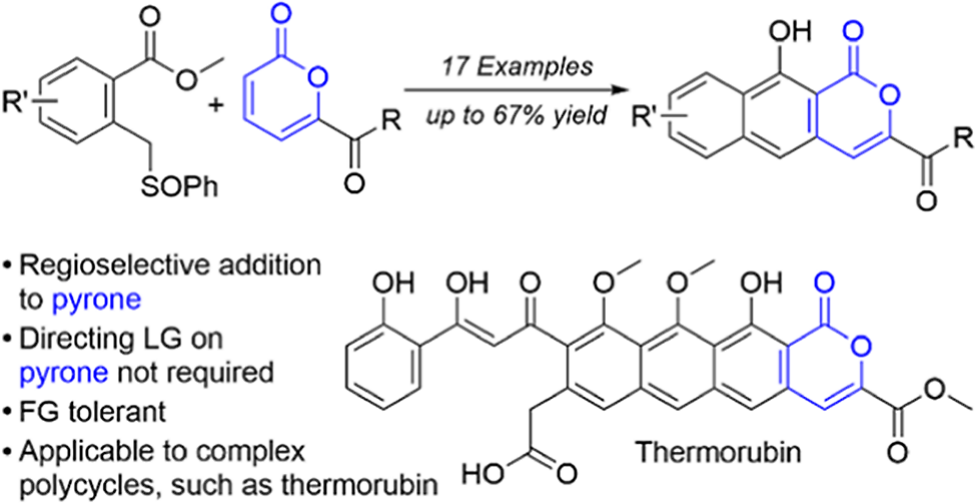

Heterocycles serve as a critical motif in chemistry, but despite being present in more than 85% of pharmaceuticals, there are limited methods for their construction. Here, we describe the incorporation of intact pyrone (2*H*-pyran-2-one) into larger ring systems via annulation. In a formal [4 + 2] cycloaddition, the pyrone regioselectively accepts a benzylic anion as a nucleophile in a conjugate addition fashion, with the subsequent pyrone-derived enolate attaching to a pendant ester on the initial nucleophile. Subsequent base-driven enolate formation and elimination establish aromaticity of the newly formed ring. After optimization of this process using an NMR-based assessment to overcome solubility and separation challenges, the reaction was successfully applied to a library of 6-ester and -amide-substituted pyrones and using a phenyl ester and other substituted sulfoxides. This technology enables the incorporation of intact pyrone rings into more complex systems, such as for the total synthesis of the natural product thermorubin.

## Introduction

Ring annulations, a critical reaction in synthetic chemistry, form heterocycles and polycycles that are integral to small molecule drugs^1^ and dyes.^2^ Despite their broad occurrence, forming multicyclic compounds remains challenging, especially in terms of achieving the desired substitution pattern on densely functionalized systems.^3,4^ Ring formation generally relies on modifying commercially available cyclic compounds. To form more complex polycycles, limited methods exist, often requiring costly or toxic catalysts,^4^ harsh conditions,^5^ or installing specific functionality to control regioselectivity in the presence of other competing functional groups.^6^

**Figure 1:**
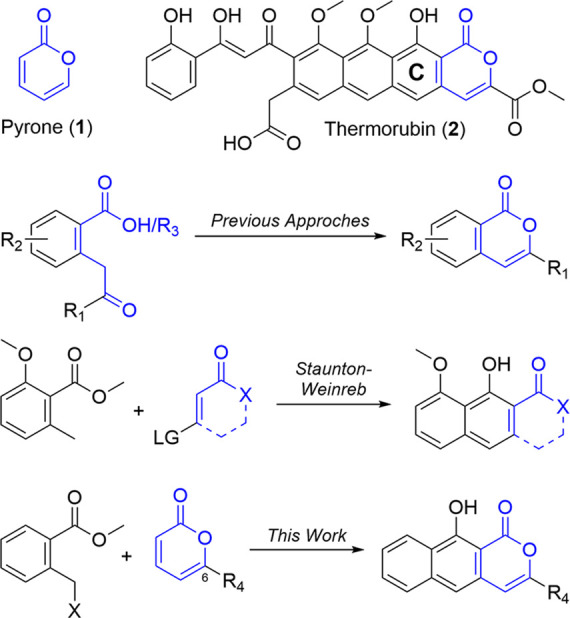
Pyrone-containing natural product thermorubin; previous and current approaches to annulated pyrones.

One particularly challenging ring is pyrone (2*H*-pyran-2-one, **1**, Figure 1), a heterocycle found in various pharmaceuticals and natural products.^7,8^ Often pyrone is conjugated to additional rings, such as in the natural product thermorubin (**2**).^9^ Many ring-forming reactions to establish annulated pyrones exist,^3,4,10^ all effectively proceeding through esterification to form the lactone. The enforced geometry and aromatic character of the pyrone make it fairly robust, thus its formation can occur early in a synthetic sequence.

Toward synthesizing aryl-pyrone-containing compounds, such as thermorubin, we envisioned directly annulating an intact pyrone to form a polycycle rather than the traditional approach of decorating an aromatic ring and subsequently cyclizing to generate the pyrone. Detractions of the traditional approach are the typical necessity of protecting groups for other substituents on the aromatic ring^11^ and harsh conditions^12^ or costly metal catalysts^13^ to facilitate closure to the pyrone. The Staunton–Weinreb approach enables annulation of *o*-methyl toluate with pyrones under basic conditions, but installation of a leaving group on the pyrone is required to enforce selectivity and establish aromaticity^14^ and a chelating group is required on the toluate for sufficient activation.^15^ Regioselective use of an unactivated pyrone as a two-carbon unit in what is formally a [4 + 2] cycloaddition is scarce, with only the 5-substituted pyrone enabling regioselectivity, but not chemoselectivity.^16^ Other substitution formats resulted in a blend of diene and dienophile roles for the pyrone and a lack of regioselectivity, particularly with 6-substituted pyrones.^17^ These findings made it unclear what conditions would enable the successful use of 6-substituted pyrones as dienophiles (their use as dienes is known)^18,19^ and what substitution pattern would result in the absence of a directing Staunton–Weinreb-type leaving group. Thus, we investigate the parameters that would enable the regioselective use of pyrones as dienophiles toward the synthesis of thermorubin.

## Results and Discussion

**Scheme 1:**
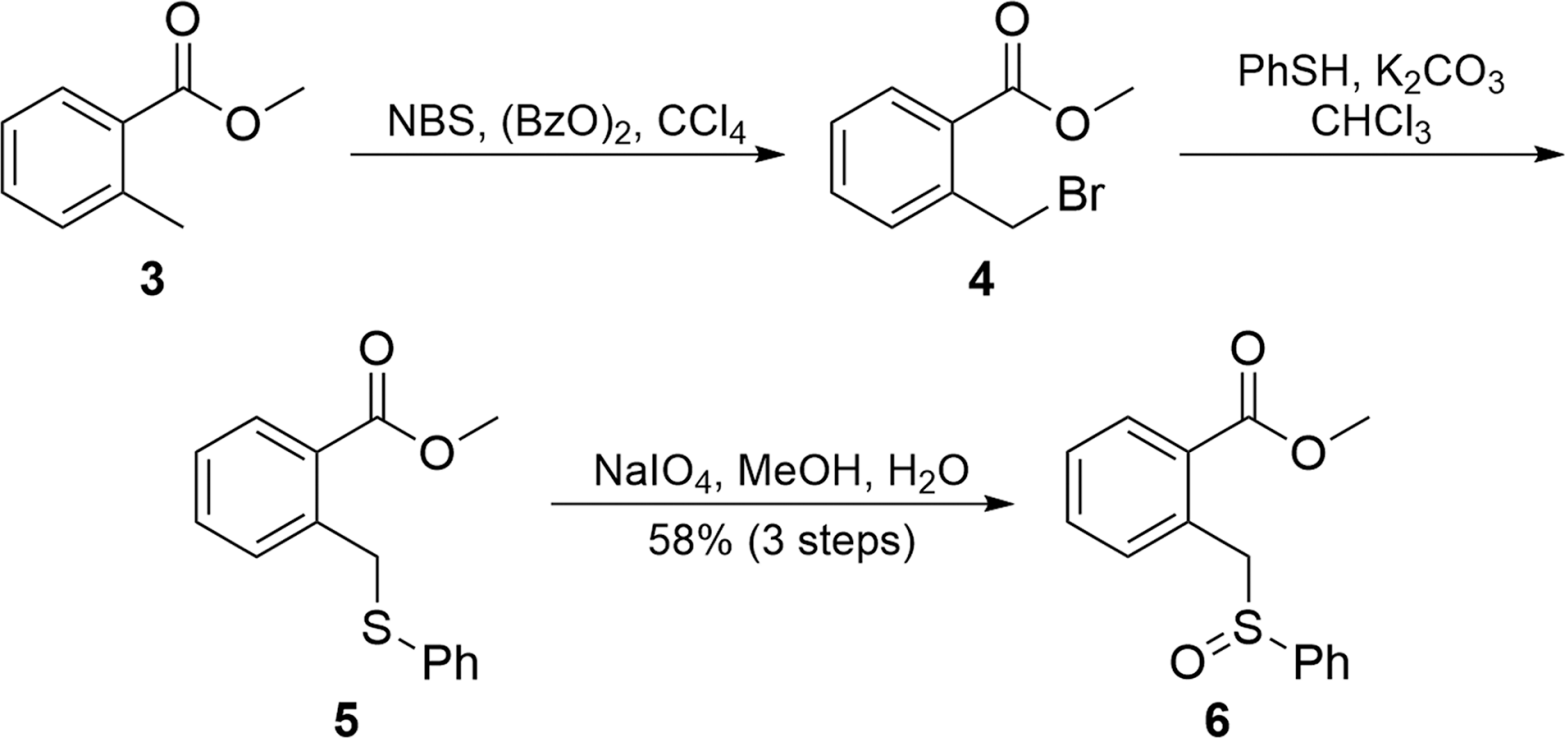
Synthesis of Diene Surrogate **6**

For the diene portion, several well-established synthons^20^ are available that would enable rearomatization after reaction with pyrones, forming either a single phenol *peri* to the pyrone carbonyl or a *p*-hydroquinone. To determine the suitability of pyrones as dienophiles, we selected methylbenzoate sulfoxide **6**(21) (Scheme 1) as a representative test partner that we could prepare from **3** via a bromination (**4**), thioetherification (**5**), and oxidation (**6**) sequence^22^ in good yield. After annulation, a diene such as **6** would provide the functionality pattern present in the thermorubin C-ring.

**Scheme 2:**
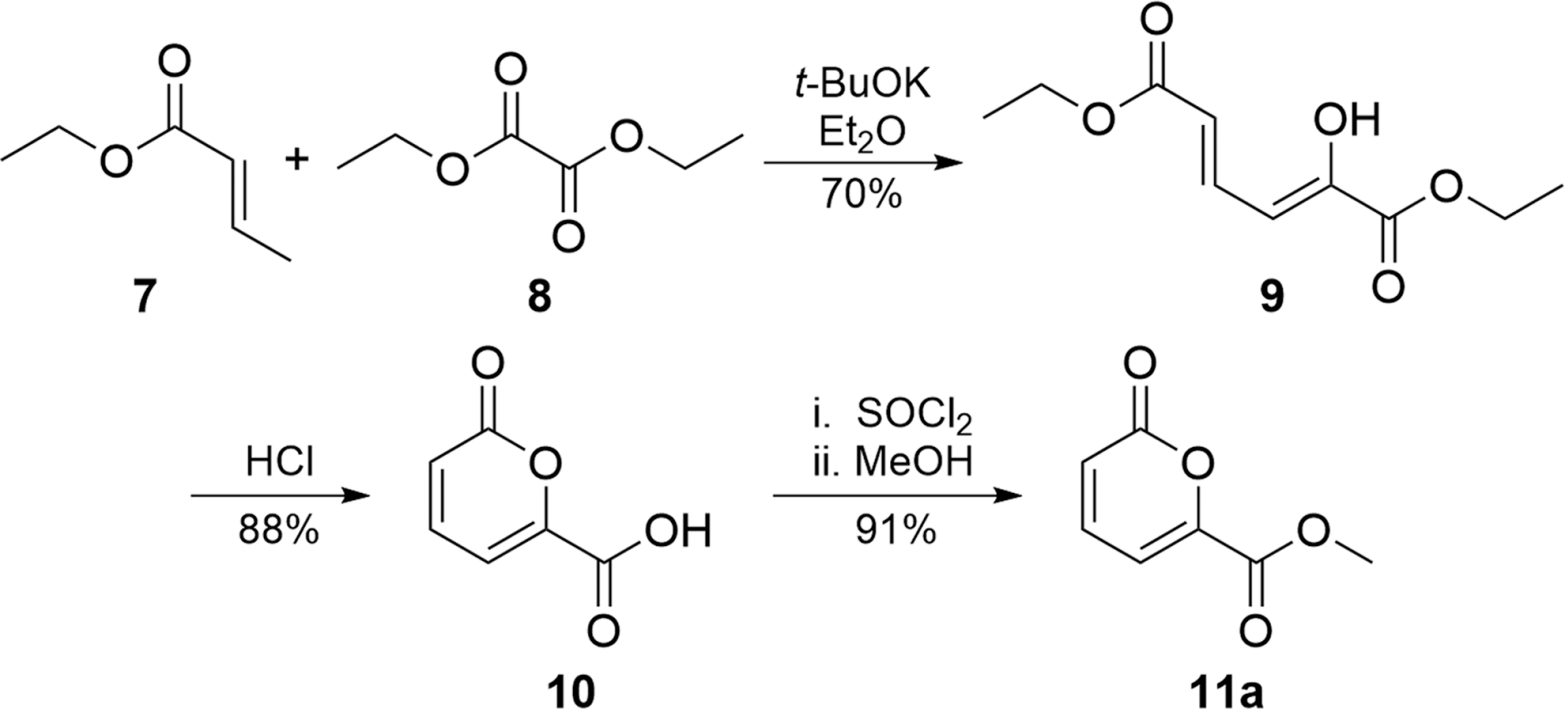
Preparation of Dienophile Methyl 6-Carboxylate Pyrone (**11a**)

With a suitable diene-equivalent in hand, the questions left to resolve were ones of activity and regiochemistry. We hypothesized that the pyrone was sufficiently active to react with a diene in either a stepwise or concerted fashion and that the diene would add to the α–β position relative to the pyrone carbonyl. We questioned this hypothesis because for pyrones such as **11a** (Scheme 2), which mimics the D-ring of thermorubin, the δ position is also conjugated to the lactone and even the γ position is activated because of conjugation to the *exo*-ester. However, the enforced *syn* orientation of the pyrone ester makes them strong electron-withdrawing groups, more so than the *exo*-ester. Coupled with cross-conjugation from the lactone oxygen, we expected the lactone to dominate, suppressing the electrophilicity of the γ position. Furthermore, if the initial attachment of the diene nucleophile has any reversibility, addition to the more sterically hindered δ position—which cannot reattain aromaticity because the intermediate is a quaternary center—would be reversible.

Our initial trials used sulfoxide **6** with methyl ester pyrone **11a** (Scheme 2), as their annulation product would be analogous to the BCD ring of thermorubin. Pyrone **11a**(23,24) was synthesized through the reaction of ethyl crotonate (**7**) with diethyl oxalate (**8**) under basic conditions to give dienedioate **9**, which after treatment with concentrated hydrochloride acid, cyclized into pyrone carboxylic acid **10**. Acid **10** was converted into pyrone methyl ester **11a**(23) by first refluxing **10** in thionyl chloride and, after removal of excess thionyl chloride through distillation, treatment of the resulting acid chloride with methanol. An initial trial reacting **6** with **11a** using lithium diisopropylamide (LDA) in tetrahydrofuran (THF) validated our hypothesis; α–β addition and rearomatization was observed as the exclusive product, albeit in a modest yield, with sulfoxide and pyrone also recovered.

To optimize the production of annulated product **12a** (Table 1), we began screening conditions based on prior work using sulfoxide **6** with α–β unsaturated esters^6,21,25^ and ketones.^22,26,27^ Initial trials varying the equivalencies of the LDA base (entries 1–5) showed a decrease in the production of **12a** when fewer equivalencies of the base were used (entries 1 and 2) and a modest increase with 3.0 equiv (entry 3). Further increases in the amount of base abolished the production of **12a** (entries 4 and 5). Reducing the equivalents of pyrone also decreased the yields (entries 6 and 7) while excess (entry 8) or proportionate (entry 9) increases of pyrone relative to the base increased the yield. A large increase (entry 10) decreased the yield, likely due to interference by excess base as per entries 4 and 5. Altering the addition order (entries 11–14) decreased the yield or resulted in no product when the pyrone was mixed with LDA first. Changing the addition temperature and quenching protocol slightly decreased the yield (entry 15). The screening of different bases showed that alkoxides and hydrides were unsuitable (entries 16–18) while LiHMDS (entry 19) was essentially as effective as LDA as a base.

**Table 1:**
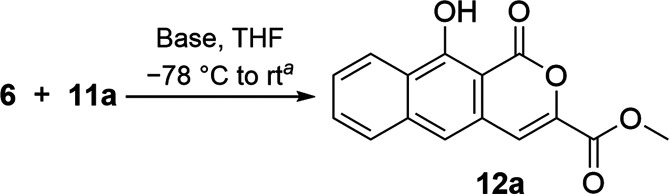
Annulation Trials between **6** and **11a** Assessed by Isolation^a^

entry	base	base equiv	pyrone equiv	% yield
1	LDA	1.1	2.4	28
2	LDA	1.65	2.4	28
3	LDA	3.0	2.4	43
4	LDA	4.4	2.4	<1
5	LDA	8.8	2.4	<1
6	LDA	2.2	1.2	<9
7	LDA	2.2	1.9	<9
8	LDA	2.2	4.8	48
9	LDA	4.4	4.8	47
10	LDA	6.6	7.2	22
11^b^	LDA	2.2	2.4	19
12^c^	LDA	2.2	2.4	17
13^d^	LDA	2.2	2.4	13
14^e^	LDA	2.2	2.4	0
15^f^	LDA	2.2	2.4	26
16	*t*-BuOLi	2.2	2.4	3
17	*t*-BuOK	2.2	2.4	3
18	NaH	2.2	2.4	0
19	LiHMDS	2.2	2.4	27

a Sulfoxide **6** added to the base followed by pyrone **11a** unless otherwise noted.

b Addition of base to **6**.

c Addition of base to **6** and **11a**.

d A premixture of **6** and base were added to **11a**.

e Pyrone **11a** added to the base followed by **6**.

f Base/**6** mixture warmed to −40 °C prior to the addition of **11a**, then quenched at −40 °C with NH_4_Cl.

**Figure 2:**
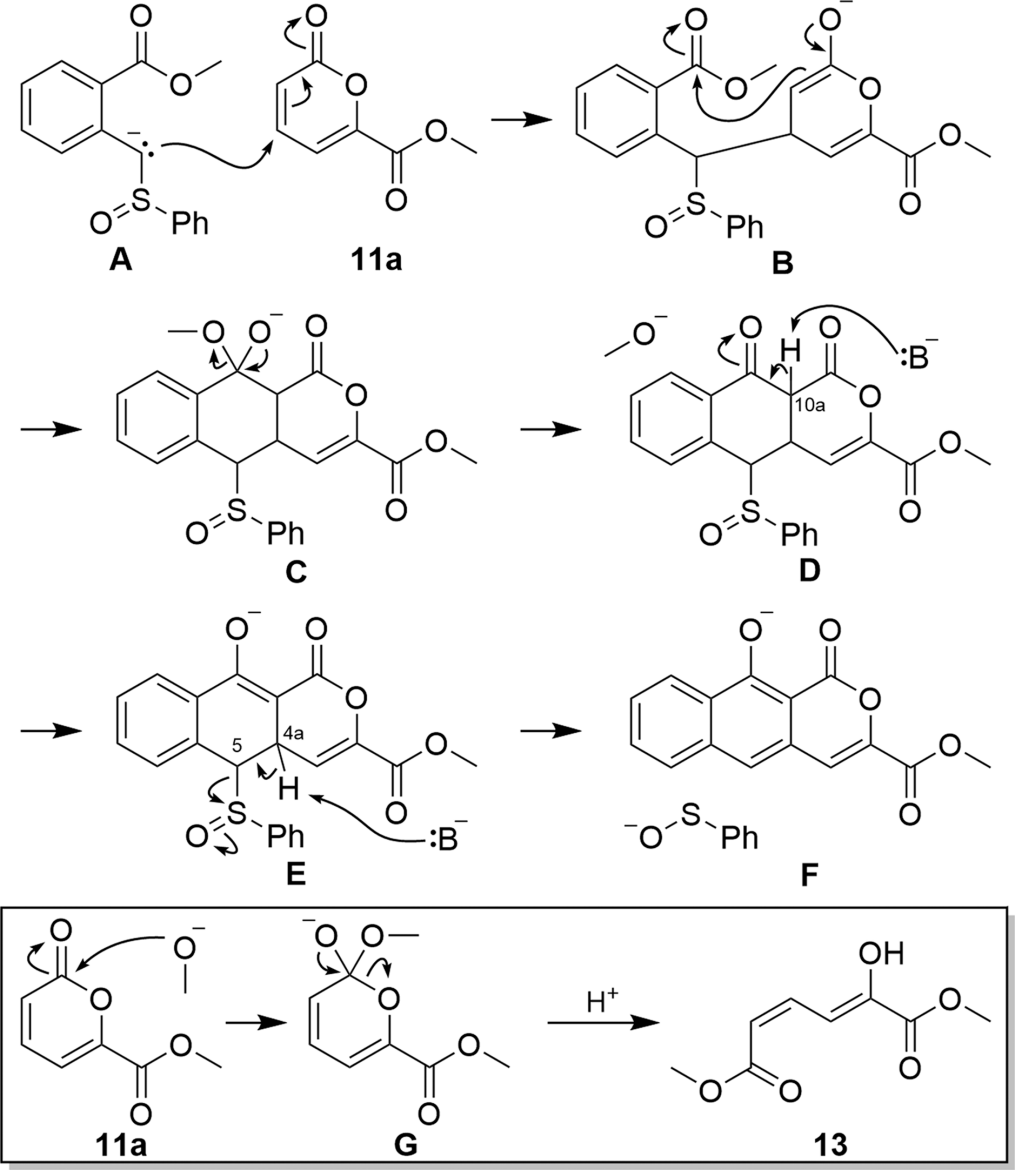
Proposed mechanism of pyrone annulation. *Inset*: Reaction of methoxide with pyrone **11a** to give **13**.

While this process enabled initial optimization of this reaction, batch-to-batch variation made comparisons difficult, necessitating a concurrent control reaction. Furthermore, the separation of the product from other materials proved challenging, significantly slowing progress. Besides the desired product **12a**, we were able to isolate recovered sulfoxide **6** and pyrone **11a**, along with a byproduct that showed features similar to pyrone **11a**. Characterization of this material revealed it to be ring-opened **11a**, where methoxide generated during the course of the reaction acted on the pyrone carbonyl to form **13** (Figure 2, *inset*).

This finding spurred careful analysis of the proposed mechanism (Figure 2) to build on the isolated yield data and identify potential pitfalls. Three equivalents of base are needed, one to generate the initial sulfoxide anion (**A**), a second to deprotonate C10a of **D**, and a third to establish aromaticity by deprotonating C4a and eliminating sulfenate from **E**. While the methoxide generated when **C** converts into **D** would be sufficient to deprotonate C10a, the resulting methanol would be deprotonated by remaining stronger base, and it was unclear if methoxide would be sufficient to cause elimination (**E** to **F**). The methoxide also reacts with pyrone **11a** (*inset*), requiring extra equivalents of this starting material, and the conjugate base of **13** is certainly not sufficient for elimination. While we were worried that competitive deprotonation of the C5 position in the presence of a strong base would interfere with the desired annulation reaction, we were never able to isolate unaromatized byproducts and had a good mass balance of the desired product **12a** and recovered sulfoxide **6**. Three equivalents of base are ideal and necessary, but we found that LDA would also consume pyrone **11a**, which prevented high yields through the unwanted reaction of LDA with pyrone.

To continue optimizing the reaction while avoiding purification difficulties, we employed an NMR-based assessment method in order to quantify yields more rapidly than we could with isolation. By running the reaction through workup, collecting the mass of the dry, impure material, and then adding a known quantity of *o*-xylene as an internal standard to a portion of the product mixture, we could reliably quantify the absolute ratio of each component and easily ascertain how varying conditions affected the yield. Using this approach, we continued experimentation (Table 2) with different bases, additives, and equivalents. The use of precedented^21^ 2.2 equiv of LDA as a base gave a modest yield (entry 20) and did not show significant change when dimethyl sulfoxide (DMSO) was added as a cosolvent (entry 21). In contrast to the isolated results showing LiHMDS being similar to LDA, its use in this case showed a slight improvement in yield (entry 22). Increasing the equivalents of base increased the yield (entry 23), but the addition of lithium (entry 24) or its sequestration with 12-crown-4 (entry 25) did not increase yields. Experimentation with other bases showed that LiTMP reduced the yield (entry 26) while use of LiHMDS at three equivalents again improved outcomes, surpassing a 50% yield for the first time (entry 27). Use of other alkali metals as the counter cation with the HMDS anion gave the product (entries 28 and 29), but in lower yields. Mirroring the isolation experiments, the use of excess LDA and **11a** decreased yields (entries 30 and 31), although this decrease could be rescued somewhat by the addition of tetramethylethylenediamine (TMEDA, entry 32). Incubation of LDA and LiHMDS with pyrone **11a** in the absence of sulfoxide **6** revealed that while LDA degraded **11a**, LiHMDS did not. Thus, we shifted our focus to LiHMDS. Addition of TMEDA with LiHMDS did not increase yields (entry 33), nor did increased equivalents of LiHMDS (entry 34), though the product was observed in this case, unlike with LDA. Premixing LiHMDS with pyrone **11a** followed by sulfoxide (**6**) addition gave reasonable product formation (entry 35) and increasing the concentration by reducing the amount of solvent used to dissolve sulfoxide **6** and prepare the base (entry 36) gave a good yield. We believe this highest yield results from a combination of less water contamination during the dissolution of sulfoxide **6** and more available base to quickly push the intermediates, especially **E** (Figure 2) through the elimination. With optimized conditions in hand, we proceeded to isolate the products of the reaction and were pleased to find we could synthesize **12a** in a 53% isolated yield. Based on this success, we sought to explore the generality of this reaction using **6** and other pyrone derivatives.

**Table 2:**
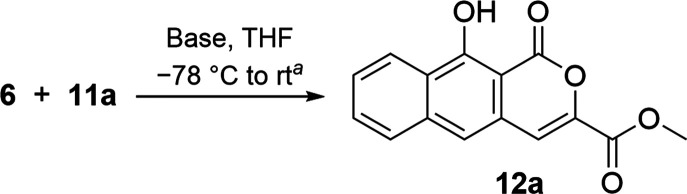
Annulation Trials between **6** and **11a** Assessed by NMR^a^

entry	base	base equiv	pyrone equiv	cosolvent/additive	% yield
20	LDA	2.2	2.4	–	27
21	LDA	2.2	2.4	DMSO^b^	30
22	LiHMDS	2.2	2.4	–	37
23	LDA	3.0	2.4	–	48
24	LDA	3.0	2.4	LiCl^c^	34
25	LDA	3.0	2.4	12-crown-4^d^	34
26	LiTMP	3.0	2.4	–	24
27	LiHMDS	3.0	2.4	–	57
28	NaHMDS	3.0	2.4	–	21
29	KHMDS	3.0	2.4	–	19
30	LDA	4.4	4.8	–	34
31	LDA	6.6	7.2	–	1
32	LDA	4.4	4.8	TMEDA^e^	45
33	LiHMDS	3.0	2.4	TMEDA^e^	44
34	LiHMDS	8.0	2.4	–	33
35^f^	LiHMDS	3.0	2.4	–	48
36^f^^,^^g^	LiHMDS	3.0	2.4	–	64

a Sulfoxide **6** added to base followed by pyrone **11a** with a final concentration of **6** of 0.042 M unless otherwise noted.

b 100 equiv.

c 5.6 equiv.

d 3.0 equiv.

e 3.3 equiv.

f Sulfoxide **6** added to base and pyrone **11a**.

g Concentration increased 1.8-fold.

**Scheme 3:**
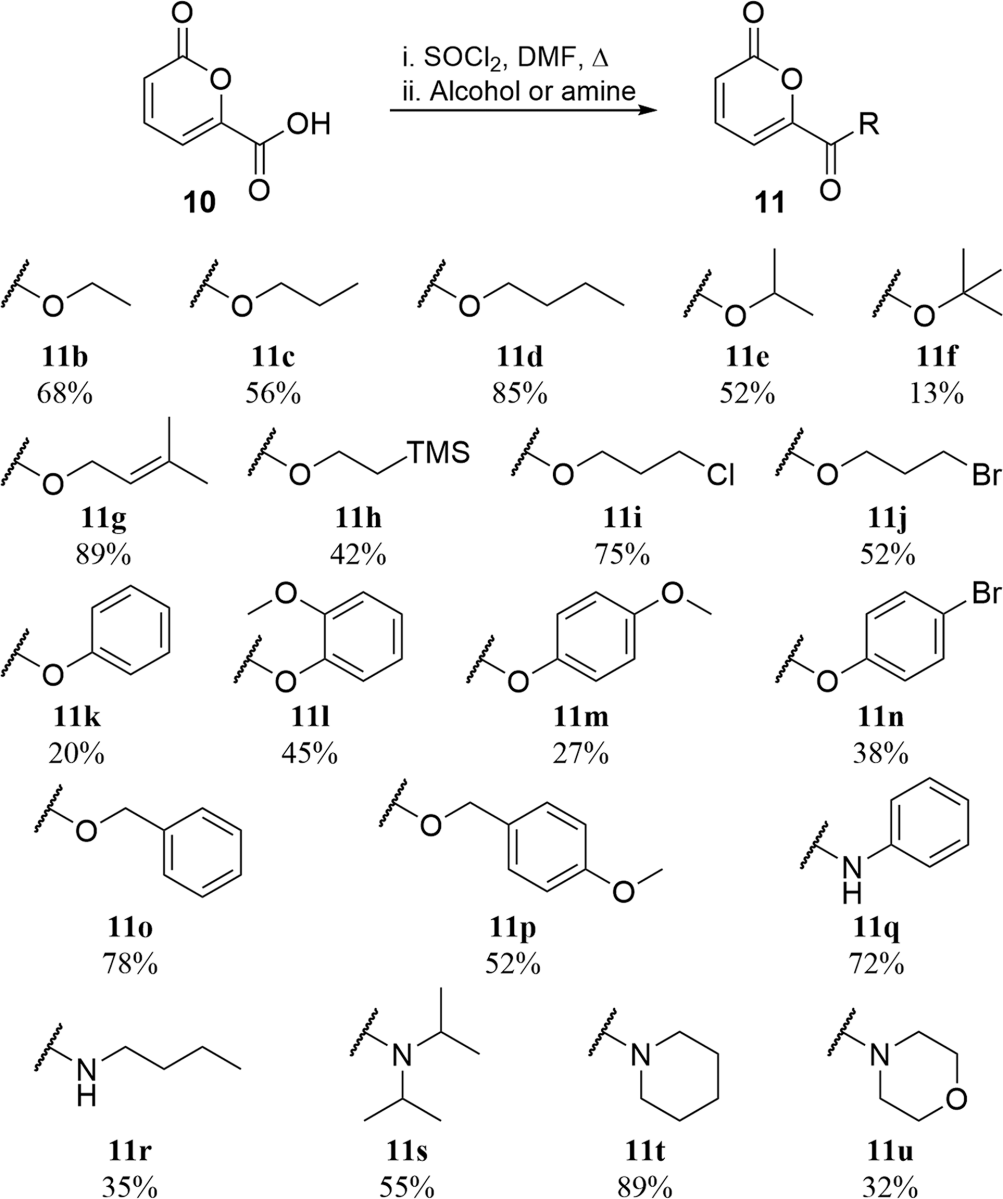
Synthesis of Other Pyrone Esters and Amides

Other pyrone esters could be made from **10** (Scheme 3) in an analogous manner to **11a**. Generation of alkyl esters **11b**–**11f** by reaction of the acid chloride with the corresponding alcohol generally proceeded well, except for with sterically bulky *tert*-butanol, as did alkene (**11g**), silyl (**11h**), and halogen (**11i** and **11j**) containing materials. Aryl (**11k**–**11n**) and aryl-containing (**11o** and **11p**) esters were also synthesized in reasonable yields. The addition of primary and secondary amines to the acid chloride of **10** in place of alcohols furnished the corresponding secondary aromatic (**11q**) and aliphatic (**11r**) amides as well as tertiary amides **11s**–**11u**.

**Scheme 4:**
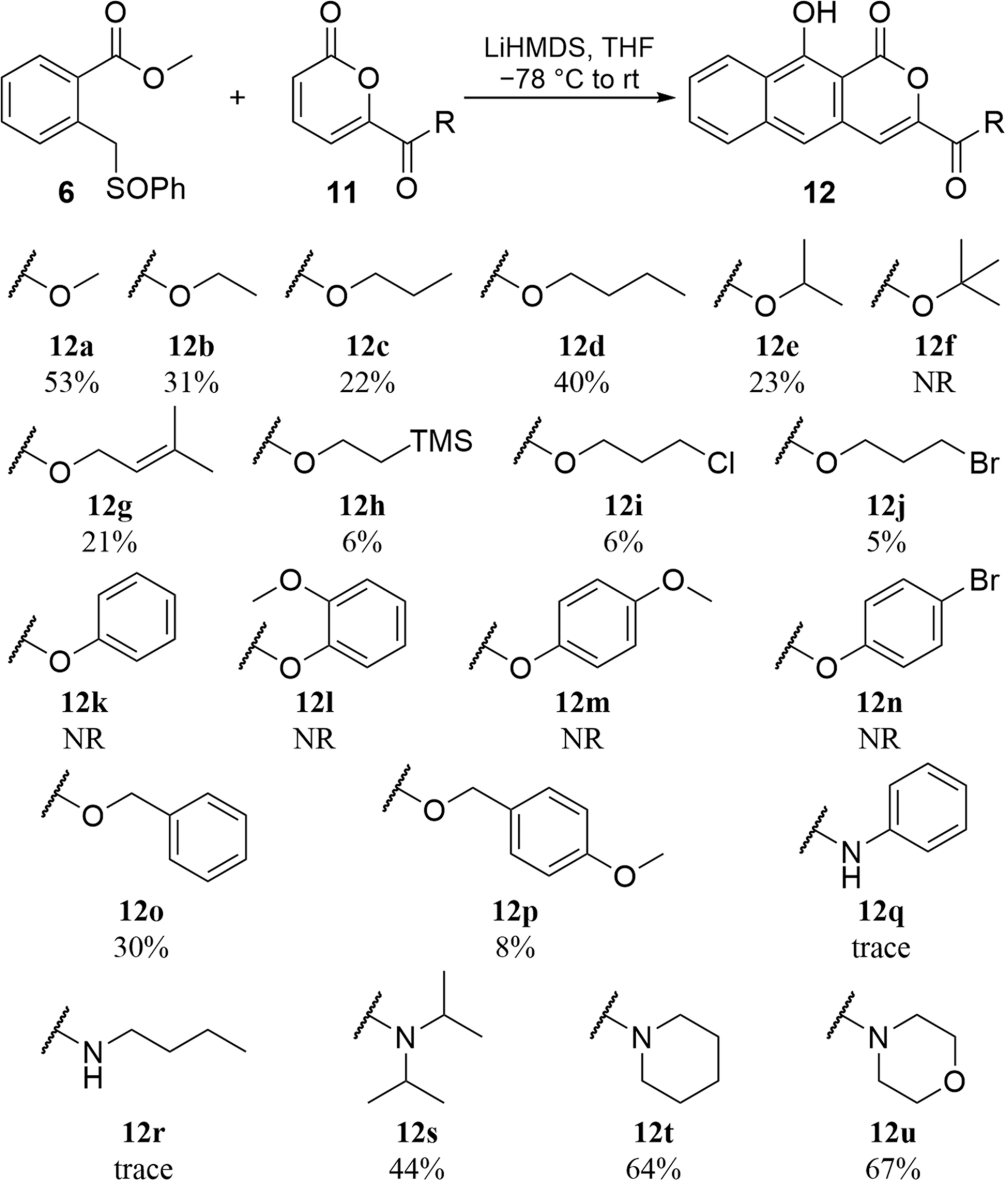
Isolated Annulation Yields with Various Pyrones

Application of the optimized annulation conditions to alkyl pyrone esters was generally effective, furnishing **12a**–**12e** (Scheme 4) in 22–53% isolated yields. The more sterically hindered *tert*-butyl pyrone **11f** did not react. Lower yields resulted from difficulties with chromatographic separation of the naphthyl-pyrones from closely eluting byproducts, an issue compounded by their low solubility in most organic solvents. Transesterification also complicated purification. The equivalent of methoxy produced upon the reaction of the pyrone enolate with the sulfoxide (Figure 2, **B** to **D**) in some cases reacted with the desired annulated products (**12**), converting them into **12a**. These could be separated by high-performance liquid chromatography (HPLC), but yields suffered accordingly. Despite the formation of this unwanted product, a variety of functional groups including an exogenous alkene (**12g**), a silyl group (**12h**), and halogens (**12i** and **12j**) proceeded through annulation, though required HPLC purification. For aryl esters, no appreciable amount of **12k**–**12n** could be isolated, presumably due to transesterification. Aromatic ester functionality in the form of benzyl esters worked, but products **12o** and **12p** required HPLC purification to separate them from **12a**. Production of secondary amide products **12q** and **12r** was detected, but due to deprotonation of the nitrogen and corresponding consumption of needed base, low yields precluded isolation and characterization, a problem that could conceivably be overcome by protection. Tertiary amides performed well (**12s**–**12u**) with yields equivalent to that of the optimized methyl ester. These results show that pyrones with a variety of C6-carbonyl substitutions are tolerated as dienophiles using this annulation approach.

**Scheme 5:**
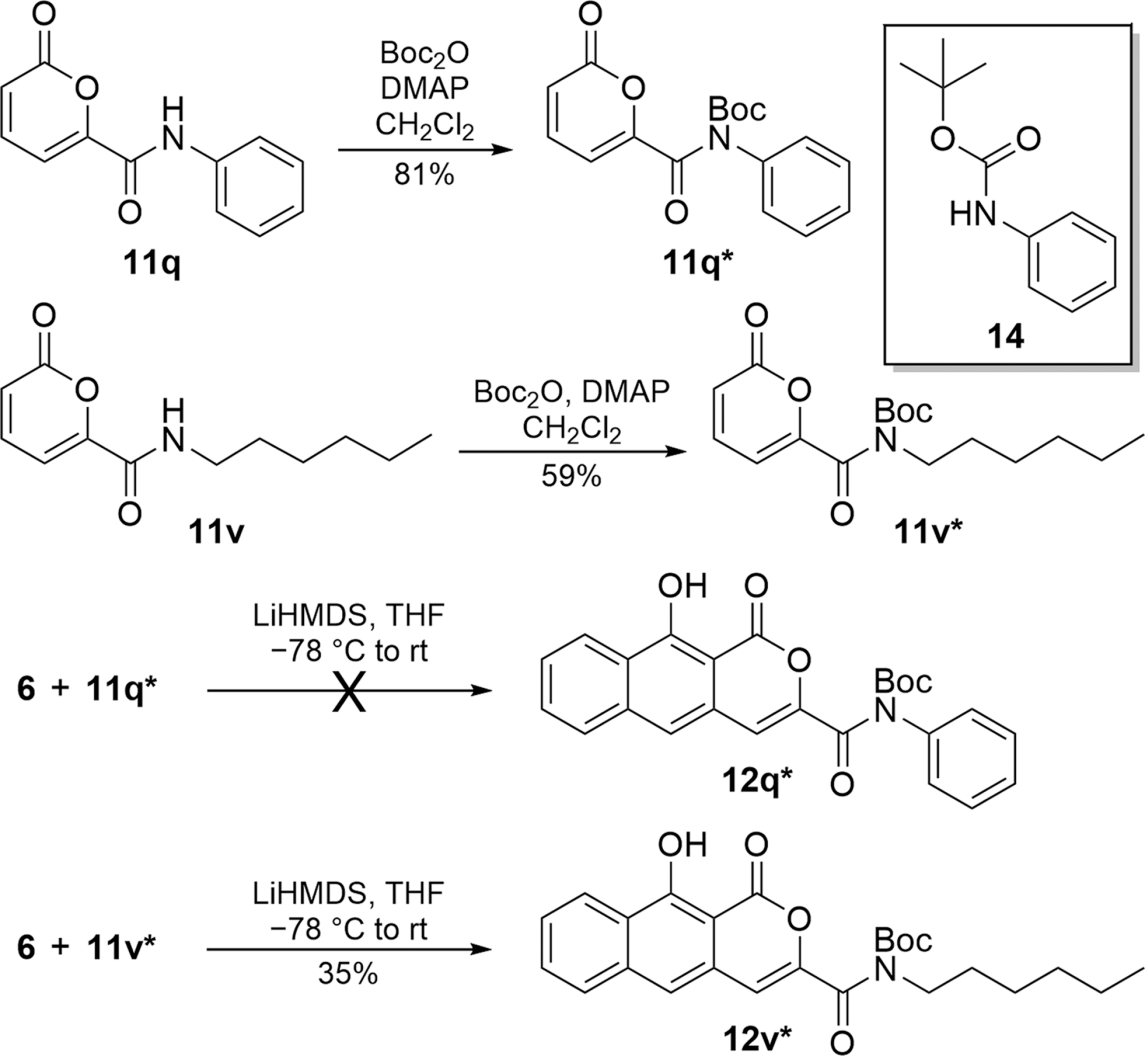
Synthesis and Testing of Protected Secondary Amide Pyrones

To investigate issues around low-yielding annulations and gain additional insight into reaction scope, protected pyrones (11*), and additional sulfoxide variants were prepared. A BOC-protected analogue (11q*, Scheme 5) of aniline amide **11q** was prepared, as was the BOC-protected variant (11v*) of the secondary hexyl amide **11v**, an alkyl amide analogous to **11r** selected because of its larger size. Unexpectedly, 11q* did not annulate. In this reaction, pyrone 11q* was completely consumed and BOC-protected aniline^28^ (**14**, *inset*) was isolated. This result stems from the stability of **14** as a leaving group (an effective p*K*_a_ of ∼10); LiHMDS must react with 11q*, similar to the decomposition of **11a** observed with LDA. In the case of protected alkyl amide 11v* however, the expected product 12v* was obtained.

**Scheme 6:**
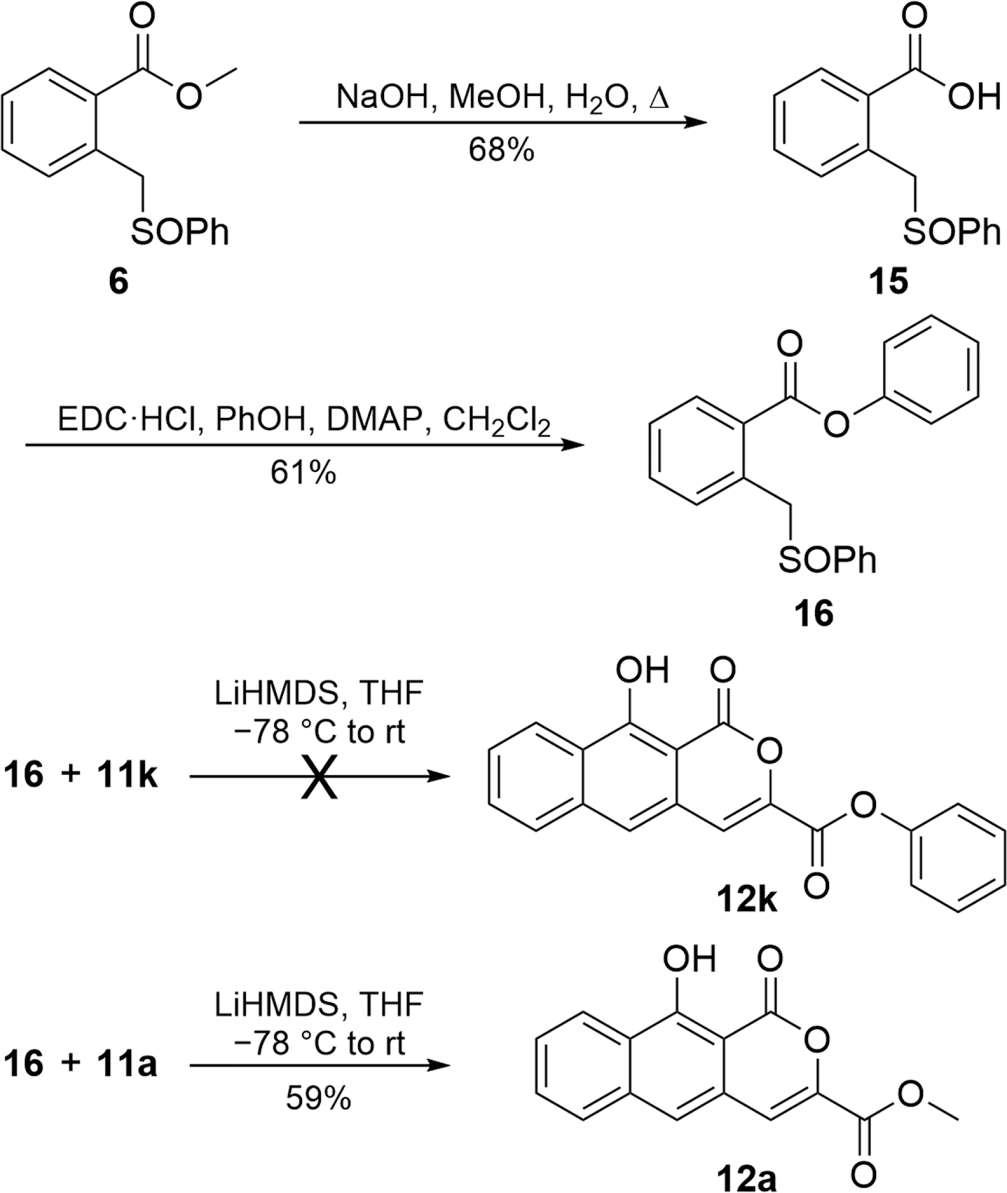
Preparation and Use of Sulfoxide Phenyl Ester **16** with Selected Pyrones

To test if transesterification could be suppressed by matching the leaving group of the sulfoxide ester with the esters of pyrones **11k**–**11n**, we prepared an alternative sulfoxide, swapping a phenyl ester (**16**, Scheme 6) for the methyl ester of **6**. This was accomplished by hydrolyzing **6** to its acid (**15**) and subsequently using peptide coupling conditions with phenol to create **16**. Reaction of **16** with **11k** under annulation conditions did not produce **12k**. Starting sulfoxide **16** was recovered while **11k** was not. Combined with the findings from BOC-protected pyrone variant 11q*, the inability of **11k** to annulate suggests a sufficiently labile leaving group off of the C6′ carbaldehyde precludes successful annulation; the base instead degrades the pyrone. However, a control reaction, where **16** was reacted with **11a**, produced **12a** in the highest isolated yield along with recovered **11a**. This result shows that generating and using the more costly phenyl ester (**16**) instead of **6**—which produces the less basic phenolate ion in lieu of the methoxide during **C** to **D** (Figure 2)—increases yields by suppressing unwanted reactions resulting from free methoxide, such as transesterification and ring-opening of **11a** to **13**.

α-Pyrone was also tested in the annulation reaction, but poor solubility and similar polarity profiles of the resulting compounds prevented complete separation and characterization. Two major masses consistent with the product were identified from the purified reaction mixture, suggesting that in the absence of C6-substitution to enforce regiochemistry, annulation can occur at either the α–β or γ–δ positions. A quaternary carbon also appears to be necessary off the C6 position. When tested, 2,3-dimethyl-4*H*-pyran-4-one did not undergo annulation, likely because of competitive deprotonation of the methyl group, which is made acidic due to conjugation with the ester.

**Scheme 7:**
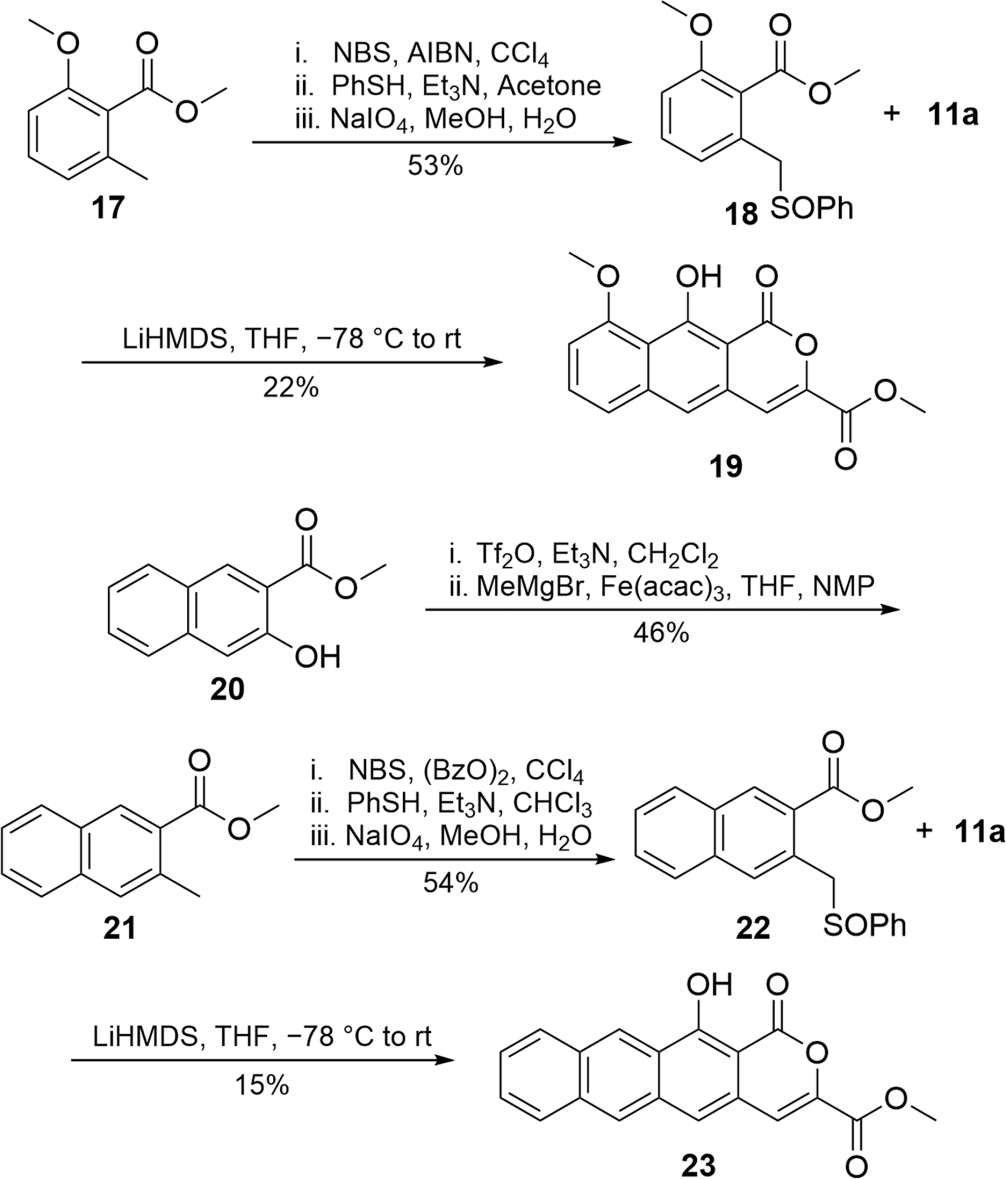
Creation and Annulation of Sulfoxide Derivatives **18** and **22**

To assess the applicability of this method toward thermorubin production, we sought to use ring systems more applicable to the natural product in the annulation with **11a** (Scheme 7). Application of a radical bromination, thioetherification, and oxidation sequence^22^ to methoxy benzoate **17**, as was used to produce **6**, gave sulfoxide **18** in good yield. Annulation with **11a** furnished **18** in 22% yield. To test a complete ring system, hydroxynaphthoate **20** was activated as the triflate and nucleophilic aromatic substitution with methylmagnesium bromide produced **21**, a naphthalene analogous to methyl toluate **3**. Application of an identical radical bromination, thioetherification, and oxidation sequence^22^ gave naphthoate sulfoxide **22** in good yield. The use of **22** with **11a** under reaction conditions optimized for **6** yielded anthracenepyrone **23**. These results successfully demonstrate that functionalized and larger ring systems are achievable using pyrone dienophiles. However, the yield in these cases was low, indicating that optimization will be required for this step during the natural product campaign.

## Conclusions

Despite their extensive presence in drugs and natural products, the synthesis of fused heterocycles is a challenging process that would benefit from additional methods for their formation. Here, we show that instead of creating an annulated pyrone from suitably functionalized aromatics^3,4,10^ it is possible to anneal this intact heterocycle onto existing rings in the absence of a guiding^6^ functionality. This annulation is selective for the α–β double bond of the pyrone when a carbonyl is present at the C6 position. The presence of the electron-withdrawing carbonyl at the δ position did not affect regiochemistry, likely because cross-conjugation from the pyrone ring oxygen diminishes the electron-withdrawing effect of the *exo*-ester and the enforced *syn* geometry of the pyrone enhances its electron-withdrawing ability. Although acidity issues prevented us from conclusively demonstrating annulation with alkyl substituents at C6, it is likely that this same regioselectivity would be observed with this substitution in other reactions when a milder base can be used or the C6 substituent is quaternary. When only a hydrogen atom was present at C6, however, rearomatization after attachment to the γ–δ position was enabled, and a mixture consistent with both regioisomers was observed. Conversion of methyl ester sulfoxide diene surrogate to its corresponding phenyl ester reduced the impact of deleterious side reactions involving liberated methoxide. Substituted and polycyclic sulfoxides were also suitable reactants. In summary, these findings show that pyrones containing C6-carbonyl groups are efficient selective dienophiles in sulfoxide-type annulations to polycycles and may be suitable in other cycloaddition reactions. This method shows that intact pyrone rings can be incorporated into more complex systems. Application of this approach to the synthesis of thermorubin and other pyrone-containing natural products is ongoing.

## Experimental Section

### General Experimental

Chemical reagents and solvents were purchased from EMD Millipore, Oakwood Chemical, Sigma Aldrich, Beantown Chemical, Acros, and Thermo Fisher Scientific. Unless otherwise specified, all nonaqueous reactions were carried out under an atmosphere of dry nitrogen in dried glassware. Commercially available starting materials and reagents were used as received or purified prior to use if necessary. Anhydrous^29^ THF was obtained commercially or from a solvent purification system.^30^ Diisopropylamine and triethylamine were distilled from calcium hydride. *n*BuLi was titrated using 3,5-di-*tert*-butyl-4-hydroxytoluene in THF using fluorene as an indicator. Analytical thin-layer chromatography was performed using Supelco 0.25 mm silica gel 60 F_254_ plates. Visualization was accomplished by irradiation with a 254 nm UV lamp or by staining with a basified aqueous solution of potassium permanganate. Chromatography was performed using a forced flow of the indicated solvent system on SiliCycle SiliaFlash P60 silica gel or prepacked commercial columns. Deionized water was obtained from the in-house water deionizing system.

^1^H NMR spectra were recorded on a Bruker Avance II 500 MHz spectrometer or an Agilent U4-DD2 400 MHz spectrometer. Chemical shifts are reported in parts per million from tetramethylsilane (0 ppm) using solvent resonance as an internal standard (CDCl_3_ 7.26 ppm, CD_3_OD 3.31 ppm). Data are reported as follows: chemical shift, multiplicity (s = singlet, d = doublet, t = triplet, q = quartet, p = pentet, m = multiplet, br = broad), coupling constant, and number of protons. Proton decoupled ^13^C NMR spectra were recorded on a Bruker Avance II 500 MHz (126 MHz) spectrometer, an Agilent U4-DD2 400 MHz (101 MHz) spectrometer, or a Bruker Avance III 600 MHz (151 MHz) spectrometer. Chemical shifts are reported in ppm from tetramethylsilane (0 ppm) using solvent resonance as an internal standard (CDCl_3_ 77.2 ppm, CD_3_OD 49.0 ppm). High-resolution mass spectra were obtained on an Agilent Technologies 6220 TOF LC/MS or a Waters Synapt Q-TOF G2 at the Department of Chemistry and the VT-Mass Spectrometry Incubator at the Virginia Polytechnic Institute and State University.

### Methyl 2-((Phenylsulfinyl)methyl)benzoate (**6**)

The following reactions were carried out in an analogous manner to the published procedure.^22^**Caution!** Carbon tetrachloride is highly toxic and should be handled exclusively in a fume cabinet to avoid vapor exposure. *N*-Bromosuccinimide (14.3 g, 80.1 mmol) and benzoyl peroxide (0.97 g, 4.0 mmol) were combined in a flame-dried flask under nitrogen, and CCl_4_ (100 mL) was added. Methyl *O*-toluate (**3**, 12.0 g, 80.0 mmol) was added, and the mixture was heated to reflux (oil bath) and stirred for 2 h. After cooling to rt, the mixture was filtered and the solid was washed with CCl_4_ (25 mL). The filtrate was concentrated to yield methyl 2-(bromomethyl)benzoate (**4**) as a yellow oil that was used directly without further purification.

Compound **4** was dissolved in CHCl_3_ (25 mL) and added to a stirring solution of PhSH (8.5 mL, 83.3 mmol) and K_2_CO_3_ (14.2 g, 103 mmol) in CHCl_3_ (75 mL). After stirring overnight, the mixture was diluted with Et_2_O (100 mL) and washed sequentially with solutions of aqueous NaOH (1 M, 50 mL), water (50 mL), and brine (50 mL). Concentration of the organic layer resulted in methyl 2-((phenylthiol)methyl)benzoate (**5**) that was used directly without further purification.

Compound **5** was dissolved in methanol (170 mL) and water (26 mL) and NaIO_4_ (17.8 g, 83.3 mmol) was added portion-wise. After stirring for 18 h, the mixture was diluted with water (100 mL) and EtOAc (200 mL), and the layers were separated. The organic layer was washed with water (2 × 50 mL) and brine (50 mL), dried using Na_2_SO_4_, and concentrated. The residue was purified using flash chromatography (40% EtOAc/hexanes, SiO_2_) to yield **6** (12.8 g, 58%) as an amorphous white solid. Spectral data were in accord with those previously reported.^22^

### 6-Ethyl 1-Methyl (2*Z*,4*E*)-2-Hydroxyhexa-2,4-dienedioate (**9**)

To a flame-dried flask was added *t*-BuOK (24.7 g, 0.220 mol) and Et_2_O (86 mL). The flask was purged with nitrogen, and after the mixture had cooled to 0 °C (cryocool), diethyl oxalate (27.1 mL, 0.200 mol) dissolved in Et_2_O (16 mL) was added dropwise over 15 min, followed by ethyl crotonate (24.9 mL, 0.200 mol) in a dropwise fashion. The mixture was stirred at 4 °C (cryocool) overnight, after which the reaction was filtered, and the precipitate was washed with Et_2_O (100 mL). The yellow-orange precipitate was dissolved in cold water (750 mL) and 50% aqueous acetic acid (35 mL) was added. Filtration (water) and drying of the precipitate resulted in dienedioate **9** (30 g, 75%) as an amorphous yellow solid. Spectral data were in accord with those previously reported.^24^

### 2-Oxo-2*H*-pyran-6-carboxylic Acid (**10**)

Dienedioate **9** (11.65 g, 54.39 mmol) was dissolved in concentrated HCl (325 mL), heated to reflux (oil bath), and stirred for 8 h. The mixture was cooled to rt and then cooled to −20 °C (cryocool) for 4 h. The precipitate was collected by filtration and the filtrate was stored at −20 °C for 12 h, after which additional filtrate was collected. Drying yielded **10** (6.71 g, 88%) as an amorphous gold-yellow solid. Spectral data were in accord with those previously reported.^24^

### General Procedure for the Preparation of Pyrone Ester Derivatives (**11**)

The following reactions were carried out in an analogous manner to the published procedure.^23,24^ Pyrone carboxylic acid **10** (1 equiv) was dissolved in SOCl_2_ (10–20 equiv) and a catalytic amount of *N*,*N*-dimethylformamide (DMF, 0.05 equiv) was added. The reaction was heated to reflux (oil bath) and stirred for 15 h, after which excess solvent was removed via distillation. The resulting acid chloride was dissolved in the appropriate alcohol (1–5 equiv) and the mixture was stirred at rt for 1 h. The resulting solid material was either filtered and the filtrate concentrated to give **11** or dissolved in CH_2_Cl_2_, washed with saturated aqueous NaHCO_3_, dried (Na_2_SO_4_), concentrated, and the residue purified using flash chromatography (SiO_2_) to yield **11**.

### Methyl 2-Oxo-2*H*-pyran-6-carboxylate (**11a**)

Purified by filtration to give **11a** (0.516 g, 91%) as an amorphous white solid: ^1^H NMR (400 MHz, CDCl_3_) δ 7.41 (dd, *J* = 9.4, 6.6 Hz, 1H), 7.10 (dd, *J* = 6.5, 1.0 Hz, 1H), 6.55 (dd, *J* = 9.4, 1.0 Hz, 1H), 3.94 (s, 3H); ^13^C{^1^H} NMR (101 MHz, CDCl_3_) δ 159.8, 159.6, 149.4, 141.7, 121.1, 109.9, 53.1. HRMS (ESI) calcd for C_7_H_7_O_4_ [M + H]^+^ 155.0344, found 155.0343.

### Ethyl 2-Oxo-2*H*-pyran-6-carboxylate (**11b**)

Purified using CHCl_3_ to give **11b** (0.490 g, 68%) as an amorphous pink solid: ^1^H NMR (400 MHz, CDCl_3_) δ 7.41 (dd, *J* = 9.4, 6.5 Hz, 1H), 7.08 (dd, *J* = 6.6, 1.0 Hz, 1H), 6.52 (dd, *J* = 9.4, 1.0 Hz, 1H), 4.37 (q, *J* = 7.1 Hz, 2H), 1.37 (t, *J* = 7.1 Hz, 3H); ^13^C{^1^H} NMR (101 MHz, CDCl_3_) δ 159.9, 159.5, 149.8, 141.9, 121.0, 109.9, 62.7, 14.2; HRMS (ESI) calcd for C_8_H_9_O_4_ [M + H]^+^ 169.0501, found 169.0499.

### Propyl 2-Oxo-2*H*-pyran-6-carboxylate (**11c**)

Purified using CH_2_Cl_2_ to give **11c** (0.367 g, 56%) as a yellow amorphous solid: ^1^H NMR (400 MHz, CDCl_3_) δ 7.41 (dd, *J* = 9.4, 6.6 Hz, 1H), 7.04 (dd, *J* = 6.6, 1.2 Hz, 1H), 6.47 (dd *J* = 9.4, 1.2 Hz, 1H), 4.21 (dd, *J* = 7.1, 6.4 Hz, 2H), 1.70 (qt, *J* = 7.4, 6.7 Hz, 2H), 0.92 (t, *J* = 7.4 Hz, 3H); ^13^C{^1^H} NMR (101 MHz, CDCl_3_) δ 160.0, 159.6, 149.8, 141.9, 121.0, 109.9, 68.2, 22.0, 10.4; HRMS (ESI) calcd for C_9_H_11_O_4_ [M + H]^+^ 183.0657, found 183.0658.

### Butyl 2-Oxo-2*H*-pyran-6-carboxylate (**11d**)

Purified using a 2–4% EtOAc/CH_2_Cl_2_ to give **11d** (0.596 g, 85%) as a yellow-white amorphous solid: ^1^H NMR (400 MHz, CDCl_3_) δ 7.41 (dd, *J* = 9.4, 6.6 Hz, 1H), 7.09 (dd, *J* = 6.5, 1.0 Hz, 1H), 6.54 (dd, *J* = 9.4, 1.0 Hz, 1H), 4.33 (t, *J* = 6.7 Hz, 2H), 1.79–1.67 (m, 2H), 1.51–1.35 (m, 2H), 0.96 (t, *J* = 7.4 Hz, 3H); ^13^C{^1^H} NMR (101 MHz, CDCl_3_) δ 160.0, 159.6, 149.9, 141.9, 121.0, 109.9, 66.5, 30.6, 19.2, 13.8; HRMS (ESI) calcd for C_10_H_13_O_4_ [M + H]^+^ 197.0814, found 197.0814.

### Isopropyl 2-Oxo-2*H*-pyran-6-carboxylate (**11e**)

Purified using 3% acetone/PhH to give **11e** (0.35 g, 52%) as a white amorphous solid: ^1^H NMR (400 MHz, CDCl_3_) δ 7.41 (dd, *J* = 9.4, 6.6 Hz, 1H), 7.07 (d, J= 7.0 Hz, 1H), 6.53 (d, *J* = 9.4 Hz, 1H), 5.23 (heptet, *J* = 6.3 Hz, 1H), 1.36 (d, *J* = 6.3 Hz, 6H); ^13^C{^1^H} NMR (126 MHz, CDCl_3_) δ 160.3, 159.2, 150.4, 142.2, 121.1, 110.0, 71.1, 22.1; HRMS (ESI) calcd for C_9_H_11_O_4_ [M + H]^+^ 183.0657, found 183.0657

### *tert*-Butyl 2-Oxo-2*H*-pyran-6-carboxylate (**11f**)

Purified using CHCl_3_ to give **11f** (0.051 g, 13%) as an orange amorphous solid: ^1^H NMR (400 MHz, CDCl_3_) δ 7.39 (dd, *J* = 9.4, 6.6 Hz, 1H), 7.01 (dd, *J* = 6.6, 1.0 Hz, 1H), 6.50 (dd, *J* = 9.4, 1.1 Hz, 1H) 1.56 (s, 9H); ^13^C{^1^H} NMR (101 MHz, CDCl_3_) δ 160.3, 158.4, 150.9, 142.0, 120.6, 109.2, 84.2, 28.1; HRMS (ESI) calcd for C_10_H_12_O_4_Na [M + Na]^+^ 219.0633, found 219.0628.

### 3-Methylbut-2-en-1-yl 2-Oxo-*2H*-pyran-6-carboxylate (**11g**)

Purified using CH_2_Cl_2_ to give **11g** (0.251 g, 89%) as an orange amorphous solid: ^1^H NMR (400 MHz, CDCl_3_) δ 7.40 (dd, *J* = 9.4, 6.6 Hz, 1H), 7.07 (d, *J* = 6.5 Hz, 1H), 6.51 (d, *J* = 9.4 Hz, 1H), 5.45–5.38 (m, 1H), 4.79 (d, *J* = 7.4 Hz, 2H), 1.78 (s, 3H), 1.75 (s, 3H); ^13^C{^1^H} NMR (101 MHz, CDCl_3_) δ 159.9, 159.5, 149.9, 141.9, 140.9, 120.9, 117.6, 109.9, 63.3, 25.9, 18.2; HRMS (ESI) calcd for C_11_H_12_O_4_Na [M + Na]^+^ 231.0633, found 231.0626.

### 2-(Trimethylsilyl)ethyl 2-Oxo-2*H*-pyran-6-carboxylate (**11h**)

Purified using CHCl_3_ to give **11h** (0.192 g, 42%) as a pale-white amorphous solid: ^1^H NMR (400 MHz, CDCl_3_) δ 7.40 (dd, *J* = 9.4, 6.6 Hz, 1H), 7.09–7.06 (m, 1H), 6.55–6.51 (m, 1H), 4.46–4.38 (m, 2H), 1.16–1.09 (m, 2H), 0.08 (s, 9H); ^13^C{^1^H} NMR (101 MHz, CDCl_3_) δ 159.9, 159.6, 150.1, 141.9, 121.0, 109.7, 65.2, 17.5, −1.4; HRMS (ESI) calcd for C_11_H_16_O_4_NaSi [M + Na]^+^ 263.0716, found 263.0724.

### 3-Chloropropyl 2-Oxo-*2H*-pyran-6-carboxylate (**11i**)

Purified using a 0–15% acetone/CHCl_3_ to give **11i** (0.324 g, 75%) as a red-white amorphous solid: ^1^H NMR (400 MHz, CDCl_3_) δ 7.43 (dd, *J* = 9.4, 6.6 Hz, 1H), 7.12 (dd, *J* = 6.6, 1.0 Hz, 1H), 6.56 (dd, *J* = 9.4, 1.0 Hz, 1H), 4.50 (t, *J* = 6.1 Hz, 2H), 3.68 (t, *J* = 6.3 Hz, 2H), 2.31–2.15 (m, 2H); ^13^C{^1^H} NMR (101 MHz, CDCl_3_) δ 159.8, 159.4, 149.5, 141.8, 121.3, 110.2, 63.3, 41.0, 31.4; HRMS (ESI) calcd for C_9_H_10_ClO_4_ [M + H]^+^ 217.0268, found 217.0267.

### 3-Bromopropyl 2-Oxo-*2H*-pyran-6-carboxylate (**11j**)

Purified using 90% CH_2_Cl_2_/hexanes, then CH_2_Cl_2_ to give **11j** (0.53 g, 52%) as an amorphous white solid: ^1^H NMR (400 MHz, CDCl_3_) δ 7.47–7.38 (m, 1H), 7.11 (ddt, *J* = 6.6, 2.5, 1.0 Hz, 1H), 6.55 (ddt, *J* = 9.4, 2.6, 1.0 Hz, 1H), 4.52–4.39 (m, 2H), 3.58–3.44 (m, 2H), 2.36–2.25 (m, 2H); ^13^C{^1^H} NMR (126 MHz, CDCl_3_) δ 159.5, 159.1, 149.2, 141.6, 121.0, 110.0, 64.0, 31.2, 28.9; HRMS (ESI) calcd for C_9_H_10_BrO_4_ [M + H]^+^ 260.9762, found 260.9760.

### Phenyl 2-Oxo-2*H*-pyran-6-carboxylate (**11k**)

Purified using 0–10% acetone/CHCl_3_ to give **11k** (0.090 g, 20%) as a white amorphous solid: ^1^H NMR (400 MHz, CDCl_3_) δ 7.48 (dd, *J* = 9.4, 6.6 Hz, 1H), 7.45–7.40 (m, 2H), 7.32–7.29 (m, 1H), 7.27 (dd, *J* = 6.6, 1.0 Hz, 1H), 7.22–7.17 (m, 2H), 6.62 (dd, *J* = 9.4, 1.0 Hz, 1H); ^13^C{^1^H} NMR (101 MHz, CDCl_3_) δ 159.5, 157.9, 150.0, 149.0, 141.6, 129.7, 126.6, 121.6, 121.2, 111.0; HRMS (ESI) calcd for C_12_H_9_O_4_, [M + H]^+^ 217.0495, found 217.0494.

### 2-Methoxyphenyl 2-Oxo-2*H*-pyran-6-carboxylate (**11l**)

Purified using 0–15% acetone/CHCl_3_ to give **11l** (0.239 g, 45%) as a white amorphous solid: ^1^H NMR (400 MHz, CDCl_3_) δ 7.47 (dd, *J* = 9.4, 6.6 Hz 1H), 7.29–7.24 (m, 2H), 7.12 (dd, *J* = 8.0, 1.5 Hz 1H), 7.04–6.96 (m, 2H), 6.61 (dd, *J* = 9.4, 1.0 Hz, 1H), 3.83 (s, 3H); ^13^C{^1^H} NMR (101 MHz, CDCl_3_) δ 159.7, 157.4, 151.0, 149.1, 141.7, 139.1, 127.8, 122.6, 121.7, 121.0, 112.8, 111.1, 56.0; HRMS (ESI) calcd for C_13_H_11_O_5_ [M + H]^+^ 247.0606, found 247.0608.

### 4-Methoxyphenyl 2-Oxo-2*H*-pyran-6-carboxylate (**11m**)

Purified using 75–100% CHCl_3_/hexanes to give **11m** (0.151 g, 27%) as an off-white amorphous solid: ^1^H NMR (400 MHz, CDCl_3_) δ 7.47 (dd, *J* = 9.5, 6.5 Hz, 1H), 7.26 (dd, *J* = 6.5, 1.1 Hz, 1 H) 7.15–7.07 (m, 2H), 6.97–6.89 (m, 2H), 6.62 (dd, *J* = 9.4, 1.0 Hz, 1H), 3.82 (s, 3 H); ^13^C{^1^H} NMR (101 MHz, CDCl_3_) δ 159.7, 158.3, 157.9, 149.3, 143.6, 141.8, 122.1, 121.7, 114.8, 111.0, 55.8; HRMS (ESI) calcd for C_13_H_11_O_5_ [M + H]^+^ 247.0606, found 247.0604.

### 4-Bromophenyl 2-Oxo-2*H*-pyran-6-carboxylate (**11n**)

Purified using 0–25% EtOAc/CHCl_3_ to give **11n** (0.154 g, 38%) as a white, amorphous solid: ^1^H NMR (400 MHz, CDCl_3_) δ 7.56–7.51 (m, 2H), 7.47 (dd, *J* = 9.4, 6.6 Hz, 1H), 7.26 (dd, *J* = 6.6, 1.0 Hz, 1H), 7.12–7.06 (m, 2H), 6.61 (dd, *J* = 9.4, 1.0 Hz, 1H); ^13^C{^1^H} NMR (101 MHz, CDCl_3_) δ 159.3, 157.6, 149.0, 148.7, 141.5, 132.8, 123.0, 121.8, 119.8, 111.2; HRMS (ESI) calcd for C_12_H_8_BrO_4_, [M + H]^+^ 294.9600, found 294.9592.

### Benzyl 2-Oxo-*2H*-pyran-6-carboxylate (**11o**)

Purified using 80% CH_2_Cl_2_/hexanes to give **11o** (0.640 g, 78%) as a white amorphous solid: ^1^H NMR (400 MHz, CDCl_3_) δ 7.53–7.31 (m, 6H), 7.11 (d, *J* = 6.6 Hz, 1H), 6.54 (d, *J* = 9.4 Hz, 1H), 5.35 (s, 2H); ^13^C{^1^H} NMR (101 MHz, CDCl_3_) δ 159.8, 159.3, 149.5, 141.8, 134.7, 128.9, 128.84, 128.76, 121.2, 110.2, 68.2; HRMS (ESI) calcd for C_13_H_11_O_4_ [M + H]^+^ 231.0657, found 231.0665.

### 4-Methoxybenzyl 2-Oxo-*2H*-pyran-6-carboxylate (**11p**)

Isolated by filtration and concentration to give **11p** (0.43 g, 52%) as an orange amorphous solid: ^1^H NMR (400 MHz, CDCl_3_) δ 7.42–7.34 (m, 1H), 7.38–7.35 (m, 2H) 7.09 (dd, *J* = 6.5, 1.0 Hz, 1H), 6.93–6.87 (m, 2H), 6.53 (dd, *J* = 9.4, 1.0 Hz, 1H), 5.28 (s, 2H), 3.81 (s, 3H). ^13^C{^1^H} NMR (151 MHz, CDCl_3_) δ 160.1, 159.9, 159.4, 149.6, 141.9, 130.8, 126.8, 121.1, 114.2, 110.15, 68.1, 55.4; HRMS (ESI) calcd for C_14_H_12_NaO_5_ [M + Na]^+^ 283.0577, found 283.0576.

### 2-Oxo-*N*-phenyl-2*H*-pyran-6-carboxamide (**11q**)

Isolated by filtration and concentration to give **11q** (0.56 g, 72%) as a white amorphous solid: ^1^H NMR (400 MHz, CDCl_3_) δ 8.51 (br s, 1H), 7.69–7.65 (m, 2H), 7.52 (dd, *J* = 9.4, 6.6 Hz, 1H), 7.43–7.37 (m, 2H), 7.25 (dd, *J* = 6.6, 1.0 Hz, 1H), 7.23–7.18 (m, 1H), 6.54 (dd, *J* = 9.4, 1.0 Hz, 1H); ^13^C{^1^H} NMR (126 MHz, CDCl_3_) δ 159.8, 156.3, 152.6, 143.4, 136.8, 129.6, 125.8, 120.7, 119.8, 107.7; HRMS (ESI) calcd for C_12_H_10_NO_3_ [M + H]^+^ 216.0661, found 216.0679.

### *N*-Butyl-2-oxo-2*H*-pyran-6-carboxamide (**11r**)

Purified using 20% EtOAc/CH_2_Cl_2_ to give **11r** (0.25 g, 35%) as a brown amorphous solid: ^1^H NMR (400 MHz, CDCl_3_) δ 7.46 (ddd, *J* = 9.4, 6.6, 0.5 Hz, 1H), 7.12 (dd, *J* = 6.6, 1.0 Hz, 1H), 6.84 (s, 1H), 6.48 (dd, *J* = 9.4, 1.1 Hz, 1H), 3.41 (td, *J* = 7.1, 5.9 Hz, 2H), 1.67–1.51 (m, 2H), 1.46–1.31 (m, 2H), 0.95 (t, *J* = 7.3 Hz, 3H); ^13^C{^1^H} NMR (126 MHz, CDCl_3_) δ 159.9, 158.3, 152.6, 143.2, 119.1, 106.7, 39.6, 31.5, 20.1, 13.8; HRMS (ESI) calcd for C_10_H_14_NO_3_ [M + H]^+^ 196.0974, found 196.0970.

### *N*,*N*-Diisopropyl-2-oxo-2*H*-pyran-6-carboxamide (**11s**)

Purified using CH_2_Cl_2_, then 0.25% MeOH/CH_2_Cl_2_, then 0.5% MeOH/CH_2_Cl_2_ to give **11s** (0.44 g, 55%) as an amorphous off-white solid: ^1^H NMR (400 MHz, CDCl_3_) δ 7.37 (dd, *J* = 9.6, 6.7 Hz, 1H), 6.44 (d, *J* = 6.6 Hz, 1H), 6.33 (d, *J* = 9.7 Hz, 1H), 3.84 (br s, 1H), 3.51 (br s, 1H), 1.45 (br s, 6H), 1.26 (br s, 6H); ^13^C{^1^H} NMR (126 MHz, CDCl_3_) δ 161.1, 160.1, 157.7, 143.2, 116.8, 104.8, 51.1, 46.5, 20.8, 20.1. HRMS (ESI) calcd for C_12_H_18_NO_3_ [M + H]^+^ 224.1287, found 224.1287.

### 6-(Piperidine-1-carbonyl)-2*H*-pyran-2-one (**11t**)

Purified using 60% EtOAc/CH_2_Cl_2_ to give **11t** (0.67 g, 89%) as an amorphous orange solid: ^1^H NMR (400 MHz, CDCl_3_) δ 7.38 (dd, *J* = 9.4, 6.6 Hz, 1H), 6.58 (d, *J* = 6.6 Hz, 1H), 6.36 (d, *J* = 9.4 Hz, 1H), 3.61 (br s, 2H), 3.46 (br s, 2H) 1.72–1.59 (m, 6H); ^13^C{^1^H} NMR (126 MHz, CDCl_3_) δ 160.3, 160.1, 156.3, 143.1, 117.5, 106.7, 48.2, 44.0, 26.6, 25.6, 24.5; HRMS (ESI) calcd for C_11_H_14_NO_3_ [M + H]^+^ 208.0974, found 208.0967.

### 6-(Morpholine-4-carbonyl)-2*H*-pyran-2-one (**11u**)

Purified using EtOAc to give **11u** (0.074 g, 32%) as an amorphous white solid: ^1^H NMR (400 MHz, CD_3_OD) δ 7.58 (dd, *J* = 9.5, 6.6, 1H), 6.70 (dd, *J* = 6.6, 0.9 Hz, 1H), 6.44 (dt, *J* = 9.5, 0.9 Hz, 1H), 3.71 (br s, 4 H), 3.67 (br s, 4H); ^13^C{^1^H} NMR (151 MHz, CD_3_OD) δ 162.3, 161.9, 155.5, 144.9, 118.7, 108.5, 67.9, 67.5, 44.2; HRMS (ESI) calcd for C_10_H_12_NO_4_ [M + H]^+^ 210.0766, found 210.0765.

### *N*-Hexyl-2-oxo-2*H*-pyran-6-carboxamide (**11v**)

Purified using 20% EtOAc in CH_2_Cl_2_ to give **11v** (0.25 g, 63%) as a brown solid: ^1^H NMR (400 MHz, CDCl_3_) δ 7.46 (dd, *J* = 9.4, 6.6 Hz, 1H), 7.12 (dd, *J* = 6.6, 1.0 Hz, 1H), 6.87 (s, 1H), 6.48 (dd, *J* = 9.4, 1.0 Hz, 1H), 3.40 (td, *J* = 7.2, 6.0 Hz, 2H), 1.63–1.54 (m, 2H), 1.33–1.28 (m, 6H), 0.92–0.86 (m, 3H); ^13^C NMR (151 MHz, CDCl_3_) δ 159.8, 158.2, 152.5, 143.1, 119.1, 106.6, 39.8, 31.4, 29.3, 26.6, 22.5, 14.0; HRMS (ESI) calcd for C_12_H_18_NO_3_ [M + H]^+^ 224.1287, found 224.1290.

### *tert*-Butyl (2-Oxo-2*H-*pyran-6-carbonyl)(phenyl)carbamate (11q*)

To a stirring solution of **11q** (0.278 g, 1.29 mmol) in CH_2_Cl_2_ (15 mL) was added DMAP (17.0 mg, 0.139 mmol) and Boc_2_O (0.60 mL, 2.6 mmol). The mixture was stirred at rt for 24 h, quenched by the addition of saturated aqueous NH_4_Cl solution (10 mL), and the layers separated. The aqueous layer was extracted with CH_2_Cl_2_ (2 × 15 mL). The organic layers were combined, dried (Na_2_SO_4_), and concentrated. The residue purified was using flash chromatography (SiO_2_, CH_2_Cl_2_) to give 11q* as a light-brown amorphous solid (0.333 g, 81%): ^1^H NMR (400 MHz, CDCl_3_) δ 7.48–7.35 (m, 4H), 7.25–7.21 (m, 2H), 6.81 (dd, *J* = 6.6, 1.0 Hz, 1H), 6.49 (dd, *J* = 9.5, 1.0 Hz, 1H), 1.40 (s, 9H); ^13^C{^1^H} (101 MHz, CDCl_3_) δ 163.8, 159.4, 155.1, 152.1, 142.7, 137.6, 129. 5, 128.7, 128.1, 119.3, 107.4, 85.0, 27.8; HRMS (ESI) calcd for C_17_H_17_NO_5_Na [M + Na]^+^ 338.1004, found 338.1005.

### *tert*-Butyl Hexyl(2-oxo-2*H*-pyran-6-carbonyl)carbamate (11v*)

To a stirring solution of **11v** (0.294 g, 1.32 mmol) in CH_2_Cl_2_ (12 mL) was added DMAP (17.0 mg, 0.139 mmol), Et_3_N (0.25 mL, 1.794 mmol), and Boc_2_O (0.45 mL, 2.0 mmol). The mixture was stirred at rt for 24 h, quenched by the addition of saturated aqueous NH_4_Cl (6 mL), and the layers separated. The aqueous layer was extracted with CH_2_Cl_2_ (2 × 6 mL). The organic layers were combined, dried (Na_2_SO_4_), and concentrated. The residue was purified using flash chromatography (SiO_2_, 10–20% EtOAc/hexanes) to give 11v* as a brown oil (0.253 g, 59%): ^1^H NMR (400 MHz, CDCl_3_) δ 7.41 (dd, *J* = 9.4, 6.6 Hz, 1H), 6.66 (dd, *J* = 6.6, 1.0 Hz, 1H), 6.43 (dd, *J* = 9.4, 1.0 Hz, 1H), 3.73–3.66 (m, 2H), 1.66–1.54 (m, 2H), 1.42 (s, 9H), 1.36–1.26 (m, 6H), 0.92–0.86 (m, 3H); 13C{1H} NMR (101 MHz, CDCl_3_) δ 164.0, 159.6, 155.9, 152.2, 142.9, 118.6, 106.3, 84.5, 46.0, 31.5, 28.6, 27.8, 26.5, 22.7, 14.1; HRMS (ESI) calcd for C_17_H_25_NO_5_Na [M + Na]^+^ 346.1630, found 346.1614.

### General Procedure for Pyrone Annulation (**12**)

Hexamethyldisilane (1.2 mmol) was added to a round-bottom flask containing THF (0.2 mL). The mixture was placed in an ice bath, and *n*BuLi (1.1 mmol) was added dropwise. After the addition was complete, the mixture was stirred for 10 min, warmed to rt and stirred for 15 min, and then cooled to −78 °C (dry ice/acetone bath) for 15 min. Commercial 1 M LiHMDS solution in THF could be successfully used as well. Pyrone **11** (0.80 mmol) dissolved in THF (3 mL) was added and the mixture was stirred at −78 °C (dry ice/acetone bath) for 15 min. Sulfoxide **6** (0.37 mmol) dissolved in THF (0.8 mL) was added dropwise, and the mixture was stirred at −78 °C (dry ice/acetone bath) for 1 h. The mixture was slowly warmed to rt, and after stirring for 2 h, HCl (4 mL, 10% in water) was added. THF was removed under reduced pressure, and the residual aqueous solution was extracted with CHCl_3_ (3 × 4 mL). The combined organic layers were dried (Na_2_SO_4_) and concentrated. The residue was purified using automated flash chromatography (SiO_2_) to yield **12**. Because of the highly similar nature of the impurities (mainly **12a**), additional purification using HPLC was needed for some derivatives. These were performed using a Cogent Bidentate C18 column (100 Å, 4 μm, 250 mm × 10 mm) on a Shimadzu system equipped with a manual injector, CBM-20A communication bus module, DGU-20A degassing unit, LC-20AR liquid chromatography binary pump, SPD-20A UV/vis detector, and FRC-10A fraction collector with water containing 0.1% formic acid as solvent A and CH_3_CN containing 0.1% formic acid as solvent B.

### Methyl 10-Hydroxy-1-oxo-1*H*-benzo[*g*]isochromene-3-carboxylate (**12a**)

Purified using 75–100% CHCl_3_ in hexanes to give **12a** (0.093 g, 48%) as a yellow amorphous solid: ^1^H NMR (400 MHz, CDCl_3_) δ 12.14 (s, 1H), 8.49 (ddt, *J* = 8.4, 1.5, 0.8 Hz, 1H), 7.90–7.84 (m, 1H), 7.73 (ddd, *J* = 8.2, 6.9, 1.3 Hz, 1H), 7.62 (ddd, *J* = 8.2, 6.9, 1.2 Hz, 1H), 7.57 (s, 1H), 7.48 (d, *J* = 0.8 Hz, 1H), 3.98 (s, 3H); ^13^C{^1^H} NMR (126 MHz, CDCl_3_) δ 166.3, 162.2, 160.9, 141.4, 137.7, 131.2, 128.7, 128.2, 127.1, 124.6, 124.5, 117.6, 114.8, 100.7, 53.1. HRMS (ESI) calcd for C_15_H_11_O_5_ [M + H]^+^ 271.0601, found 271.0604.

### Representative Preparative Annulation to Produce **12a**

A flame-dried flask was charged with **11a** (0.808 g, 5.25 mmol) and THF (36 mL). The resulting stirring solution was purged with nitrogen and then cooled to −78 °C (dry ice/acetone bath) for 15 min. A solution of LiHMDS (6.6 mL, 6.6 mmol) was added dropwise over 5 min, and the resulting orange solution stirred for an additional 15 min. Sulfoxide **6** (0.598 g, 2.18 mmol) dissolved in THF (9 mL) was added dropwise over 10 min, and the solution was stirred for 45 min. The cooling bath was then removed, and the reaction warmed to rt over 1.5 h. After quenching with 10% aqueous HCl (25 mL), the mixture was extracted with CHCl_3_ (3 × 25 mL). The combined organic layers were washed with brine (25 mL), dried (Na_2_SO_4_), and concentrated. The resulting residue was purified using automated flash chromatography (SiO_2_, 0–2% EtOAc/CHCl_3_) to yield **12a** (0.313 g, 53%) as a yellow amorphous solid. Spectral data were in accord with that reported above.

### Ethyl 10-Hydroxy-1-oxo-1*H*-benzo[*g*]isochromene-3-carboxylate (**12b**)

Purified using 80% CHCl_3_/hexanes to give **12b** (0.030 g 31%) as a yellow, amorphous solid: ^1^H NMR (400 MHz, CDCl_3_) δ 12.14 (s, 1H), 8.47 (d, *J* = 8.3 Hz, 1H), 7.86 (d, *J* = 8.3 Hz, 1H), 7.75–7.69 (m, 1H), 7.64–7.58 (m, 1H), 7.54 (s, 1H), 7.45 (s, 1H), 4.44 (q, *J* = 7.1 Hz, 2H), 1.43 (t, *J* = 7.2 Hz, 3H); ^13^C{^1^H} NMR (101 MHz, CDCl_3_) δ 166.4, 162.1, 160.4, 141.6, 137. 7, 131.1, 128.8, 128.2, 127.0, 124.6, 124.5, 117.5, 114.5, 100.7, 62.4, 14.4; HRMS (ESI) calcd for C_16_H_13_O_5_ [M + H]^+^ 285.0757, found 285.0749.

### Propyl 10-Hydroxy-1-oxo-1*H*-benzo[*g*]isochromene-3-carboxylate (**12c**)

Purified using a 90–100% CHCl_3_/petroleum ether to give impure **12c** (0.029 g) as an orange, amorphous solid. A portion of this material (6.0 mg) was purified by HPLC (75% B for 15 min, 75–100% B over 2 min, 100% B for 4 min, 100–75% B over 1 min, 75% B for 3 min) to give **12c** (5.0 mg, 22%) as an orange, amorphous solid: ^1^H NMR (400 MHz, CDCl_3_) δ 12.16 (s, 1H), 8.48 (d, *J* = 8.4 Hz, 1H), 7.86 (d, *J* = 8.2 Hz, 1H), 7.74–7.68 (m, 1H), 7.65–7.58 (m, 1H), 7.55 (s, 1H), 7.47 (s, 1H), 4.33 (t, *J* = 6.5 Hz, 2H), 1.83 (sextet, *J* = 7.1 Hz, 2H), 1.05 (t, *J* = 7.4 Hz, 3H); ^13^C{^1^H} NMR (101 MHz, CDCl_3_) δ 166.2, 162.0, 160.3, 141.5, 137.6, 131.0, 128.7, 128.0, 126.9, 124.4, 124.4, 117.3, 114.3, 100.6, 67.8, 22.0, 10.4. HRMS (ESI) calcd for C_17_H_15_O_5_ [M + H]^+^ 299.0914, found 299.0907.

### Butyl 10-Hydroxy-1-oxo-1*H*-benzo[*g*]isochromene-3-carboxylate (**12d**)

Purified using 0–10% acetone in 50% CHCl_3_/petroleum ether to give **12d** (0.046 g, 40%) as a yellow, amorphous solid: ^1^H NMR (400 MHz, CDCl_3_) δ 12.13 (s, 1H), 8.44 (d, *J* = 8.4 Hz, 1H), 7.84 (d, *J* = 8.3 Hz, 1H), 7.73–7.68 (m, 1H), 7.62–7.57 (m, 1H), 7.51 (s, 1H), 7.44 (s, 1H), 4.37 (t, *J* = 6.7 Hz, 2H), 1.78 (p, *J* = 6.8 Hz, 2H), 1.49 (sextet, *J* = 7.3 Hz, 2H), 1.00 (t, *J* = 7.4 Hz, 3H); ^13^C{^1^H} NMR (101 MHz, CDCl_3_) δ 166.4, 162.2, 160.5, 141.7, 137.7, 131.1, 128.8, 128.2, 127.0, 124.6, 124.5, 117.5, 114.5, 100.8, 66.3, 30.7, 19.3, 13.9; HRMS (ESI) calcd for C_18_H_17_O_5_ [M + H]^+^ 313.1071, found 313.1074.

### Isopropyl 10-Hydroxy-1-oxo-1*H*-benzo[*g*]isochromene-3-carboxylate (**12e**)

Purified using 0–10% acetone in 50% CHCl_3_/petroleum ether to give impure **12e** (0.036 g) as a yellow, amorphous solid. A portion of this material (10.0 mg) was purified by HPLC (80% B for 10 min, 80–100% B over 3 min, 100% B for 4 min, 100–80% B over 1 min, 80% B for 3 min) to give **12e** (7.0 mg, 23%) as a yellow, amorphous solid: ^1^H NMR (400 MHz, CDCl_3_) δ 12.16 (s, 1H), 8.47 (d, *J* = 8.4 Hz, 1H), 7.86 (d, *J* = 8.3 Hz, 1H), 7.76–7.68 (m, 1H), 7.63–7.57 (m, 1H), 7.53 (s, 1H), 7.46 (s, 1H), 5.33–5.23 (septet. *J* = 6.2 Hz, 1H), 1.41 (d, *J* = 6.3 Hz, 6H); ^13^C{^1^H} NMR (101 MHz, CDCl_3_) δ 166.5, 162.1, 159.9, 141.9, 137.7, 131.1, 128.9, 128.2, 127.0, 124.6, 124.5, 117.4, 114.3, 100.8, 70.2, 22.0; HRMS (ESI) calcd for C_17_H_15_O_5_ [M + H]^+^ 299.0914, found 299.0919

### 3-Methylbut-2-en-1-yl 10-Hydroxy-1-oxo-1*H*-benzo[*g*]isochromene-3-carboxylate (**12g**)

Purified using 75–100% CHCl_3_/hexanes to give impure **12g** (0.034 g) as an orange, amorphous solid. A portion of this material (7.0 mg) was purified by HPLC (75–100% B over 13 min, 100% B for 4 min, 100–75% B over 1 min, 75% B for 3 min) to give **12g** (5.0 mg, 21%) as an orange, amorphous solid: ^1^H NMR (400 MHz, CDCl_3_) δ 12.16 (s, 1H), 8.49 (d, *J* = 8.5 Hz, 1H), 7.87 (d, *J* = 8.2 Hz, 1H), 7.74–7.70 (m, 1H), 7.64–7.59 (m, 1H), 7.56 (s, 1H), 7.47 (s, 1H), 5.51–5.44 (m, 1H), 4.87 (d, *J* = 7.4 Hz, 2H), 1.81 (s, 3H), 1.79 (s, 3H); ^13^C{^1^H} NMR (126 MHz, CDCl_3_) δ 166.3, 162.0, 160.3, 141.6, 140.5, 137.7, 131.0, 128.7, 128.1, 126.9, 124.5, 124.4, 117.8, 117.4, 116.9, 114.4, 63.1, 25.9, 18.2; HRMS (ESI) calcd for C_19_H_16_O_5_Na [M + Na]^+^ 347.0890, found 347.0884

### 2-(Trimethylsilyl)ethyl 10-Hydroxy-1-oxo-1*H*-benzo[*g*]isochromene-3-carboxylate (**12h**)

Purified using 75–100% CHCl_3_/hexanes to give impure **12h** (0.014 g) as a yellow, amorphous solid. A portion of this material (9.0 mg) was purified by HPLC (75–100% B over 10 min, 100% B for 4 min, 100–90% B over 2 min, 90% B for 4 min) to give **12h** (5.0 mg, 6%) as a yellow, amorphous solid: ^1^H NMR (400 MHz, CDCl_3_) δ 12.17 (s, 1H), 8.48 (d, *J* = 8.6 Hz, 1H), 7.87 (d, *J* = 8.2 Hz, 1H), 7.75–7.69 (m, 1H), 7.64–7.59 (m, 1H), 7.55 (s, 1H), 7.48 (s, 1H), 4.58–4.39 (m, 2H), 1.23–1.14 (m, 2H), 0.11 (s, 9H); ^13^C{^1^H} NMR (126 MHz, CDCl_3_) δ 166.2, 162.0, 160.4, 141.6, 137.5, 130.9, 128.7, 128.0, 126.8, 124.4, 124.3, 117.3, 114.2, 100.1, 64.7, 17.41, −1.5; HRMS (ESI) calcd for C_19_H_20_O_5_SiNa [M + Na]^+^ 379.0972, found 379.0962.

### 3-Chloropropyl 10-Hydroxy-1-oxo-1*H*-benzo[*g*]isochromene-3-carboxylate (**12i**)

Purified using 70–100% CHCl_3_/hexanes to give impure **12i** (0.036 g) as a yellow, amorphous solid. A portion of this material (7.7 mg) was purified by HPLC (70% B for 15 min, 70–100% B over 2 min, 100% B for 4 min, 100–70% B over 1 min, 70% B for 3 min) to give **12i** (1.6 mg, 6%) as a yellow, amorphous solid: ^1^H NMR (400 MHz, CDCl_3_) δ 12.14 (s, 1H), 8.48 (d, *J* = 8.0 Hz, 1H), 7.87 (d, *J* = 8.2 Hz, 1H), 7.75–7.70 (m, 1H), 7.65–7.60 (m, 1H), 7.57 (s, 1H), 7.49 (s, 1H), 4.54 (t, *J* = 6.1 Hz, 2H), 3.73 (t, *J* = 6.3 Hz, 2H), 2.27 (p, *J* = 6.2 Hz, 2H); ^13^C{^1^H} NMR (101 MHz, CDCl_3_) δ 166.2, 162.1, 160.2, 141.2, 137.5, 131.1, 128.5, 128.1, 127.0, 124.5, 124.4, 117.5, 114.8, 100.58, 62.9, 41.0, 31.4; HRMS (ESI) calcd for C_17_H_14_ClO_5_ [M + H]^+^ 333.0524, found 333.0515.

### 3-Bromopropyl 10-Hydroxy-1-oxo-1*H*-benzo[*g*]isochromene-3-carboxylate (**12j**)

Purified using 60–100% CHCl_3_/hexane to give impure **12j** (0.020 g) as a yellow, amorphous solid. A portion of this material (9.0 mg) was purified by HPLC (70% B for 15 min, 70–100% B over 2 min, 100% B for 4 min, 100–70% B over 1 min, 70% B for 3 min) to give **12j** (3.0 mg, 5%) as a yellow, amorphous solid: ^1^H NMR (500 MHz, CDCl_3_) δ 12.14 (s, 1H), 8.50 (d, *J* = 8.4 Hz, 1H), 7.88 (d, *J* = 8.3 Hz, 1H), 7.76–7.70 (m, 1H), 7.66–7.60 (m, 1H), 7.58 (s, 1H), 7.50 (s, 1H), 4.52 (t, *J* = 6.1 Hz, 2H), 3.57 (t, *J* = 6.4 Hz, 2H), 2.36 (p, *J* = 6.2 Hz, 2H); ^13^C{^1^H} NMR (126 MHz, CDCl_3_) δ 166.3, 162.2, 160.3, 141.3, 137.7, 131.2, 128.7, 128.2, 127.2, 124.7, 124.6, 117.7, 115.0, 100.7, 64.0, 31.7, 29.3; HRMS (ESI) calcd for C_17_H_14_BrO_5_ [M + H]^+^ 377.0019, found 377.0010.

### Benzyl 10-Hydroxy-1-oxo-1*H*-benzo[*g*]isochromene-3-carboxylate (**12o**)

Purified using 75–100% CHCl_3_/hexanes to give impure **12o** (0.046 g) as a yellow, amorphous solid. A portion of this material (6.0 mg) was purified by HPLC (90–100% B over 13 min, 100% B for 4 min, 100–90% B over 1 min, 90% B for 3 min) to give **12o** (5.0 mg, 30%) as a yellow, amorphous solid: ^1^H NMR (400 MHz, CDCl_3_) δ 12.14 (s, 1H), 8.48 (d, *J* = 8.4 Hz, 1H), 7.85 (d, *J* = 8.2 Hz, 1H), 7.74–7.68 (m, 1H), 7.64–7.55 (m, 2H), 7.50–7.34 (m, 6H), 5.40 (s, 2H); ^13^C{^1^H} NMR (101 MHz, CDCl_3_) δ 166.1, 162.0, 160.1, 141.2, 137.5, 134.9, 131.0, 128.7, 128.7, 128.6, 128.5, 128.0, 126.9, 124.4, 124.3, 117.4, 114.8, 100.6, 67.7; HRMS (ESI) calcd for C_21_H_15_O_5_ [M + H]^+^ 347.0914, found 347.0904.

### 4-Methoxybenzyl 10-Hydroxy-1-oxo-1*H*-benzo[*g*]isochromene-3-carboxylate (**12p**)

Purified using 75–100% CHCl_3_/hexane to give impure **12p** (0.026 g) as a yellow, amorphous solid. A portion of this material (5.0 mg) was purified by HPLC (75–100% B over 13 min, 100% B for 4 min, 100–75% B over 1 min, 75% B for 3 min) to give **12p** (2.0 mg, 8%) as a yellow, amorphous solid: ^1^H NMR (400 MHz, CDCl_3_) δ 12.15 (s, 1H), 8.50–8.46 (m, 1H), 7.85 (d, *J* = 8.2 Hz, 1H), 7.72 (ddd, *J* = 8.2, 6.8, 1.3 Hz, 1H), 7.61 (ddd, *J* = 8.2, 6.8, 1.2 Hz, 1H), 7.55 (d, *J* = 0.5 Hz, 1H), 7.46 (s, 1H), 7.44–7.37 (m, 2H), 6.90–6.96 (m, 2H), 5.34 (s, 2H), 3.83 (s, 3H); ^13^C{^1^H} NMR (126 MHz, CDCl_3_) δ 166.3, 162.2, 160.3, 160.1, 141.5, 137.7, 131.1, 130.8, 128.8, 128.2, 127.3, 127.1, 124.6, 124.5, 117.6, 114.8, 114.3, 100.8, 67.8, 55.5; HRMS (ESI) calcd for C_22_H_16_O_6_Na [M + Na]^+^ 399.0839, found 399.0831.

### 10-Hydroxy-*N*,*N*-diisopropyl-1-oxo-1*H*-benzo[*g*]isochromene-3-carboxamide (**12s**)

Purified using 0–20% EtOAc/CHCl_3_ to give **12s** (0.054 g, 44%) as an orange, amorphous solid: ^1^H NMR (400 MHz, CDCl_3_) δ 12.07 (s, 1H), 8.42 (d, *J* = 8.4 Hz, 1H), 7.80 (d, *J* = 8.3 Hz, 1H), 7.70–7.63 (m, 1H), 7.54 (dt, *J* = 8.2, 6.8 Hz, 1H), 7.30 (s, 1H), 6.88 (s, 1H), 3.96 (br s, 1H), 3.63 (br s, 1H), 1.41 (br s, 12H); ^13^C{^1^H} NMR (101 MHz, CDCl_3_) δ 166.4, 162.0, 161.9, 148.3, 138.0, 130.9, 129.8, 127.8, 126.2, 124.3, 123.7, 115.5, 108.6, 100.6, 50.8 (br), 46.9 (br), 20.9 (br); HRMS (ESI) calcd for C_20_H_22_NO_4_ [M + H]^+^ 330.1543, found 330.1536.

### 10-Hydroxy-3-(piperidine-1-carbonyl)-1*H*-benzo[*g*]isochromen-1-one (**12t**)

Purified using 5–30% acetone in 66% CHCl_3_/petroleum ether to give **12t** (0.076 g, 64%) as an amorphous, orange solid: ^1^H NMR (400 MHz, CDCl_3_) δ 12.08 (s, 1H), 8.46 (d, *J* = 8.4 Hz, 1H), 7.83 (d, *J* = 8.3 Hz, 1H), 7.70 (ddd, *J* = 8.3, 6.9, 1.3 Hz, 1H), 7.58 (ddd, *J* = 8.3, 6.9, 1.2 Hz, 1H), 7.37 (s, 1H), 7.04 (s, 1H), 3.63 (br s, 4H), 1.71 (br s, 6H); ^13^C{^1^H} NMR (100 MHz, CDCl_3_) δ 166.3, 162.1, 161.3, 146.8, 138.0, 131.1, 129.6, 128.0, 126.5, 124.4, 123.9, 116.0, 110.7, 100.6, 48.5 (br), 44.3 (br), 26.6 (br), 25.7 (br), 24.6; HRMS (ESI) calcd for C_19_H_18_NO_4_ [M + H]^+^ 324.1230, found 324.1226.

### 10-Hydroxy-3-(morpholine-4-carbonyl)-1*H*-benzo[*g*]isochromen-1-one (**12u**)

Purified using 10–35% acetone in 66% CHCl_3_/petroleum ether to give **12u** (0.064 g, 67%) as an orange amorphous solid: ^1^H NMR (400 MHz, CDCl_3_) δ 12.03 (s, 1H), 8.47 (*J* = 8.5 Hz, 1H), 7.88–7.82 (m, 1H), 7.74–7.68 (m, 1H), 7.63–7.56 (m, 1H), 7.40 (s, 1H), 7.17 (s, 1H), 3.78 (br s, 8H); ^13^C{^1^H} NMR (101 MHz, CDCl_3_) δ 166.1, 162.2, 161.3, 145.9, 138.0, 131.2, 129.2, 128.0, 126.7, 124.5, 124.1, 116.5, 112.2, 100.4, 67.0 (br), 53.6 (br), 47.5 (br); HRMS (ESI) calcd for C_18_H_16_NO_5_ [M + H]^+^ 326.1023, found 326.1027.

### *tert*-Butyl Hexyl(10-hydroxy-1-oxo-1*H*-benzo[*g*]isochromene-3-carbonyl)carbamate (12v*)

Purified using pure CHCl_3_ followed by another purification using 75–100% CHCl_3_/hexanes to give 12v* (0.046 g, 35%) as a yellow amorphous solid: ^1^H NMR (400 MHz, CDCl_3_) δ 12.12 (s, 1H), 8.46 (d, *J* = 8.4 Hz, 1H), 7.84 (d, *J* = 8.2 Hz, 1H), 7.69 (ddd, *J* = 8.6, 6.8, 1.3 Hz, 1H), 7.58 (dt, *J* = 8.2, 6.9, 1.2 Hz, 1H), 7.41 (s, 1H), 7.18 (s, 1H), 3.78–3.71 (t, *J* = 7.7 Hz, 2H), 1.73–1.62 (m, 2H), 1.42 (s, 9H), 1.38–1.31 (m, 6H), 0.89 (t, *J* = 6.9 Hz, 3H); ^13^C{^1^H} NMR (101 MHz, CDCl_3_) δ 166.0, 164.8, 162.1, 152.6, 146.9, 137.9, 131.1, 129.4, 128.0, 126.7, 124.4, 124.2, 116.8, 111.0, 100.6, 84.0, 46.2, 31.6, 28.8, 27.9, 26.6, 22.7, 14.1; HRMS (ESI) calcd for C_25_H_28_NO_6_ [M – H]^−^ 438.1922, found 438.1909.

### Dimethyl (2*Z*,4*Z*)-2-Hydroxyhexa-2,4-dienedioate (**13**)

Isolated as an amorphous orange solid (0.008 g, 5%) from isolation optimization trials: ^1^H NMR (400 MHz, CDCl_3_) δ 7.71 (dd, *J* = 15.6, 11.7 Hz, 1H), 6.35 (br s, 1H), 6.26 (dd, *J* = 11.7, 0.8 Hz, 1H), 6.03 (dd, *J* = 15.6, 0.9 Hz, 1H), 3.90 (s, 3H), 3.76 (s, 3H); ^13^C{^1^H} NMR (101 MHz, CDCl_3_) δ 167.1, 165.3, 143.9, 137.1, 122.9, 108.8, 53.6, 51.8; HRMS (ESI) calcd for C_8_H_10_O_5_Na [M + Na]^+^ 209.0420, found 209.0434.

### 2-((Phenylsulfinyl)methyl)benzoic Acid (**15**)

A flask was charged with **6** (0.295 g, 1.13 mmol), water (25 mL) and methanol (25 mL). Solid NaOH (0.290 g, 7.13 mmol) was added and the mixture was heated to 50 °C (oil bath) for 4 h. After cooling, the methanol was removed under reduced pressure. Unreacted starting material was removed by washing the aqueous solution with CH_2_Cl_2_ (2 × 25 mL). The aqueous layer was acidified (pH ∼ 2) using 3 M HCl and **15** (0.333 g, 68%) was collected via filtration as a white precipitate: ^1^H NMR (400 MHz, CD_3_OD) δ 8.12–8.05 (m, 1H), 7.63–7.50 (m, 5H), 7.48–7.43 (m, 2H), 7.14–7.09 (m, 1H), 4.84 (d, *J* = 12.0 Hz, 1H), 4.47 (d, *J* = 12.0 Hz, 1H); ^13^C{^1^H} NMR (101 MHz, CD_3_OD) δ 169.8, 144.0, 134.4, 133.4, 133.2, 132.67, 132.65, 131.4, 130.3, 129.8, 125.5, 64.0; HRMS (ESI) calcd for C_14_H_13_O_3_S [M + H]^+^ 261.0585, found 261.0577

### Phenyl 2-((Phenylsulfinyl)methyl)benzoate (**16**)

To a stirring solution of **15** (0.295 g, 1.13 mmol), phenol (0.110 g, 1.17 mmol), and EDC·HCl (0.240 g, 1.25 mmol) dissolved in CH_2_Cl_2_ (6 mL) was added DMAP (14.0 mg, 0.115 mmol). After stirring at rt for 24 h, the reaction was quenched by the addition of NaHCO_3_ (3 mL), the layers were separated, and the aqueous layer was extracted with CH_2_Cl_2_ (2 × 3 mL). The combined organic layers were washed with NaOH (0.5 M, 8 mL) and brine (8 mL), dried (Na_2_SO_4_), and concentrated. The residue purified was using flash chromatography (SiO_2_, 33–50% EtOAc/hexanes) to give **16** (0.233 g, 61%) as a clear and colorless oil: ^1^H NMR (400 MHz, CDCl_3_) δ 8.38–8.17 (m, 1H), 7.59–7.41 (m, 9H), 7.33–7.28 (m, 1H), 7.25–7.21 (m, 3H), 4.92 (d, *J* = 12.1 Hz, 1H), 4.26 (d, *J* = 12.2 Hz, 1H); ^13^C{^1^H} NMR (101 MHz, CDCl_3_) δ 165.5, 150.8, 143.8, 133.6, 133.11, 133.10, 131.8, 131.0, 129.7, 129.0, 128.9, 128.7, 126.2, 124.3, 121.9, 63.0; HRMS (ESI) calcd for C_20_H_16_O_3_SNa [M + Na]^+^ 359.0718, found 359.0710.

### Methyl 2-Methoxy-6-((phenylsulfinyl)methyl)benzoate (**18**)

The following reaction was carried out in an analogous manner to the published procedure.^22^**Caution!** Carbon tetrachloride is highly toxic and should be handled exclusively in a fume cabinet to avoid vapor exposure. Methyl methoxytoluate **17** (0.195 g, 1.08 mmol), *N*-bromosuccinimide (NBS) (0.214 g, 1.20 mmol), and AIBN (9.6 mg, 0.0061 mmol) were added to a dried flask. After purging with nitrogen, CCl_4_ (12 mL) was added, and the reaction was refluxed for 3.5 h. After cooling to rt, the mixture was filtered and concentrated to give **17-Br** which was carried onto the next step without further purification.

Dried K_2_CO_3_ (0.299 g, 2.16 mmol) was added to a flame-dried flask, followed by **17-Br** and thiophenol (0.124 mL, 1.21 mmol). After purging with nitrogen gas, acetone (20 mL) was added and the mixture was heated to reflux (oil bath) overnight. After cooling to room temperature, Et_2_O (25 mL) and 5% aqueous NaOH solution (5 mL) were added and the layers were separated. The aqueous layer was concentrated and extracted with EtOAc (2 × 10 mL). The combined organic layers were dried (Na_2_SO_4_) and concentrated furnishing **17-S** which was used without further purification.

Thiol **17-S** was dissolved in methanol (25 mL) and water (5 mL). NaIO_4_ (0.230 g, 1.08 mmol) was added and the reaction was stirred overnight, after which the methanol was removed under reduced pressure. The mixture was extracted with EtOAc (3 × 20 mL), and the combined organic layers were dried (Na_2_SO_4_) and concentrated. The residue was purified using flash chromatography (SiO_2_, 0–65% EtOAc/hexanes) to yield **18** (0.176 g, 53%) as a viscous, off-white oil: ^1^H NMR (400 MHz, CDCl_3_) δ 7.51–7.40 (m, 5H), 7.24 (t, *J* = 8 Hz, 1H), 6.89 (d, *J* = 8.5 1H), 6.64 (d, *J* = 8.0 1H), 4.17 (d, *J* = 12.7 Hz, 1H), 4.03 (d, *J* = 12.7 Hz, 1H), 3.88 (s, 3H), 3.82 (s, 3H); ^13^C{^1^H} NMR (101 MHz, CDCl_3_) δ 167.9, 157.4, 143.5, 131.4, 131.1, 129.8, 129.1, 124.3, 123.8, 123.7, 111.6, 62.5, 56.3, 52.5; HRMS (ESI) calcd for C_16_H_16_O_4_SNa [M + Na]^+^ 327.0667, found 327.0660.

### Methyl 10-Hydroxy-9-methoxy-1-oxo-1*H*-benzo[*g*]isochromene-3-carboxylate (**19**)

The material was prepared according to the general procedure for pyrone annulation using **11a** (0.098 g, 0.636 mmol), **18** (0.079 g, 0.260 mmol), and LiHMDS (0.78 mL, 1 M, 0.78 mmol) in THF (5 mL). Purified using CHCl_3_ followed by 25–40% EtOAc/hexanes to yield **19** (0.015 g, 22%) as a yellow amorphous solid: ^1^H NMR (400 MHz, CDCl_3_) δ 12.93 (s, 1H), 7.60 (t, *J* = 8.1 Hz, 1H), 7.50 (d, *J* = 0.5 Hz, 1H), 7.41 (d, *J* = 8.1 Hz, 1H), 7.37 (s, 1H), 6.95 (d, *J* = 7.9 Hz, 1H), 4.06 (s, 3H), 3.97 (s, 3H); ^13^C{^1^H} NMR (151 MHz, CDCl_3_) δ 166.1, 164.3, 160.8, 159.3, 141.5, 140.3, 131.7, 129.4, 120.8, 117.4, 115.9, 114.2, 107.0, 100.9, 56.4, 53.0; HRMS (ESI) calcd for C_16_H_13_O_6_ [M + H]^+^ 301.0712, found 301.0703.

### Methyl 3-((Phenylsulfinyl)methyl)-2-naphthoate (**22**)

The following reaction was carried out in an analogous manner to the published procedure.^22^**Caution!** Carbon tetrachloride is highly toxic and should be handled exclusively in a fume cabinet to avoid vapor exposure. Naphthoate **21** (0.518 g, 2.59 mmol) was dissolved in CCl_4_ (16 mL). To the stirring mixture was added NBS (0.484 g, 2.73 mmol) and benzoyl peroxide (10.0 mg, 0.0412 mmol). The mixture was heated to reflux (oil bath) and stirred for 6 h, after which the reaction was cooled, filtered, and concentrated to yield **21-Br**, which was used without further purification.

Bromide **21-Br** was dissolved in CHCl_3_ (20 mL) and thiophenol (0.277 mL, 2.72 mmol) and Et_3_N (0.38 mL, 2.7 mmol) were added. The mixture was stirred at rt overnight, after which solids were filtered away. The filtrate was washed with 1 M NaOH (3 × 10 mL), brine (10 mL), and dried (Na_2_SO_4_). Concentration yielded **21-S**, which was used without further purification.

Thioether **21-S** was dissolved in methanol (15 mL) and water (1.5 mL) and after the addition of NaIO_4_ (0.581 g, 2.72 mmol) was stirred at rt overnight. The mixture was concentrated to remove methanol, diluted with EtOAc (10 mL), and filtered. The filtrate was partitioned with water (20 mL) and the aqueous layer was extracted with EtOAc (2 × 20 mL). The combined organic layers were dried (Na_2_SO_4_) and concentrated and the residue was purified using flash chromatography (SiO_2_, 0–33% EtOAc/CHCl_3_) to yield **22** (0.454 g 54%) as an off-white amorphous solid: ^1^H NMR (400 MHz, CDCl_3_) δ 8.61 (s, 1H), 7.91 (d, *J* = 8.0 Hz, 1H), 7.79 (d, *J* = 7.4 Hz, 1H), 7.66–7.52 (m, 5H), 7.50–7.42 (m, 3H), 4.98 (d, *J* = 12.2 Hz, 1H), 4.39 (d, *J* = 12.2 Hz, 1H), 3.96 (s, 3H); ^13^C{^1^H} NMR (101 MHz, CDCl_3_) δ 167.6, 144.2, 134.8, 133.2, 133.0, 132.3, 131.0, 129.1, 129.0, 128.97, 127.9, 127.7, 127.4, 126.6, 124.4, 64.0, 52.4; HRMS (ESI) calcd for C_19_H_17_O_3_S [M + H]^+^ 325.0898, found 325.0897.

### Methyl 12-Hydroxy-1-oxo-1*H*-naphtho[2,3-*g*]isochromene-3-carboxylate (**23**)

The material was prepared according to the general procedure for pyrone annulation using **11a** (0.132 g, 0.856 mmol), **22** (0.119 g, 0.367 mmol), and LiHMDS (1.15 mL, 1.15 mmol) in THF (16 mL). Purified using CHCl_3_ to give **23** (0.018 g, 15%) as an orange amorphous solid: ^1^H NMR (600 MHz, CDCl_3_) δ 12.53 (s, 1H), 9.12 (s, 1H), 8.43 (s, 1H), 8.13 (d, *J* = 8.3 Hz, 1H), 8.03 (d, *J* = 8.4 Hz, 1H), 7.67–7.53 (m, 4H), 3.98 (s, 3H). ^13^C{^1^H} NMR (151 MHz, CDCl_3_) δ 166.5, 163.9, 161.0, 140.8, 134.6, 133.6, 131.8, 129.4, 128.2, 128.0, 127.0, 126.9, 126.7, 125.2, 123.0, 117.9, 115.1, 98.6, 53.0; HRMS (ESI) calcd for C_19_H_13_O_5_ [M + H]^+^ 321.0763, found 321.0752.

## Data Availability

The data underlying this study are available in the published article and its Supporting Information.

## Supporting Information Available

^1^H and ^13^C{^1^H} NMR spectra (PDF)
