# Supplementary material for: Regioselective Annulation of 6-Carboxy-Substituted Pyrones as a Two-Carbon Unit in Formal [4 + 2] Cycloaddition Reactions
Source: J Org Chem. 2024 Jun 13;89(13):9557–68. doi: 10.1021/acs.joc.4c01044 (PMC11232011; doi:10.1021/acs.joc.4c01044)

Supporting Information

Regioselective annulation of 6-carboxy substituted pyrones as a two-carbon unit in formal [4+2] cycloaddition reactions

Zachary A. Kohanov,[a,b] Suzzudul Islam Shuvo,[a,b] and Andrew N. Lowell[a,b,c,]*

[a]Department of Chemistry, Virginia Polytechnic Institute and State University (Virginia Tech), Blacksburg, VA 24061, USA

[b]Center for Emerging, Zoonotic, and Arthropod-borne Pathogens, Virginia Polytechnic Institute and State University (Virginia Tech), Blacksburg, VA 24061, United States

[c]Faculty of Health Sciences, Virginia Polytechnic Institute and State University (Virginia Tech), Blacksburg, VA 24061, United States

alowell@vt.edu

Table of Contents

Title Page S1

Table of Contents S2

^{1}H and $^{13}C\{^{1}H\}$ NMR Spectra S3-S48

Figure S1. ^{1}H NMR of **11a** (400 MHz, $CDCl_3$).

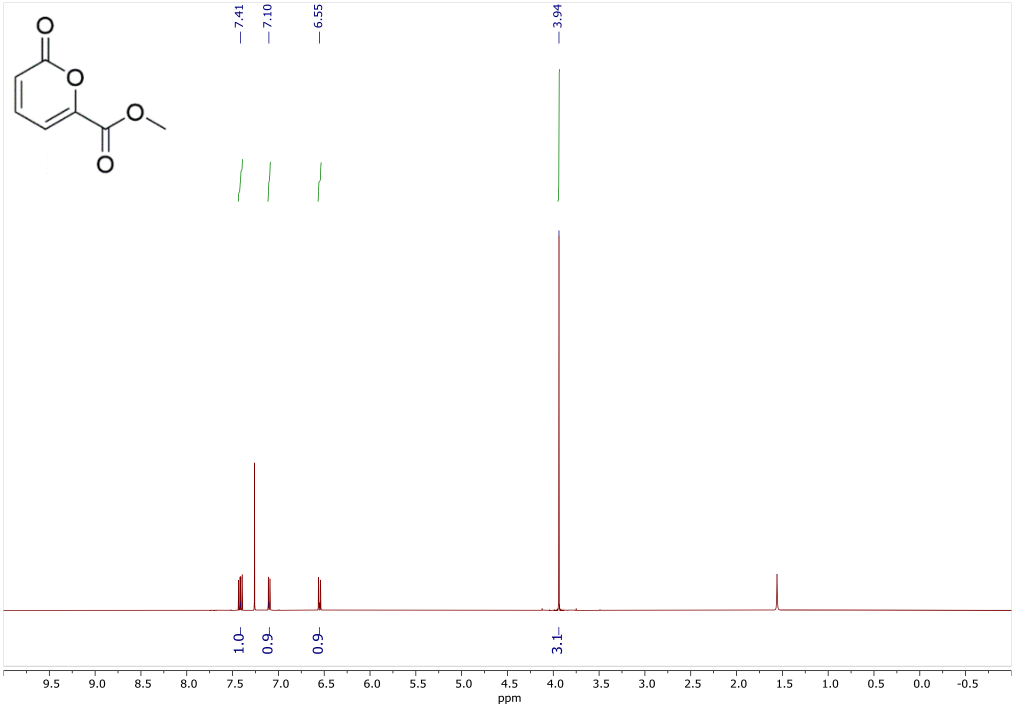

Figure S2. ^{13}C{^{1}H} NMR of **11a** (101 MHz, $CDCl_3$).

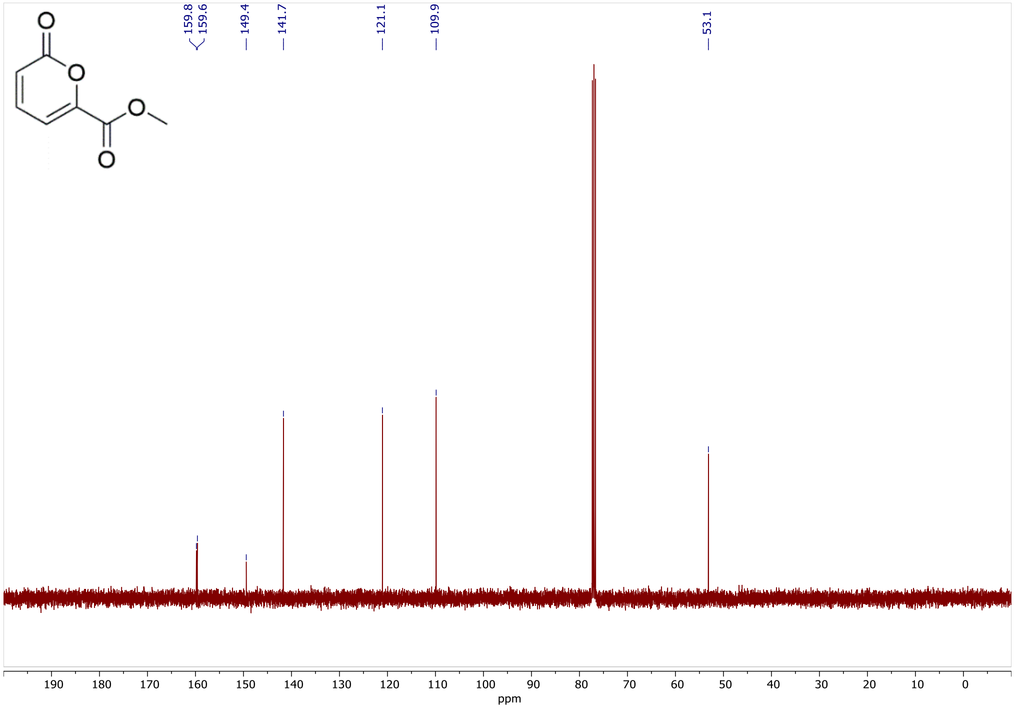

Figure S3. ^{1}H NMR of **11b** (400 MHz, $CDCl_3$).

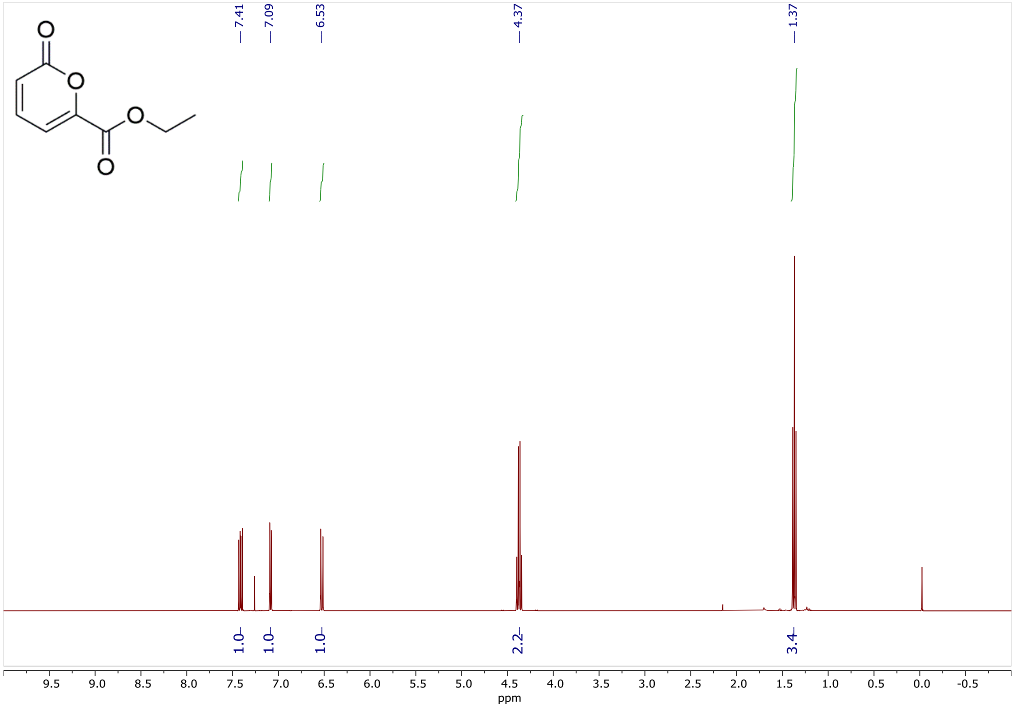

Figure S4. ^{13}C{^{1}H} NMR of **11b** (101 MHz, $CDCl_3$).

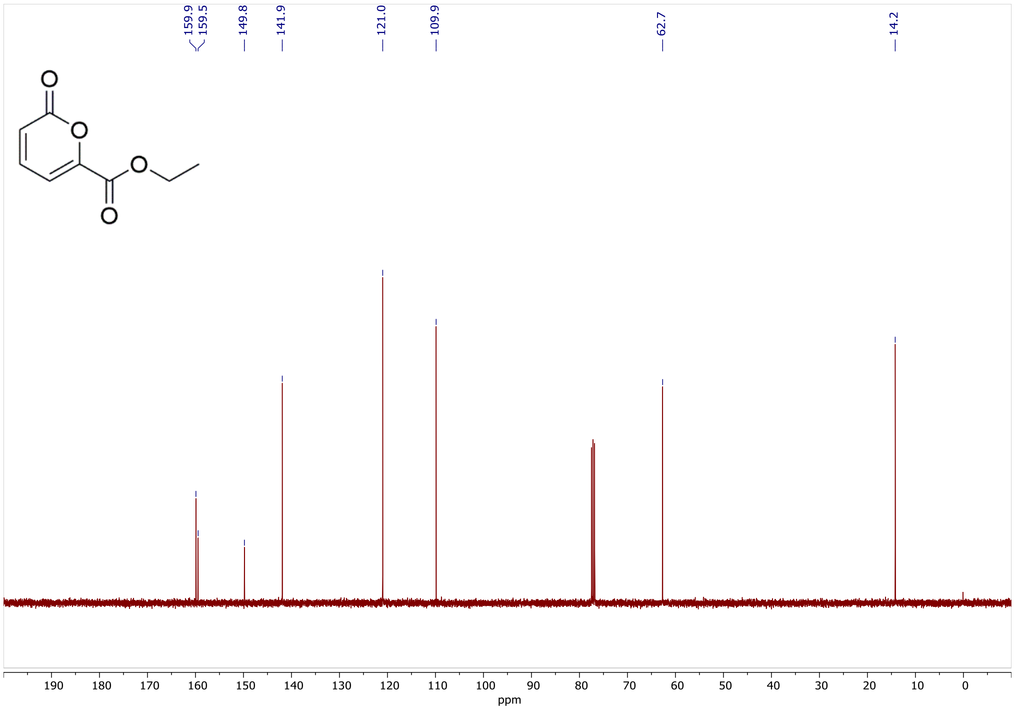

Figure S5. ^{1}H NMR of **11c** plus CH_2Cl_2 and trace EtOAc (400 MHz, $CDCl_3$).

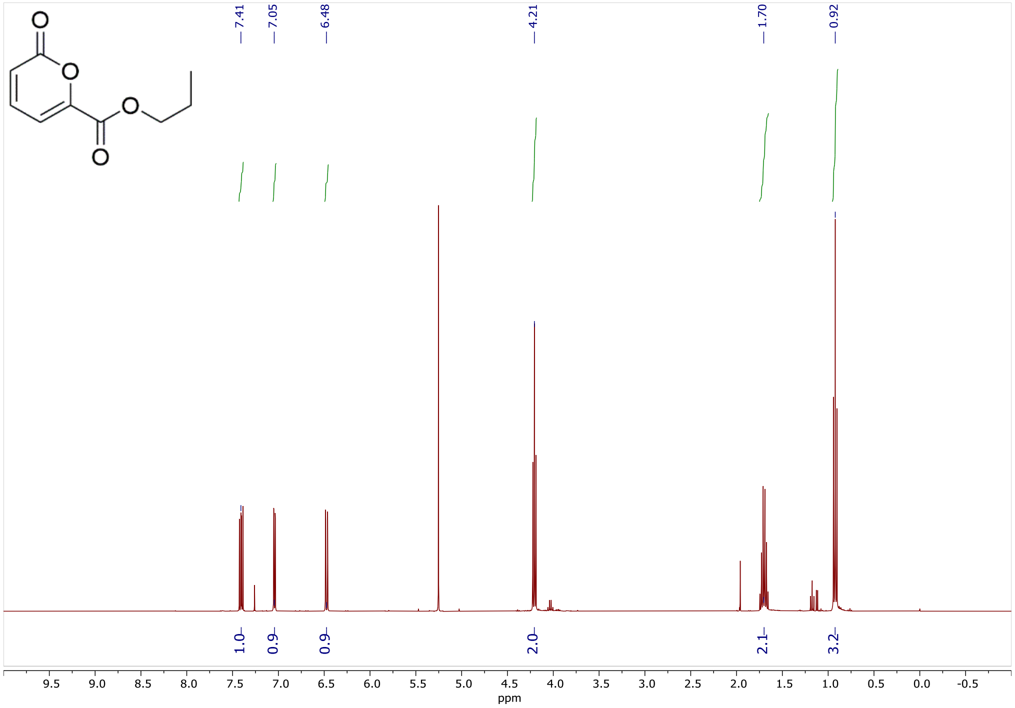

Figure S6. $^{13}C\{^1H\}$ NMR of **11c** (101 MHz, $CDCl_3$).

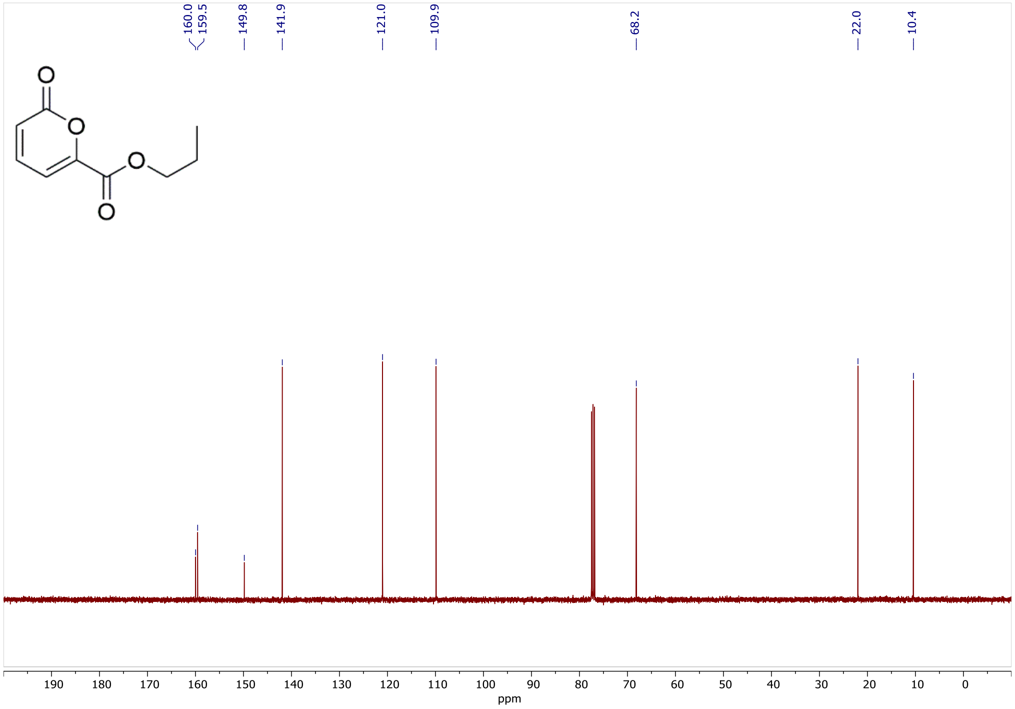

Figure S7. ^{1}H NMR of **11d** (400 MHz, $CDCl_3$).

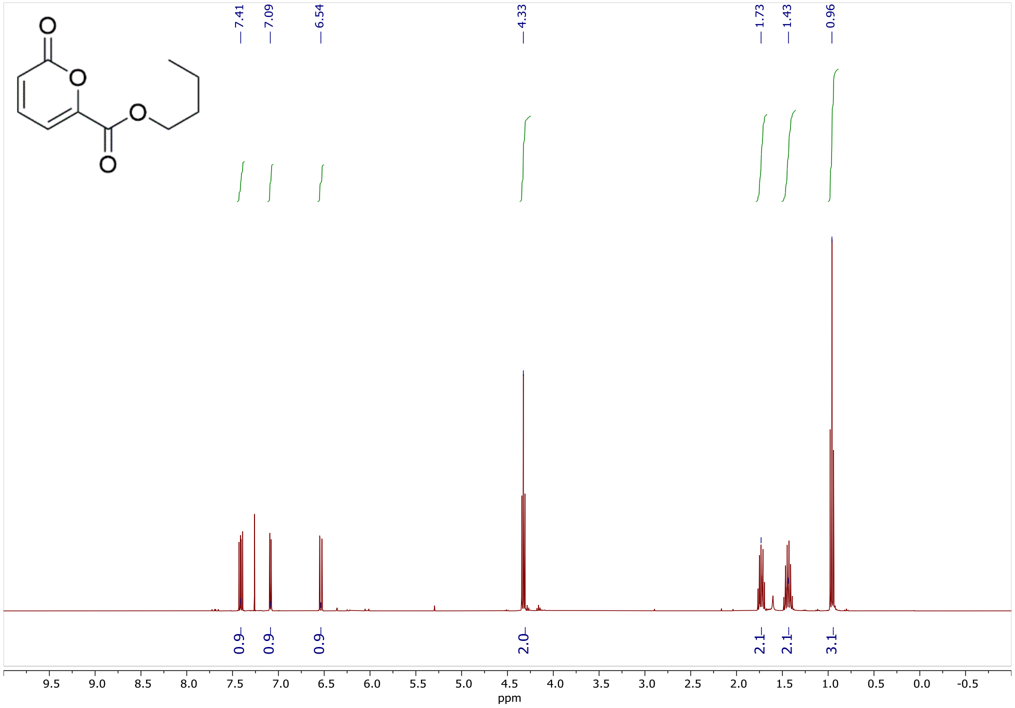

Figure S8. $^{13}C\{^{1}H\}$ NMR of **11d** (101 MHz, $CDCl_3$).

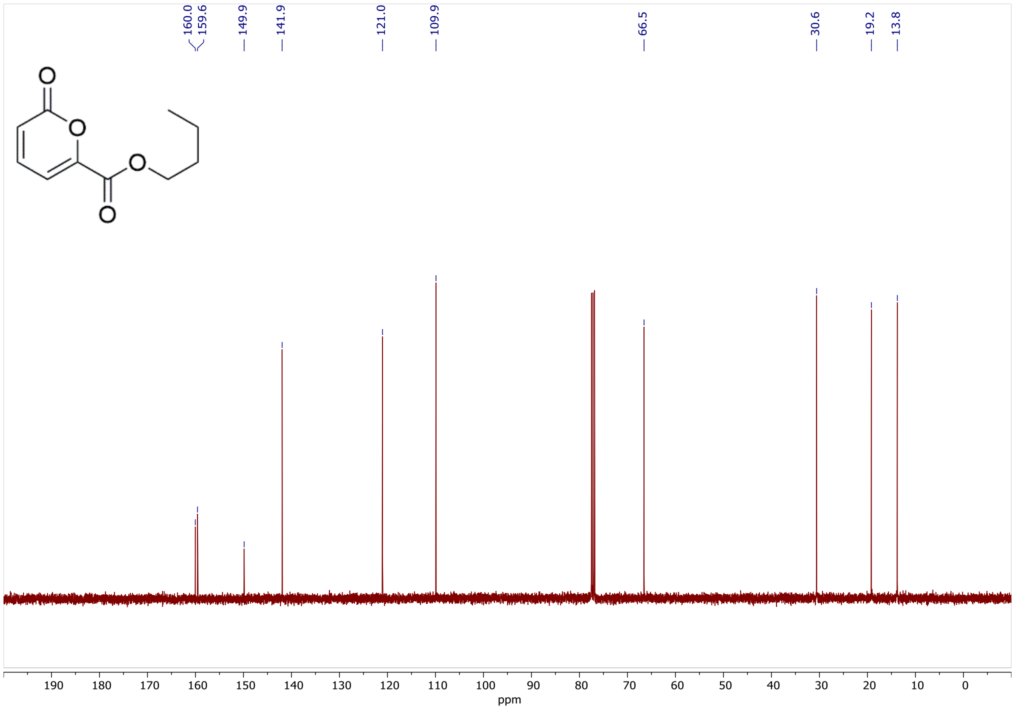

Figure S9. ^{1}H NMR of **11e** (400 MHz, $CDCl_3$).

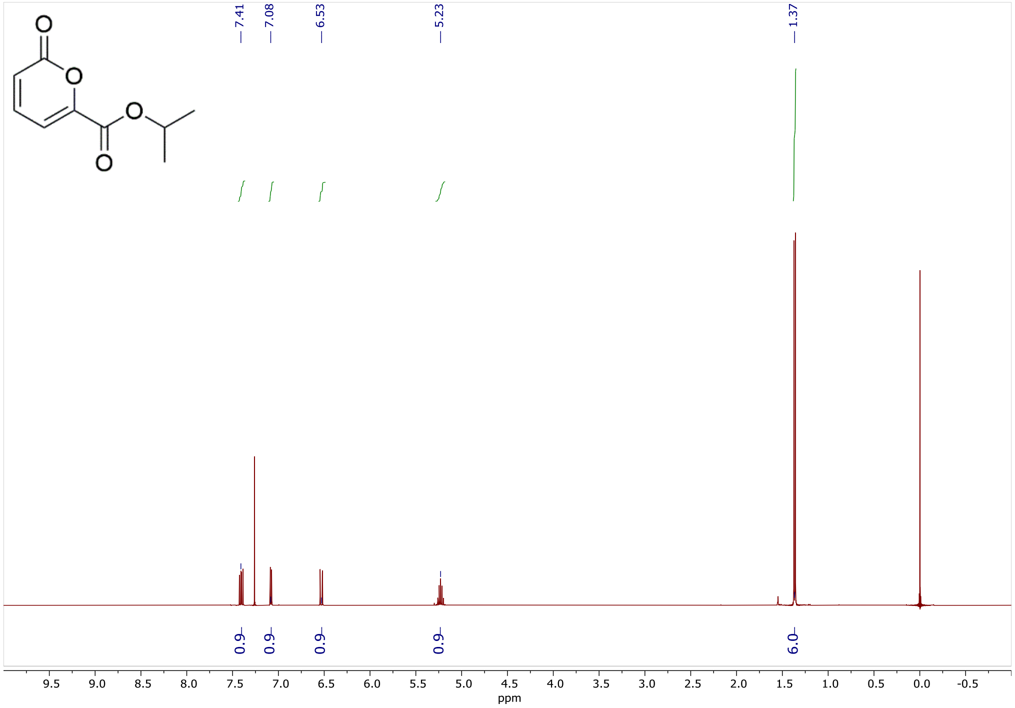

Figure S10. $^{13}C\{^1H\}$ NMR of **11e** (126 MHz, $CDCl_3$).

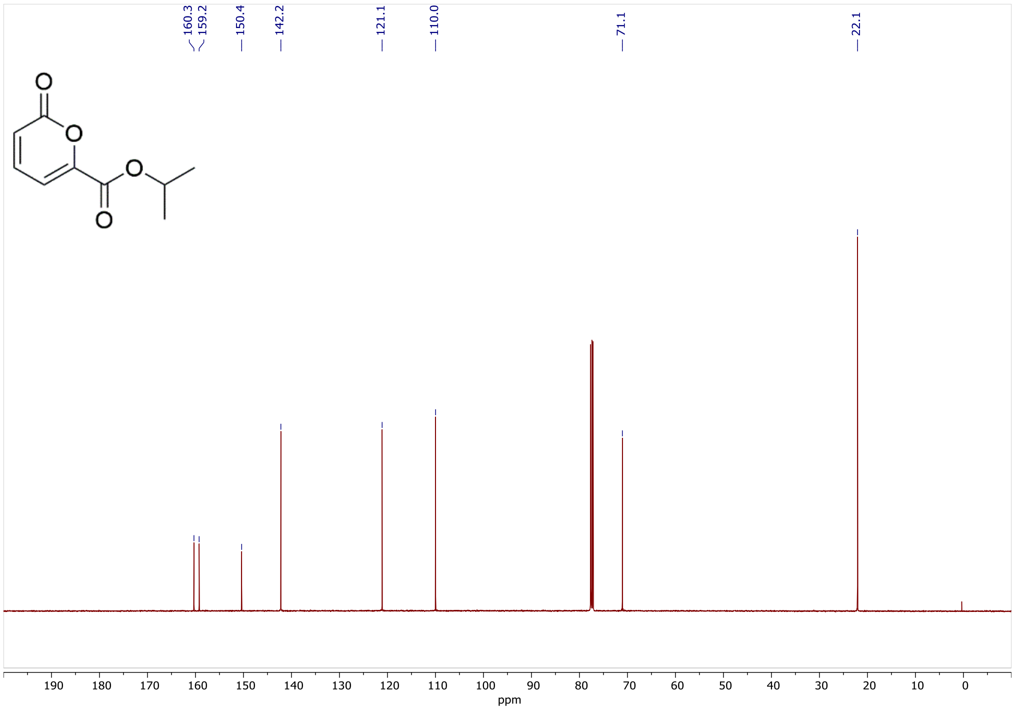

Figure S11. ^{1}H NMR of **11f** (400 MHz, $CDCl_3$).

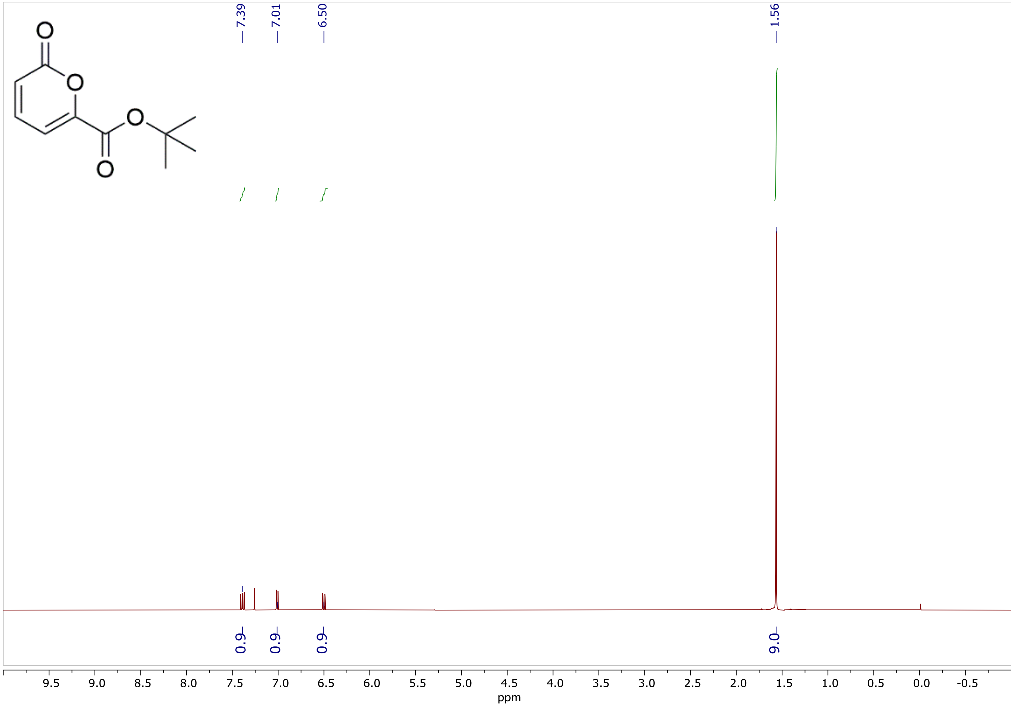

Figure S12. $^{13}C\{^{1}H\}$ NMR of **11f** (101 MHz, $CDCl_3$).

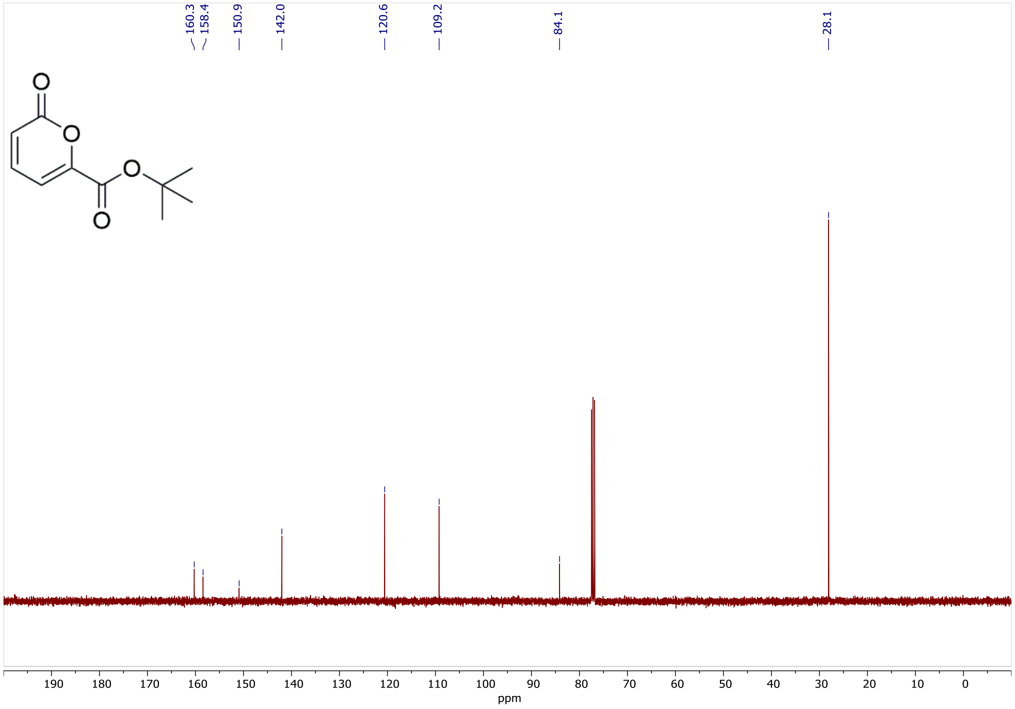

Figure S13. ^{1}H NMR of **11g** (400 MHz, $CDCl_3$).

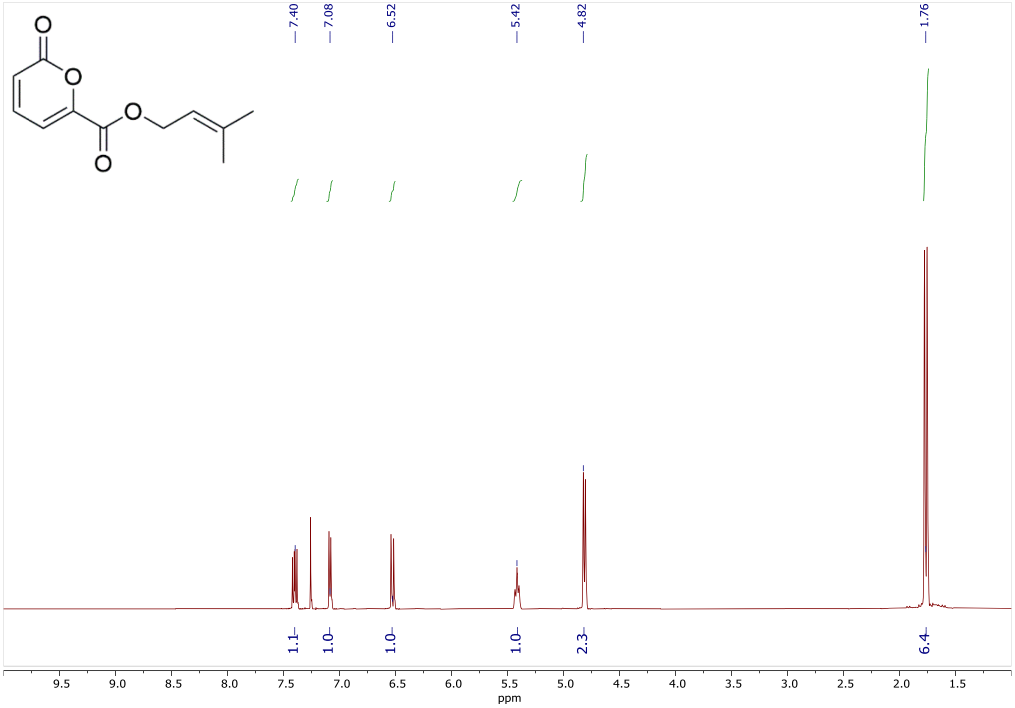

Figure S14. ^{13}C{^{1}H} NMR of **11g** (101 MHz, $CDCl_3$).

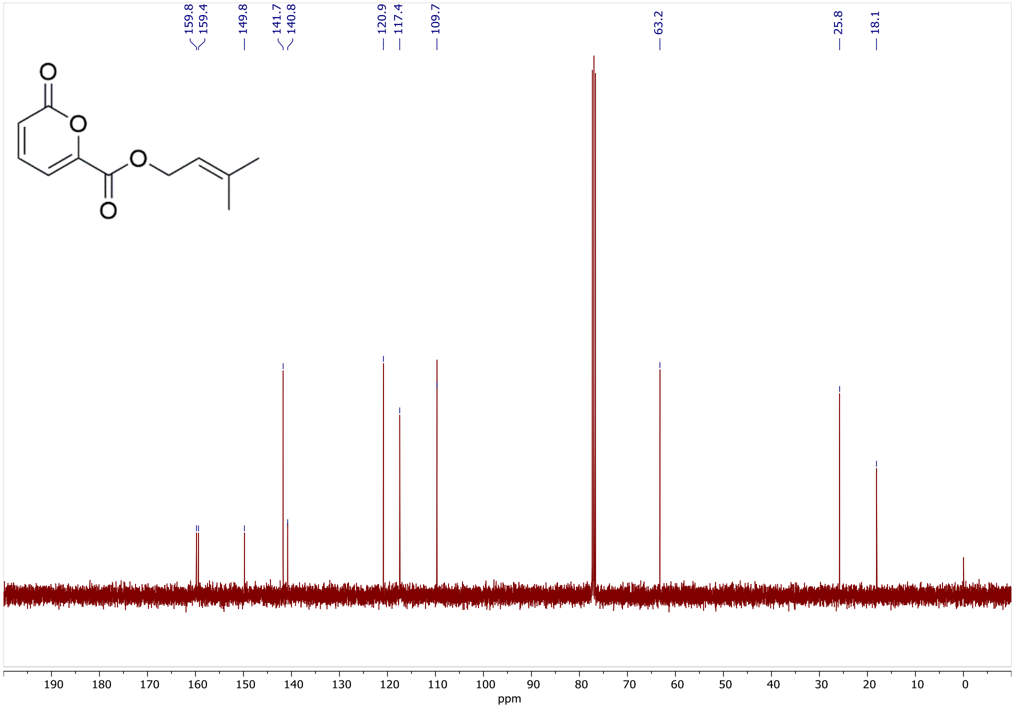

Figure S15. ^{1}H NMR of **11h** (400 MHz, $CDCl_3$).

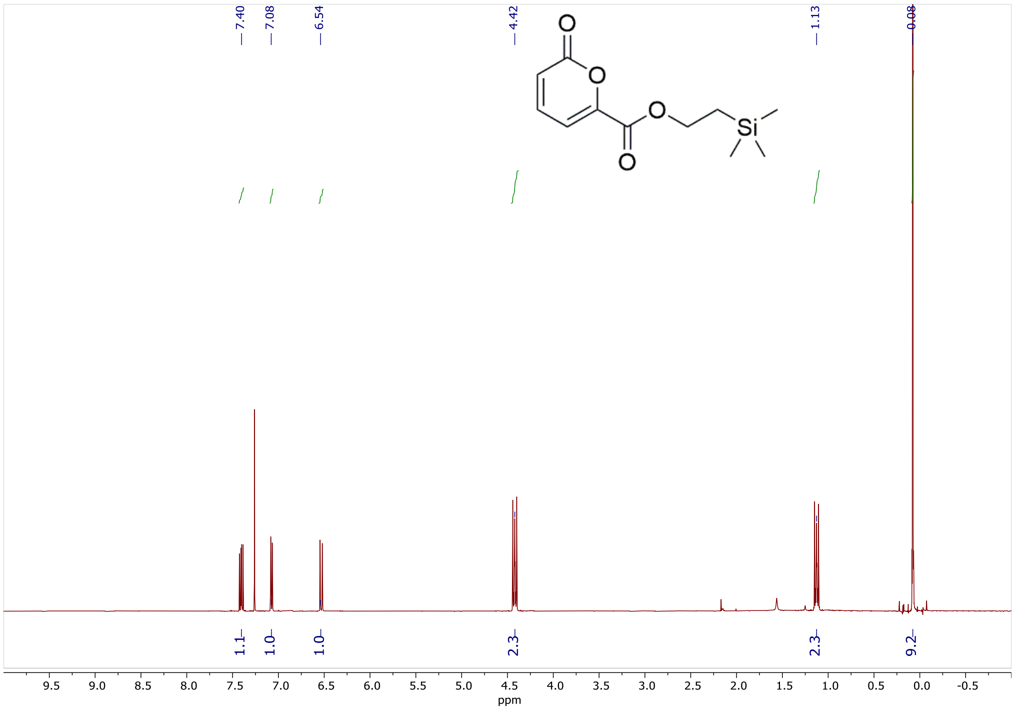

Figure S16. ^{13}C{^{1}H} NMR of **11h** (101 MHz, $CDCl_3$).

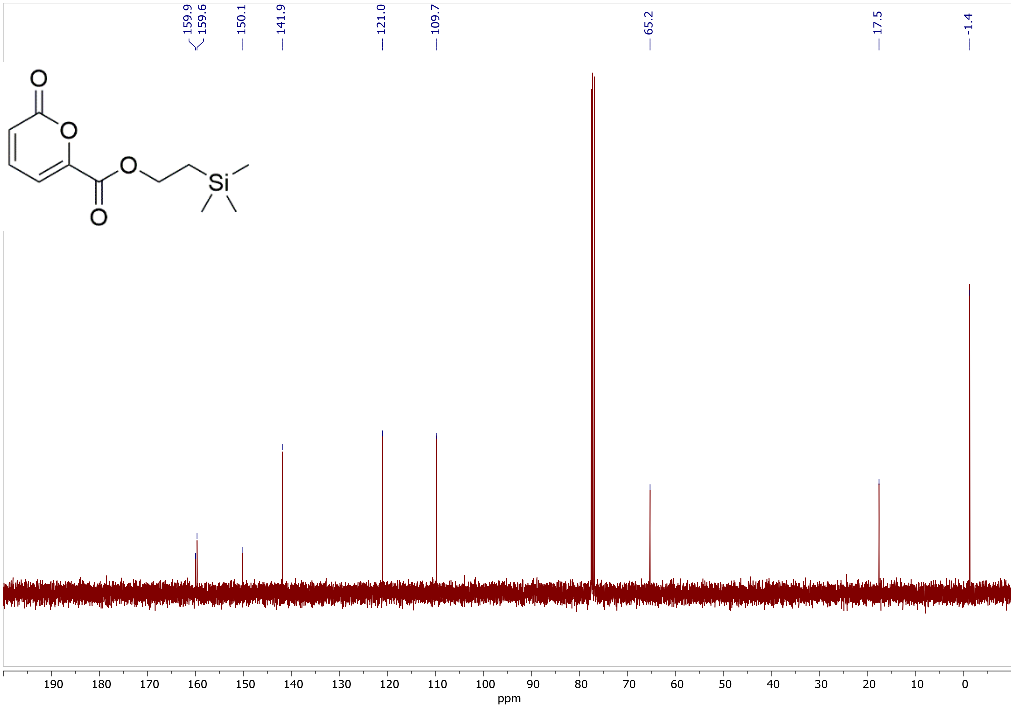

Figure S17. ^{1}H NMR of **11i** (400 MHz, $CDCl_3$).

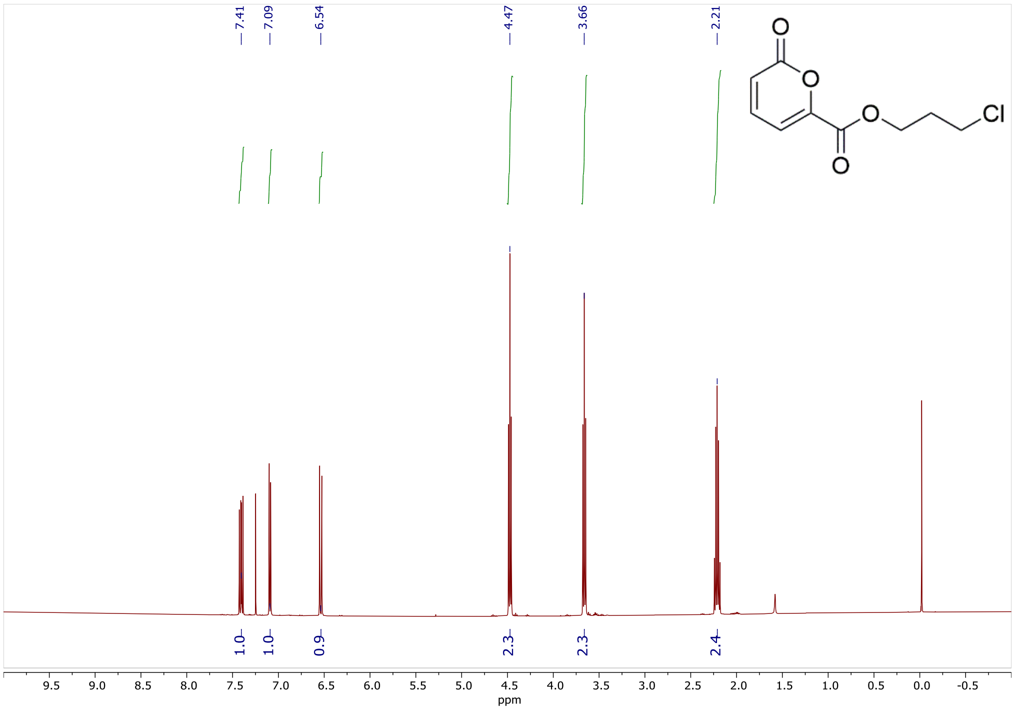

Figure S18. $^{13}C\{^{1}H\}$ NMR of **11i** (101 MHz, $CDCl_3$).

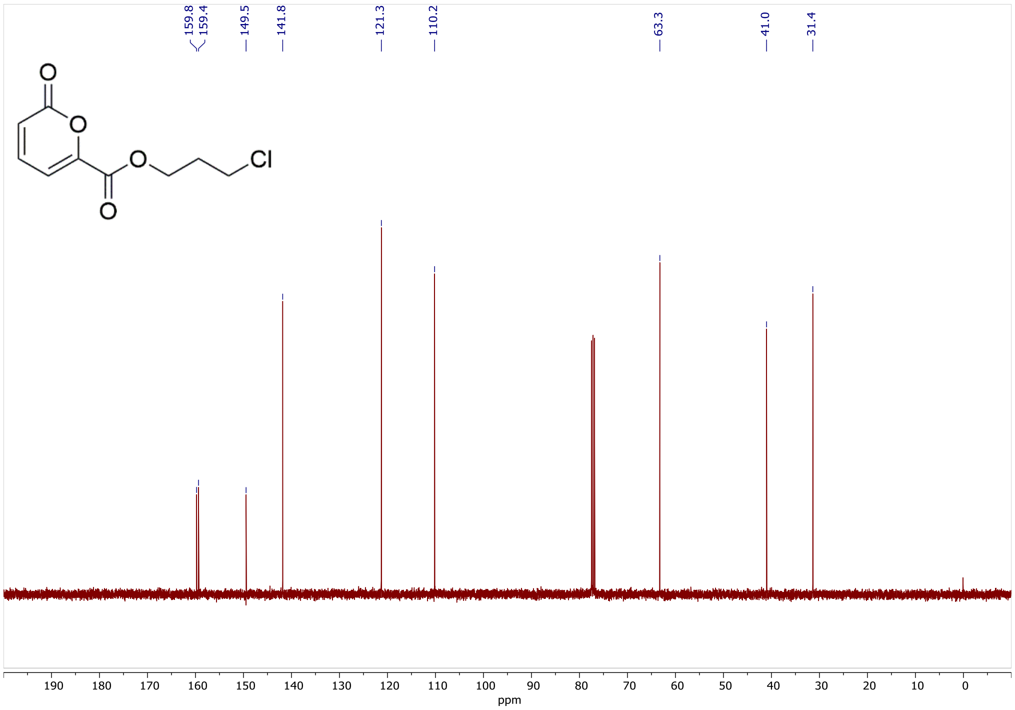

Figure S19. ^{1}H NMR of **11j** (400 MHz, $CDCl_3$).

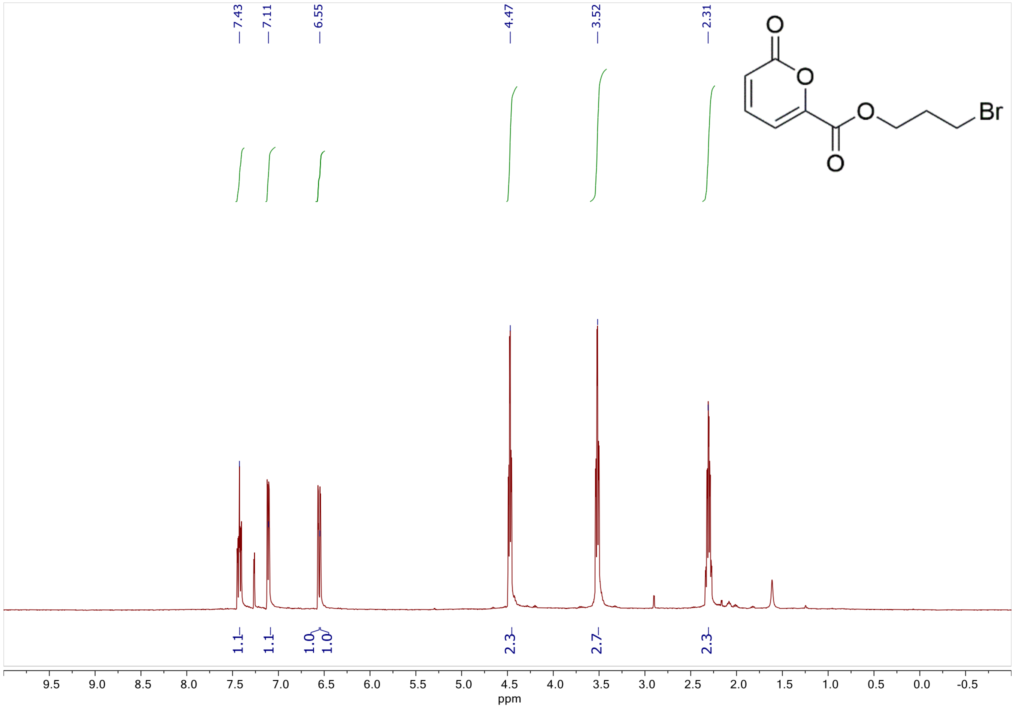

Figure S20. $^{13}C\{^{1}H\}$ NMR of **11j** (126 MHz, $CDCl_3$).

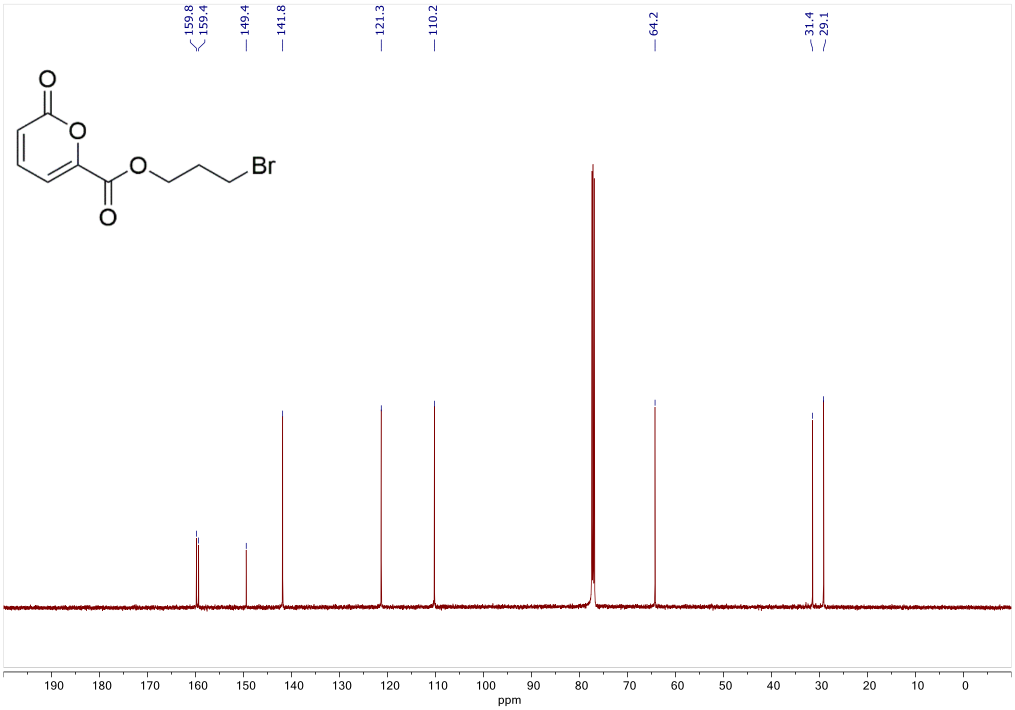

Figure S21. ^{1}H NMR of **11k** (400 MHz, CDCl$_3$).

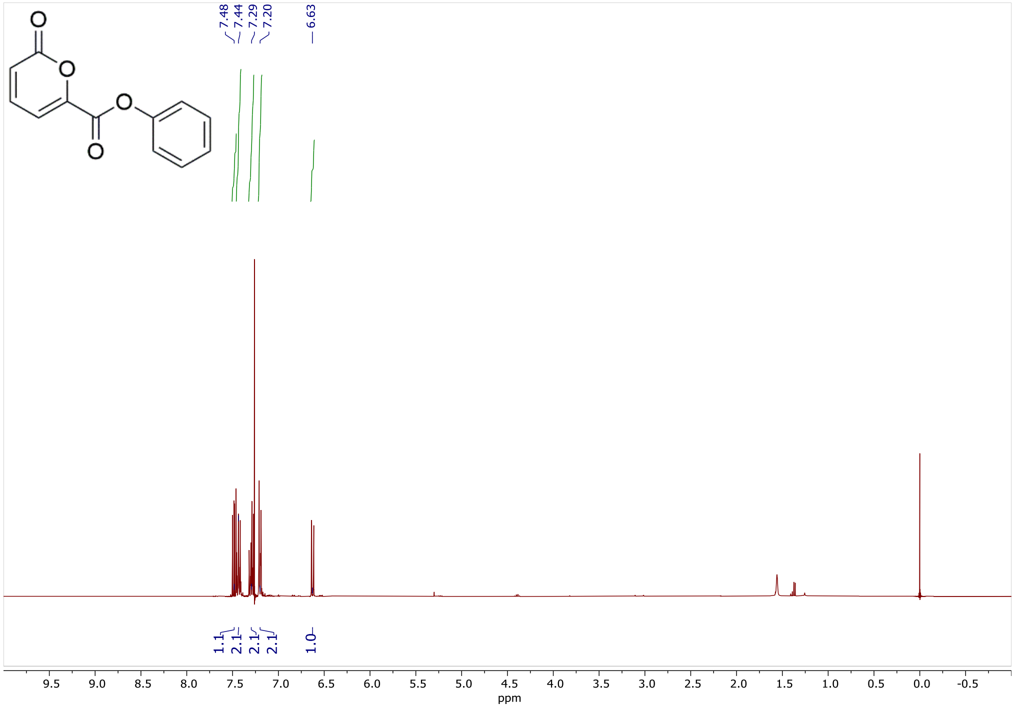

Figure S22. ^{13}C{^{1}H} NMR of **11k** (101 MHz, CDCl$_3$).

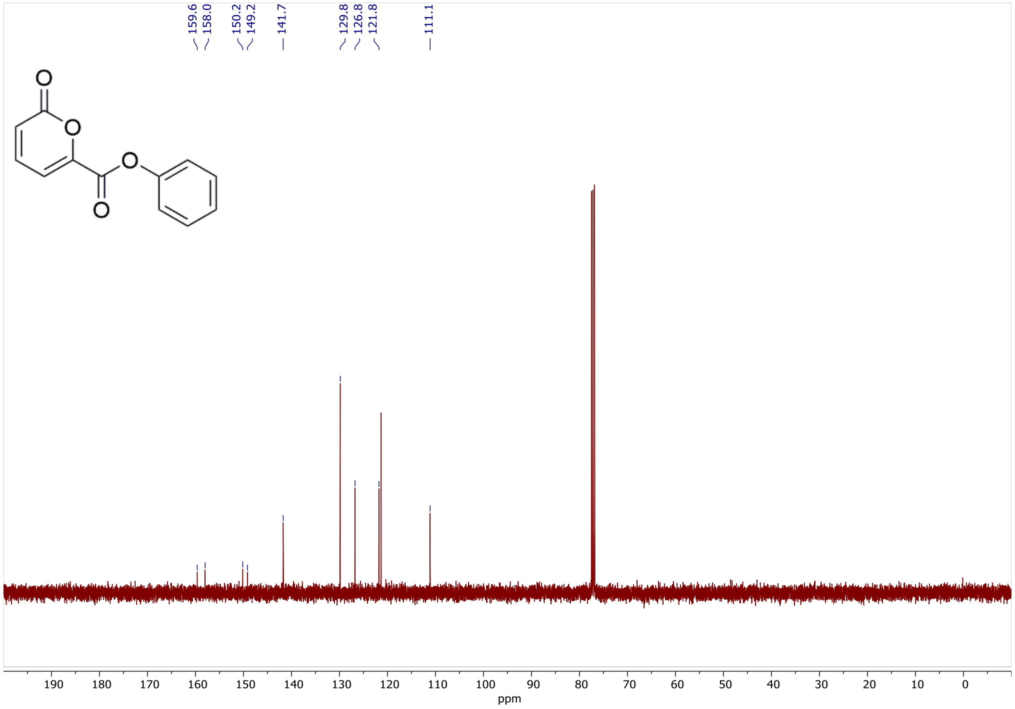

Figure S23. ^{1}H NMR of **11l** plus trace acetone (400 MHz, $CDCl_3$).

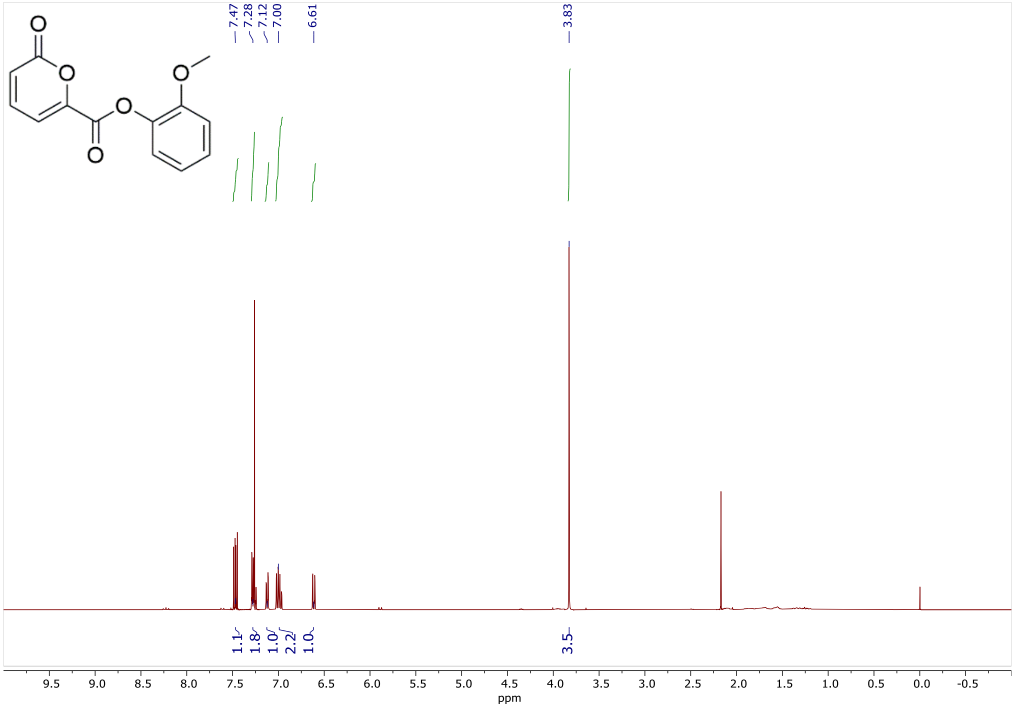

Figure S24. ^{13}C{^{1}H} NMR of **11l** (101 MHz, $CDCl_3$).

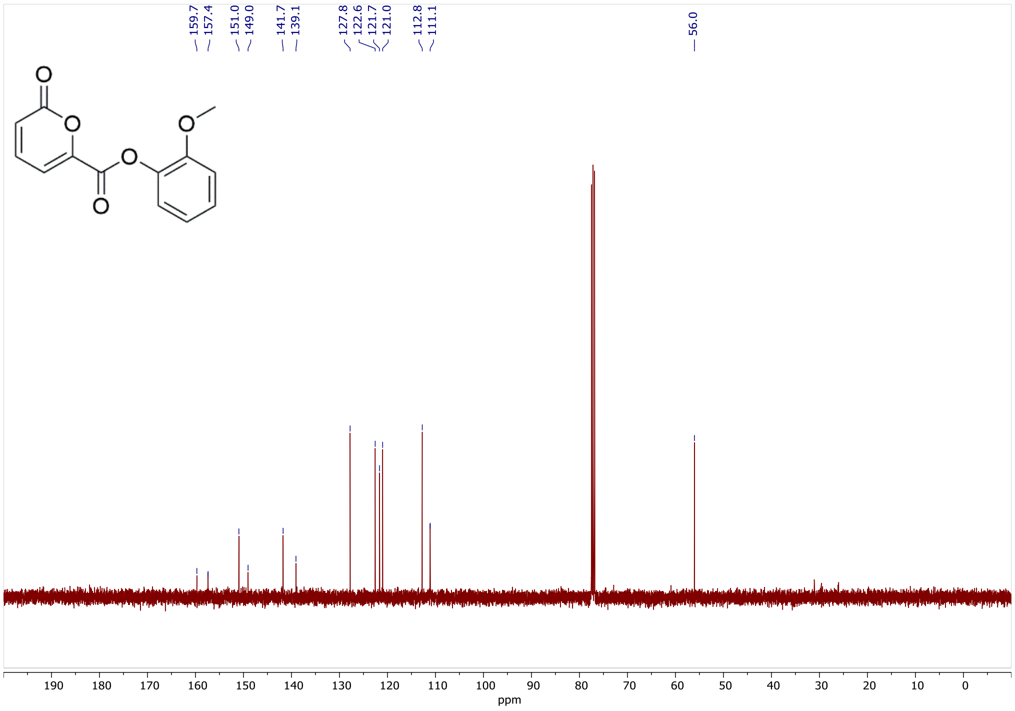

Figure S25. ^{1}H NMR of **11m** plus trace 4-methoxyphenol (400 MHz, $CDCl_3$).

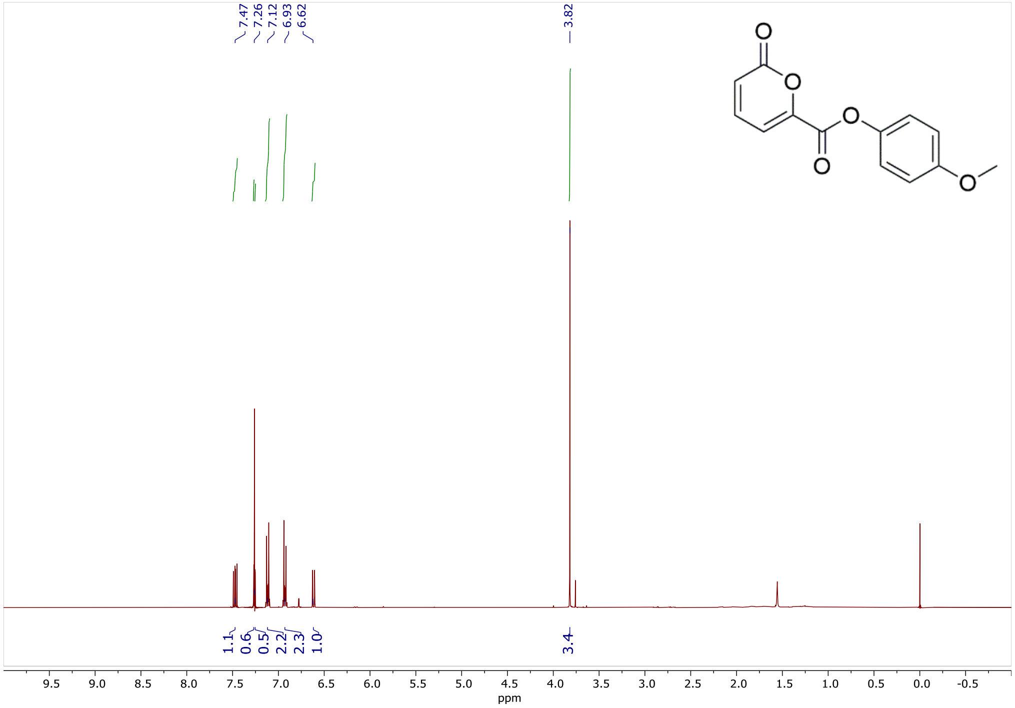

Figure S26. ^{13}C{^{1}H} NMR of **11m** (101 MHz, $CDCl_3$).

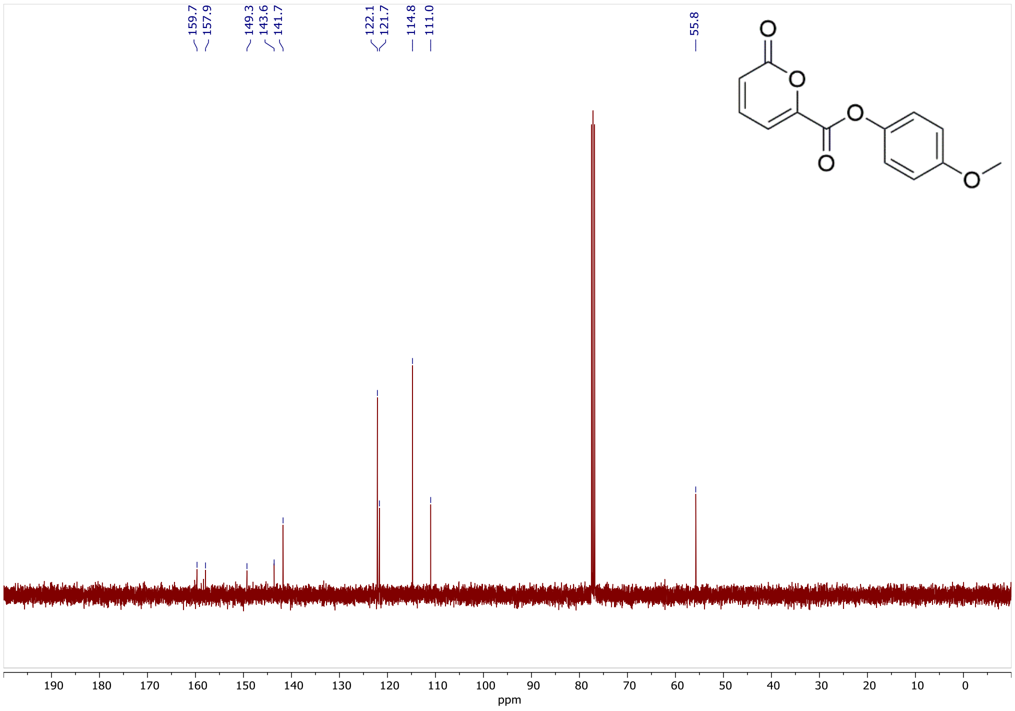

Figure S27. ^{1}H NMR of **11n** plus trace water (400 MHz, $CDCl_3$).

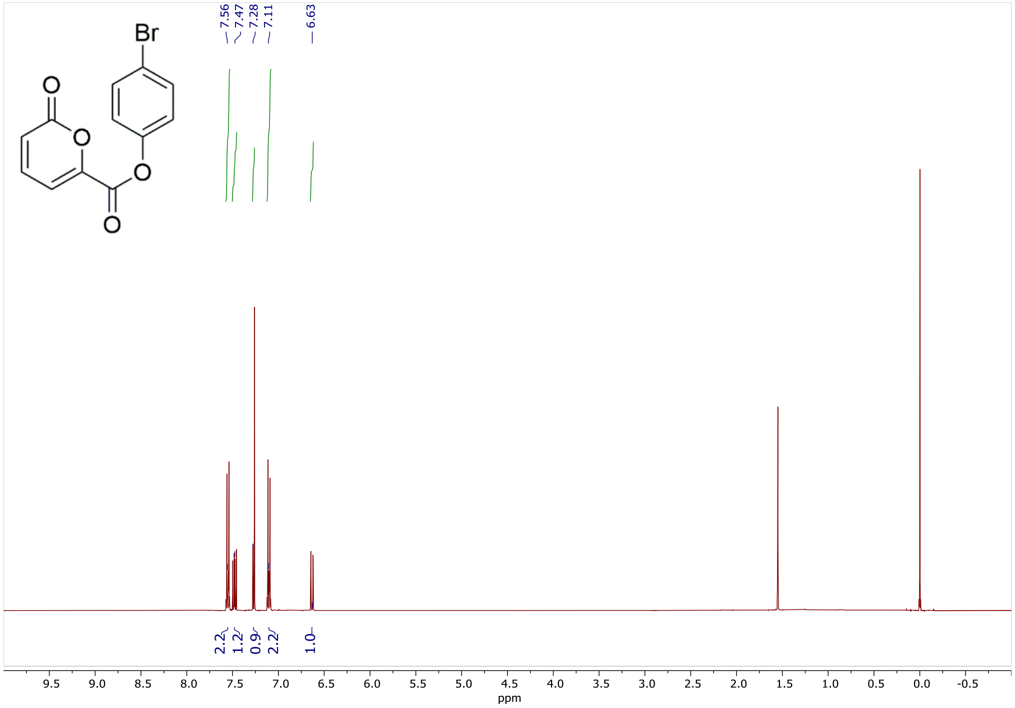

Figure S28. $^{13}C\{^{1}H\}$ NMR of **11n** (101 MHz, $CDCl_3$).

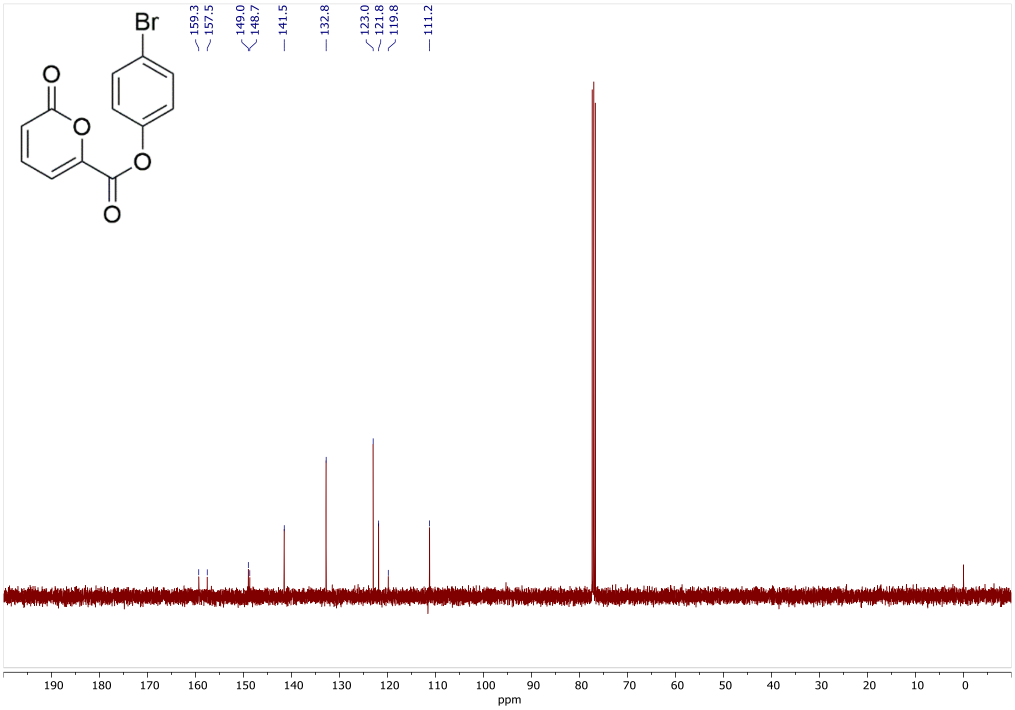

Figure S29. ^{1}H NMR of **11o** (400 MHz, $CDCl_3$).

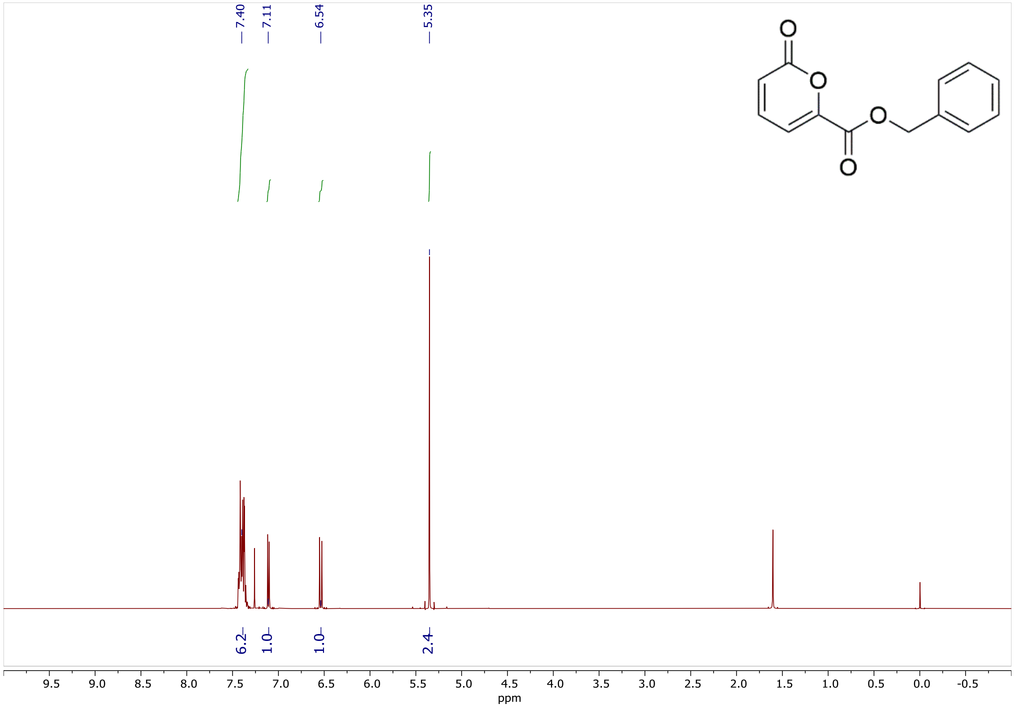

Figure S30. $^{13}C\{^{1}H\}$ NMR of **11o** (101 MHz, $CDCl_3$).

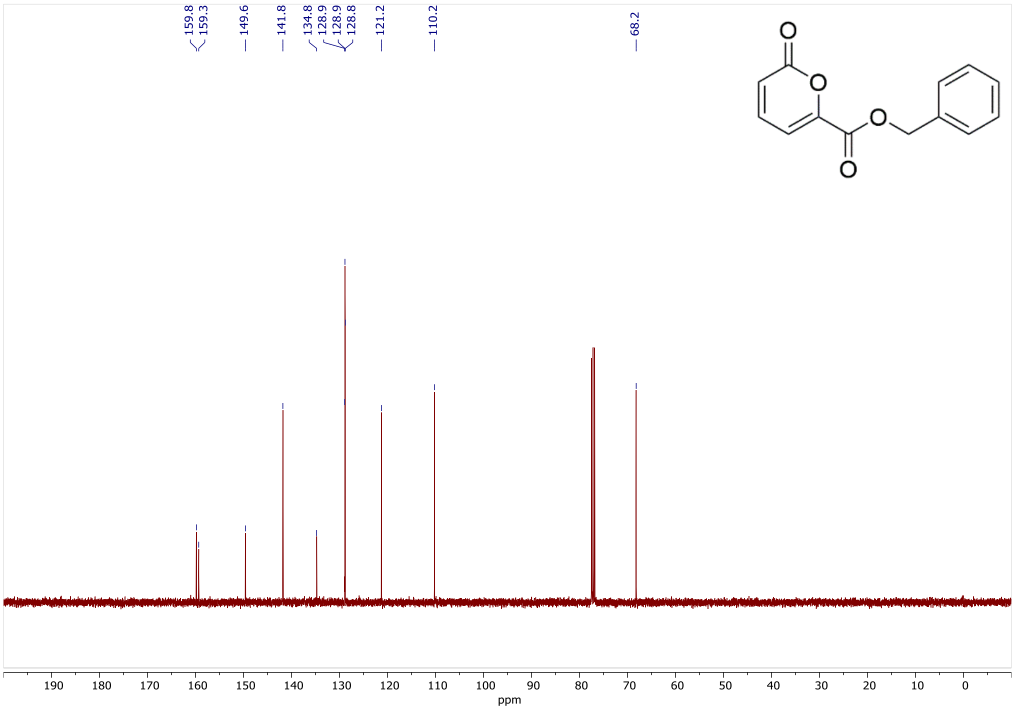

Figure S31. ^{1}H NMR of **11p** (400 MHz, $CDCl_3$).

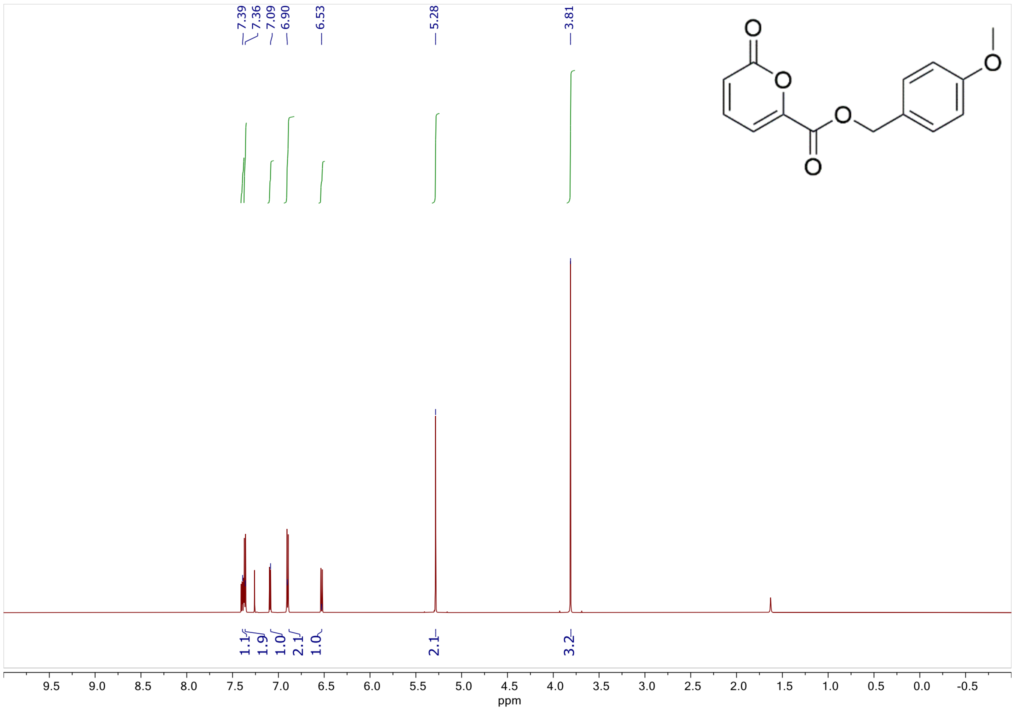

Figure S32. $^{13}C\{^{1}H\}$ NMR of **11p** (151 MHz, $CDCl_3$).

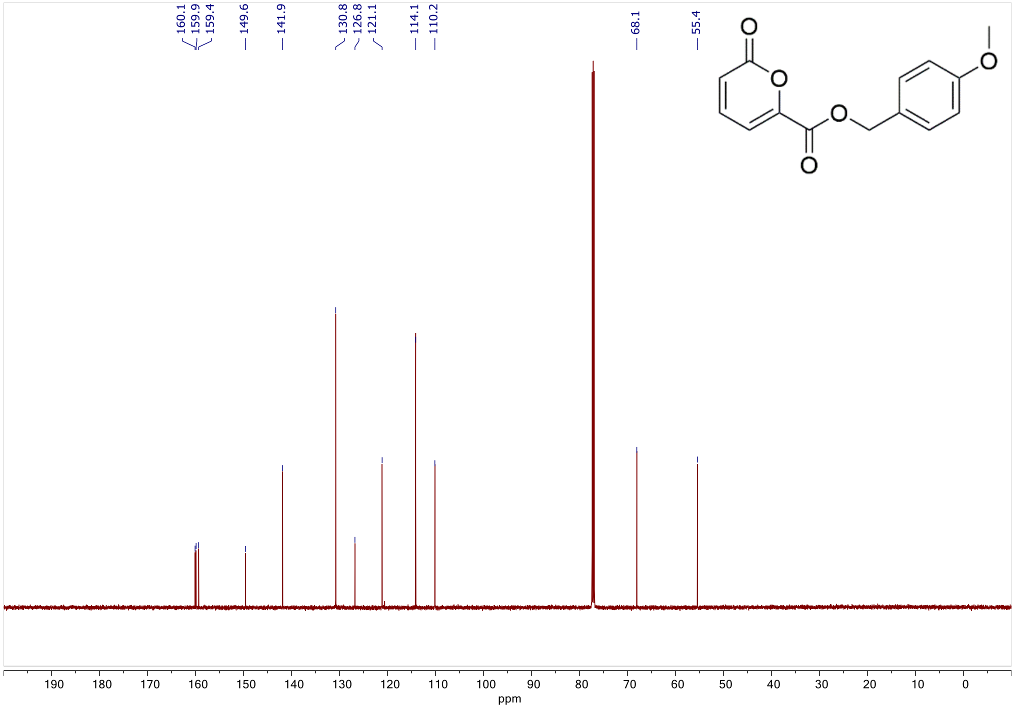

Figure S33. ^{1}H NMR of **11q** (400 MHz, $CDCl_3$).

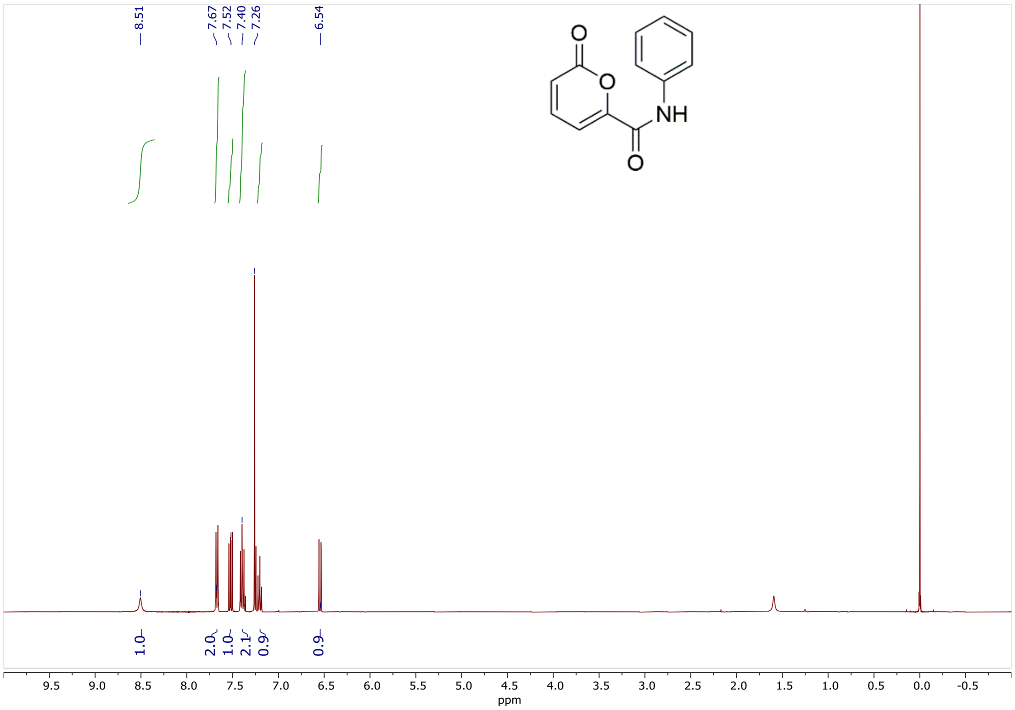

Figure S34. $^{13}C\{^{1}H\}$ NMR of **11q** (126 MHz, $CDCl_3$).

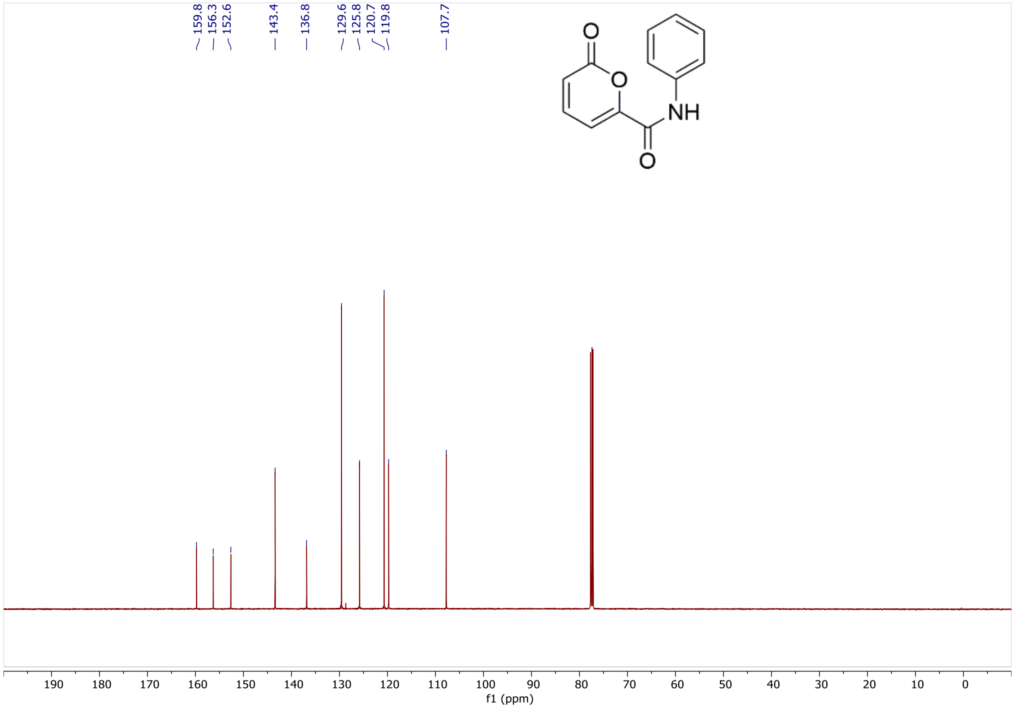

Figure S35. ^{1}H NMR of **11r** (400 MHz, $CDCl_3$).

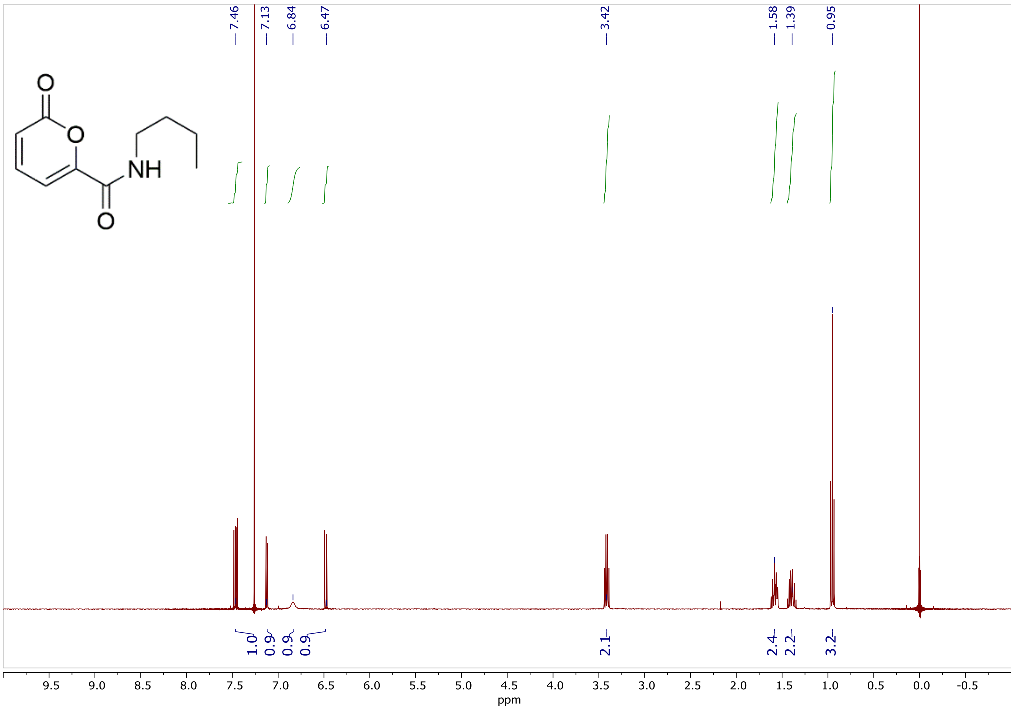

Figure S36. $^{13}C\{^{1}H\}$ NMR of **11r** (126 MHz, $CDCl_3$).

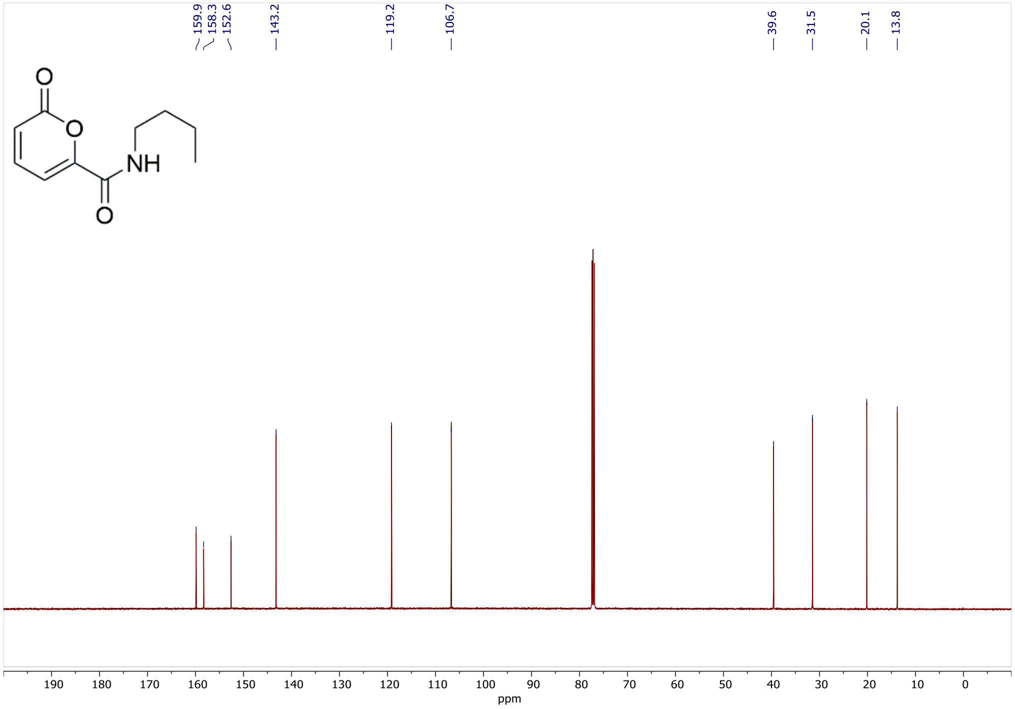

Figure S37. ^{1}H NMR of **11s** plus trace acetone (400 MHz, $CDCl_3$).

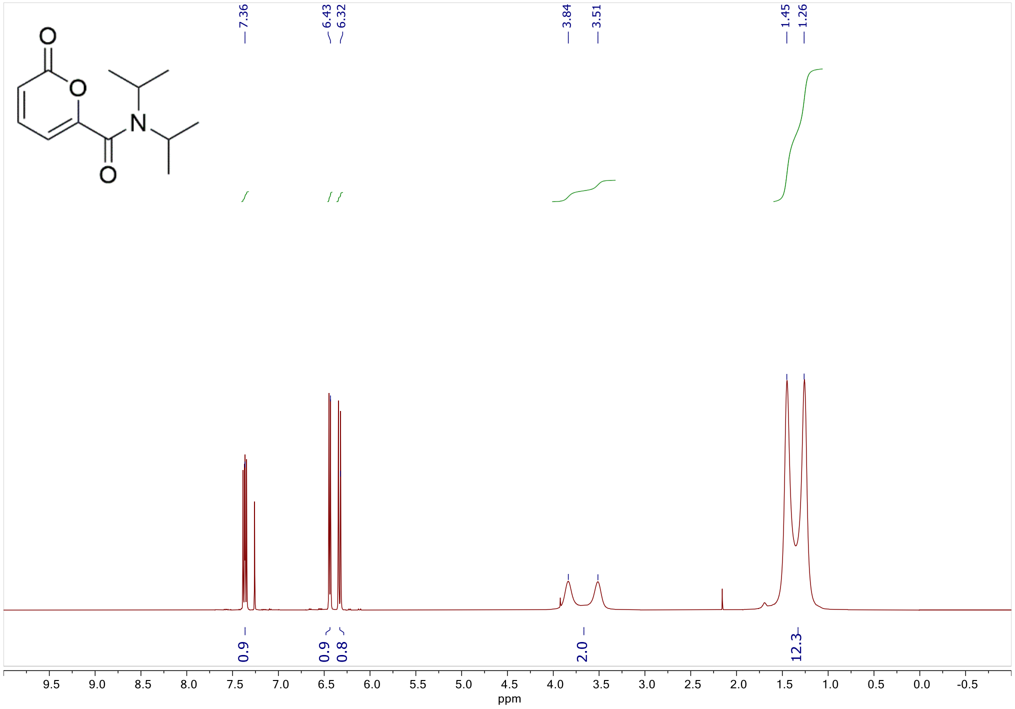

Figure S38. ^{13}C{^{1}H} NMR of **11s** (126 MHz, $CDCl_3$).

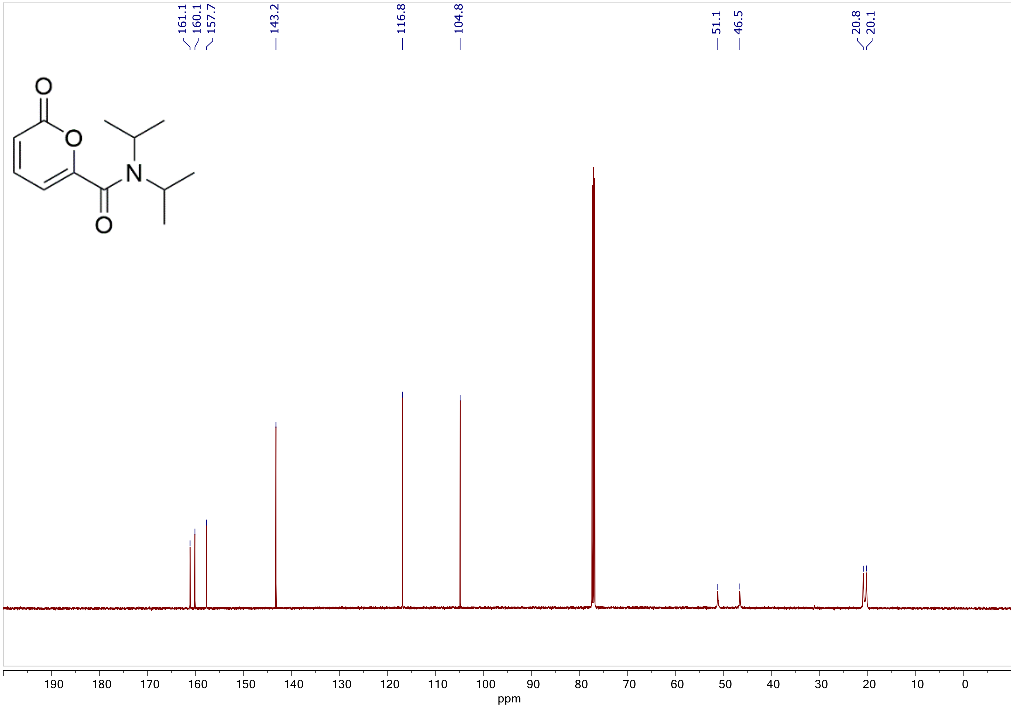

Figure S39. ^{1}H NMR of **11t** (400 MHz, $CDCl_3$).

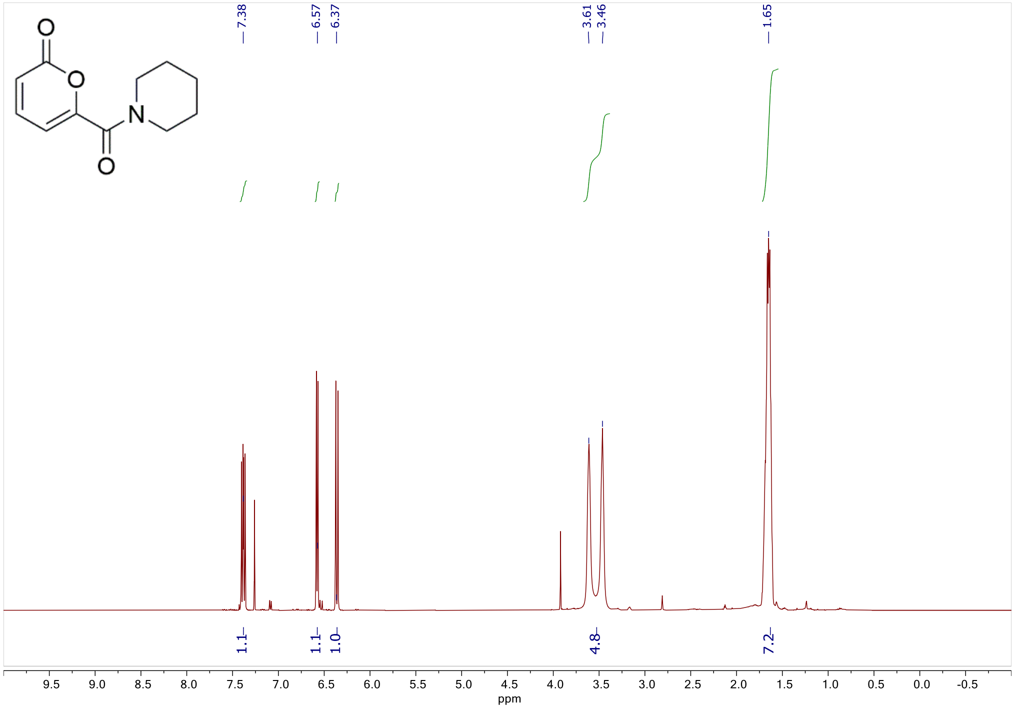

Figure S40. $^{13}C\{^{1}H\}$ NMR of **11t** (126 MHz, $CDCl_3$).

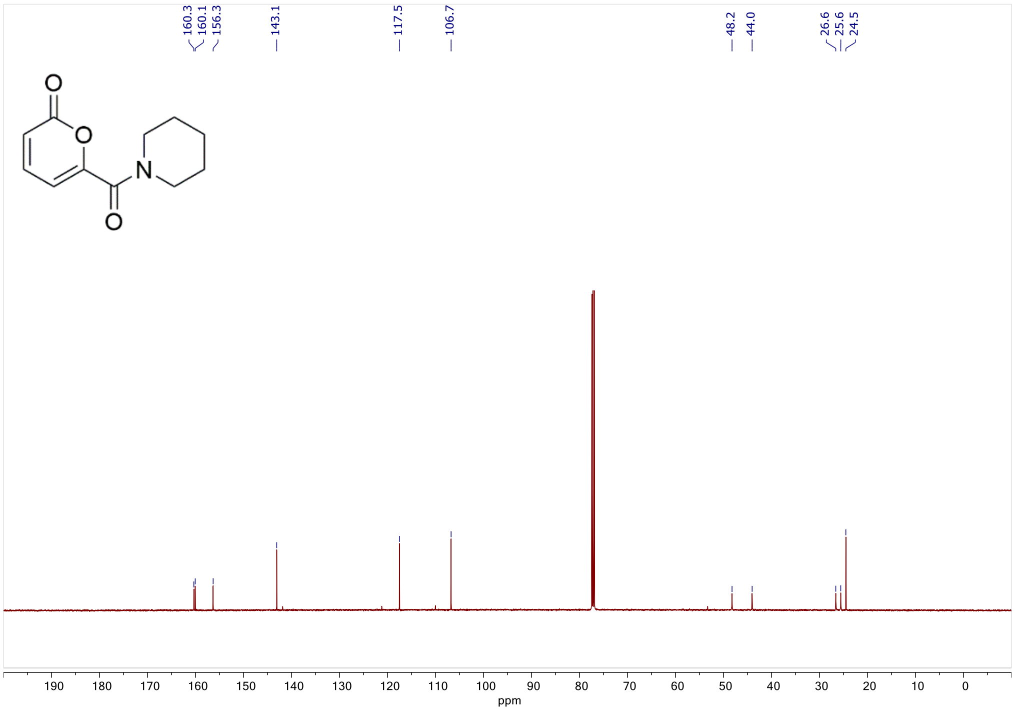

Figure S41. ^{1}H NMR of **11u** (400 MHz, CD_3OD).

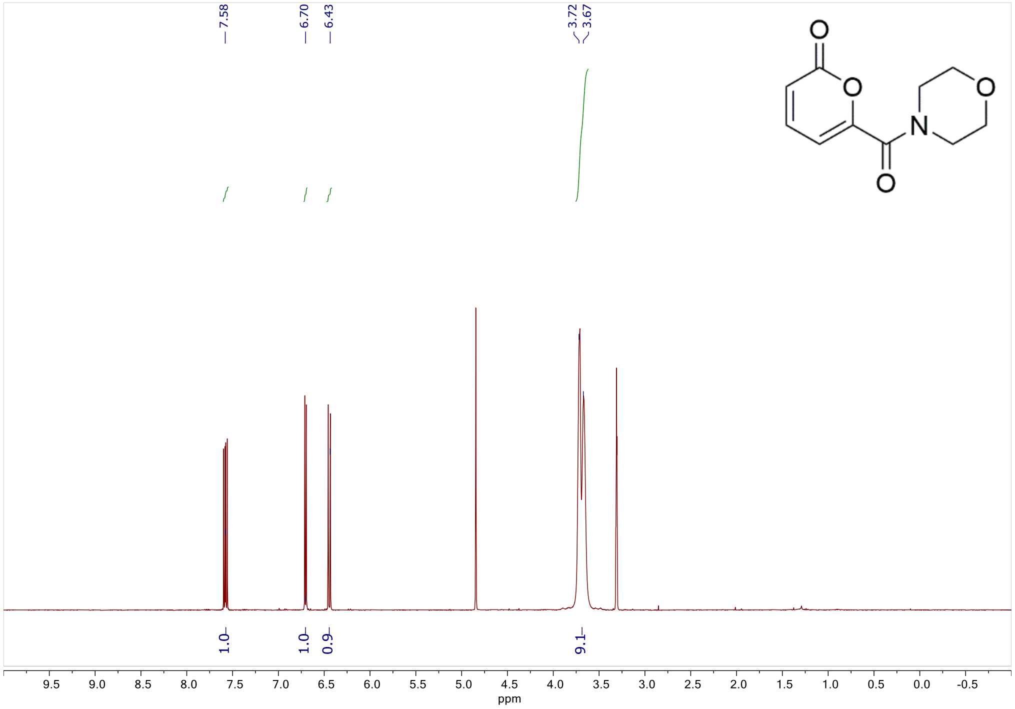

Figure S42. $^{13}C\{^{1}H\}$ NMR of **11u** (151 MHz, CD_3OD).

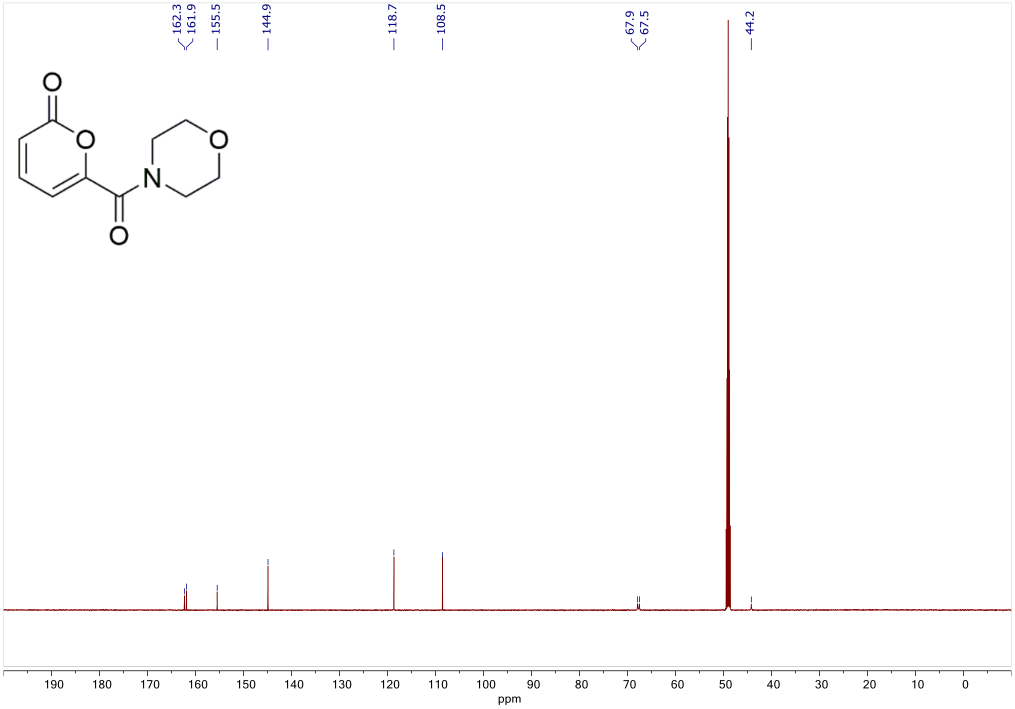

Figure S43. ^{1}H NMR of **11v** (400 MHz, $CDCl_3$).

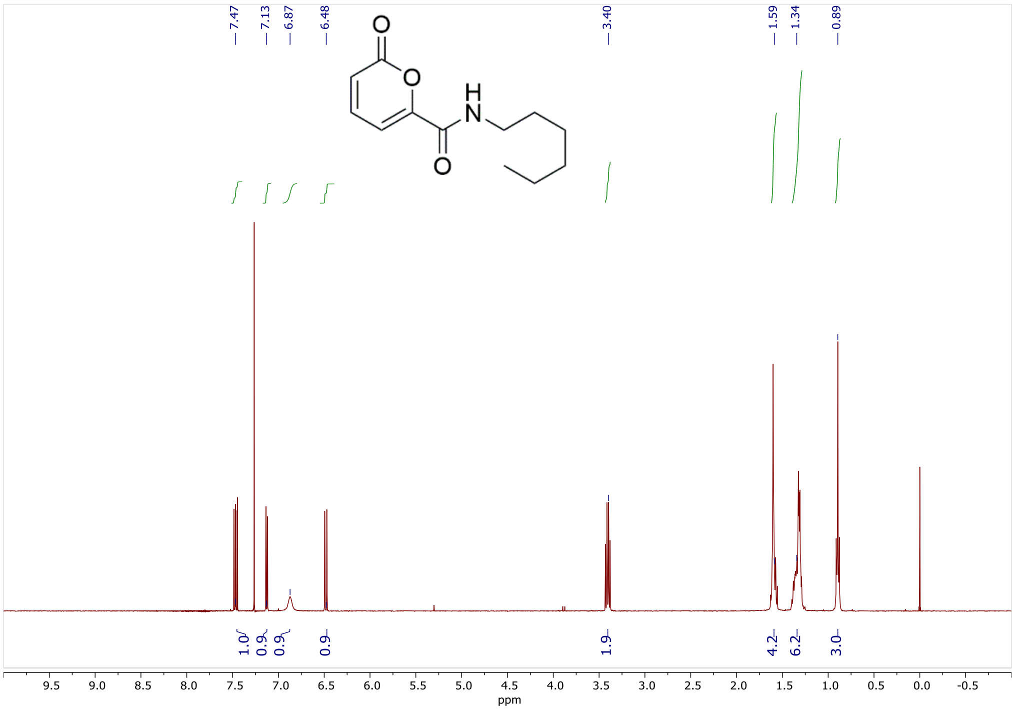

Figure S44. $^{13}C\{^{1}H\}$ NMR of **11v** (101 MHz, $CDCl_3$).

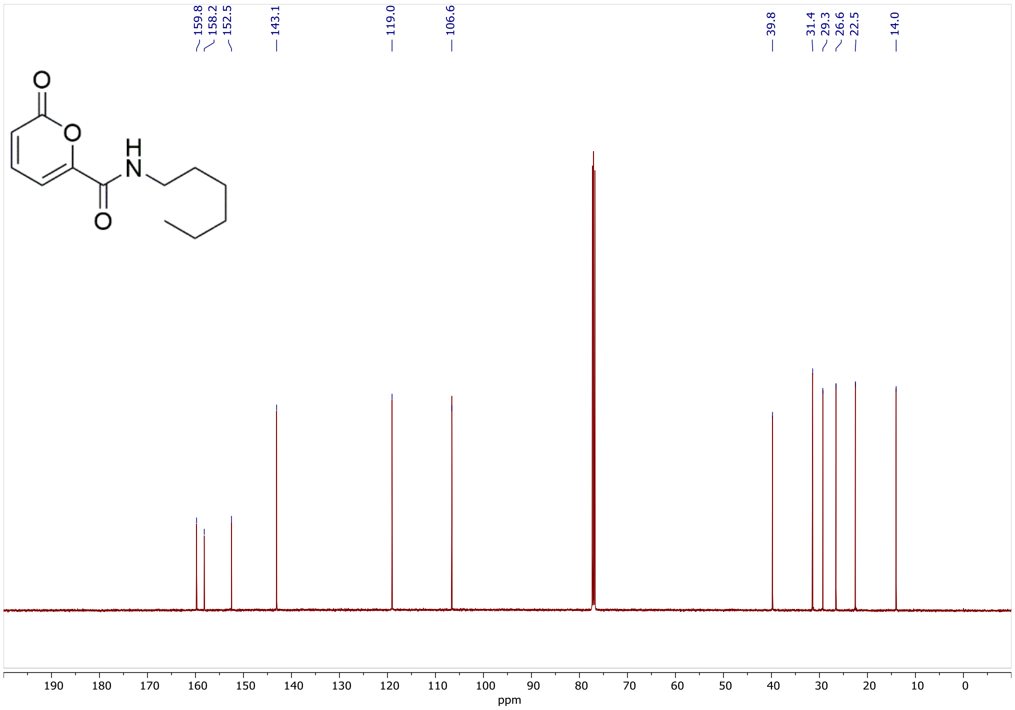

Figure S45. ^{1}H NMR of **12a** (400 MHz, $CDCl_3$).

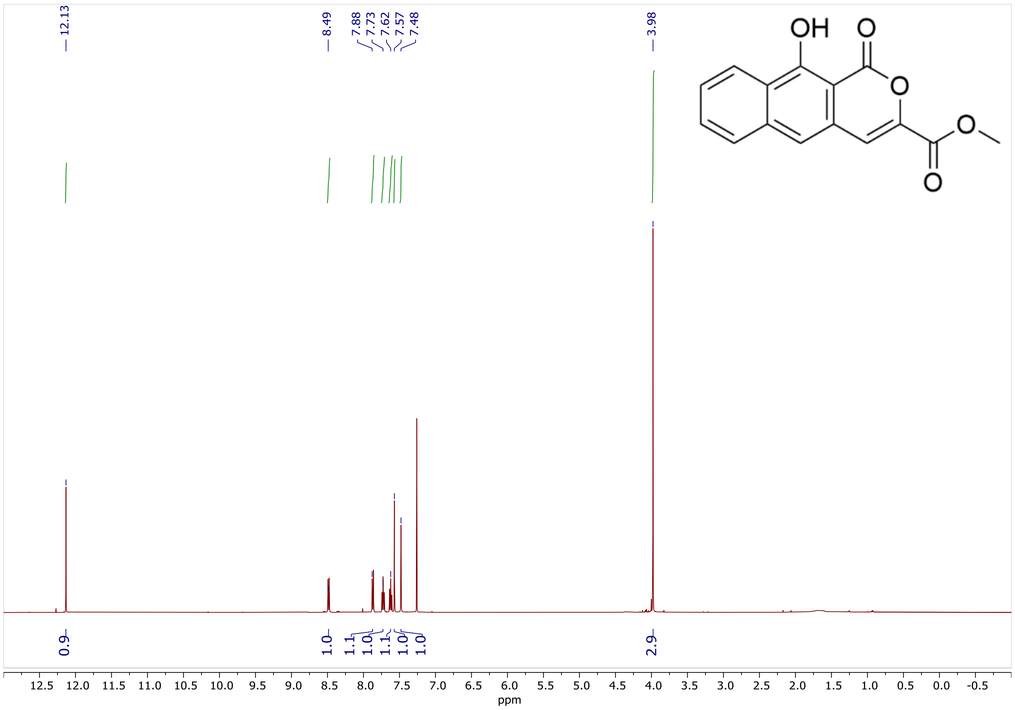

Figure S46. $^{13}C\{^1H\}$ NMR of **12a** (126 MHz, $CDCl_3$).

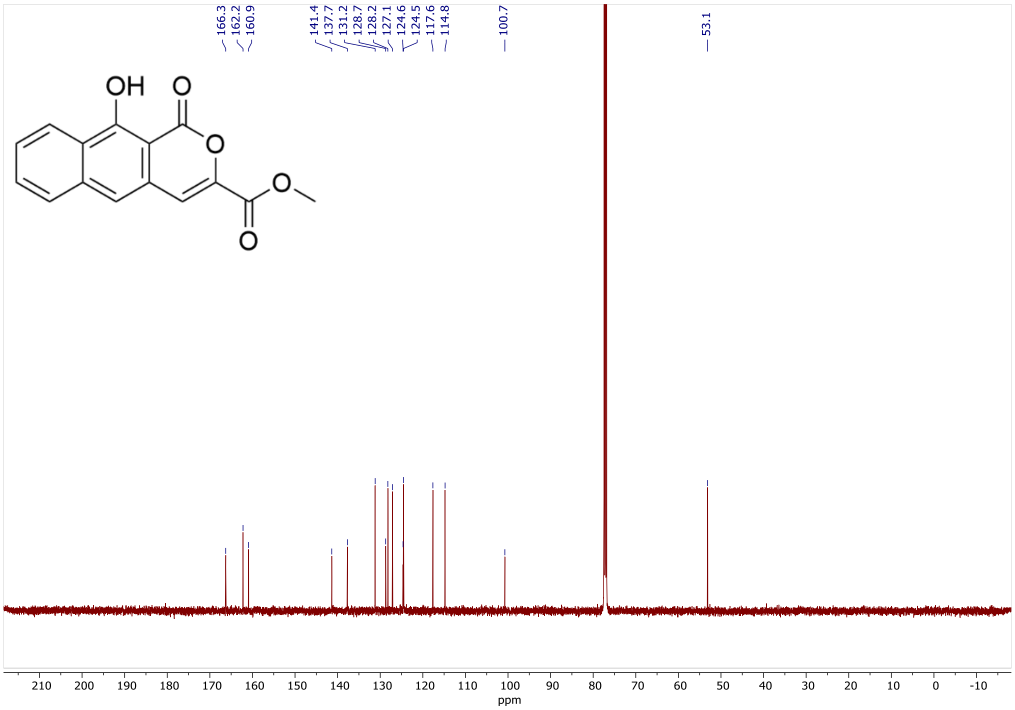

Figure S47. ^{1}H NMR of **12b** (400 MHz, $CDCl_3$).

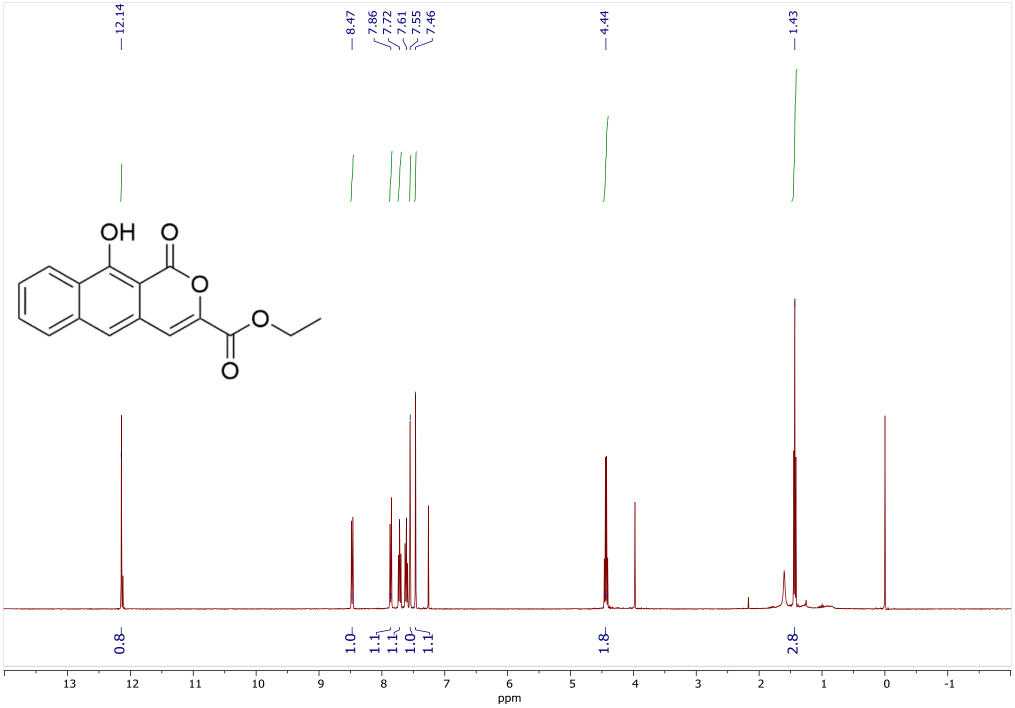

Figure S48. ^{13}C{^{1}H} NMR of **12b** (101 MHz, $CDCl_3$).

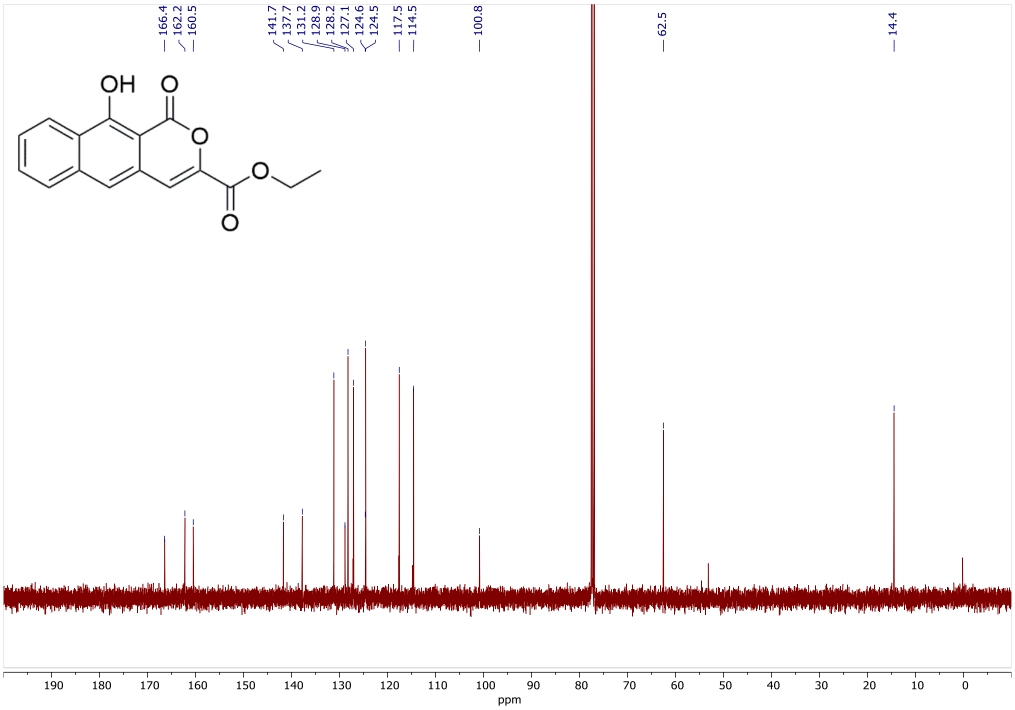

Figure S49. ^{1}H NMR of **12c** plus grease (400 MHz, CDCl$_3$).

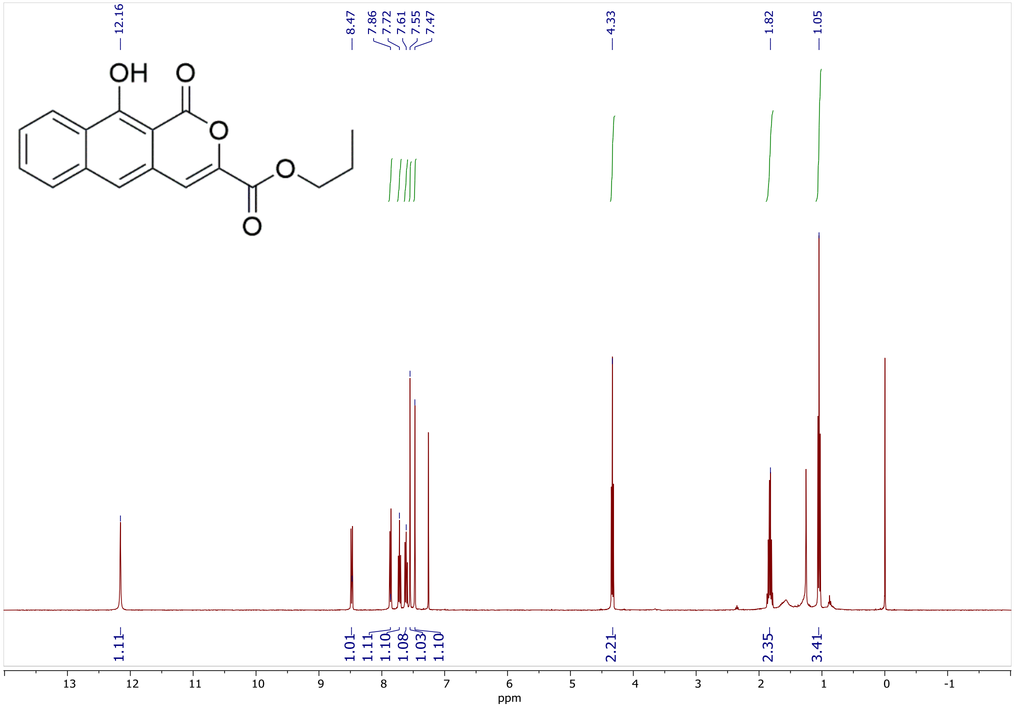

Figure S50. ^{13}C{^{1}H} NMR of **12c** plus grease (101 MHz, CDCl$_3$).

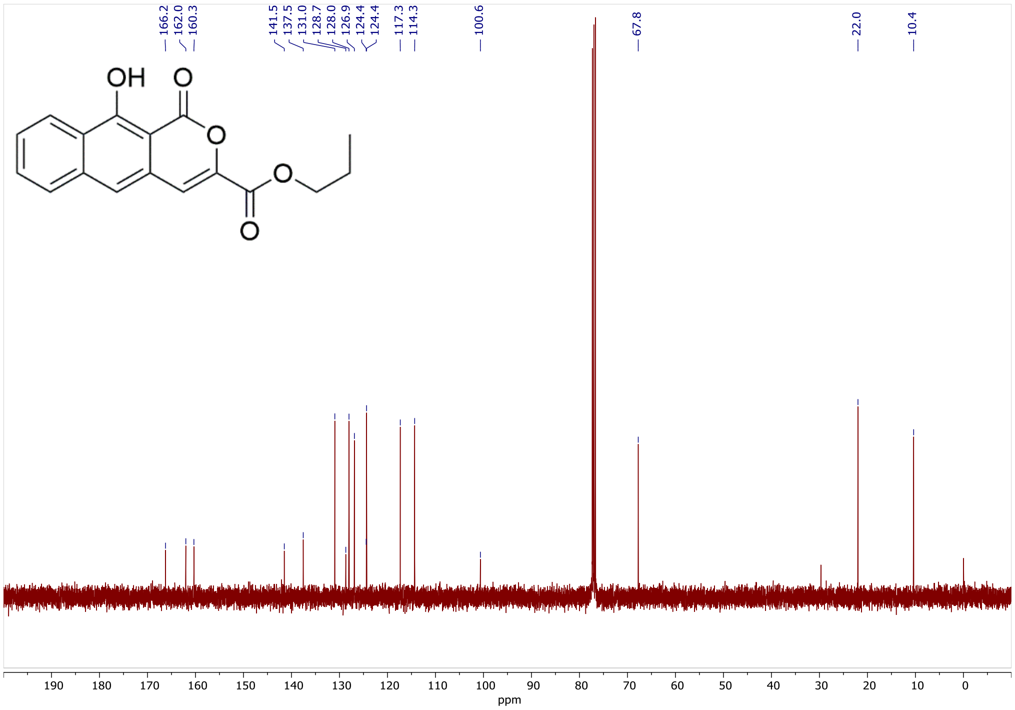

Figure S51. ^{1}H NMR of **12d** (400 MHz, $CDCl_3$).

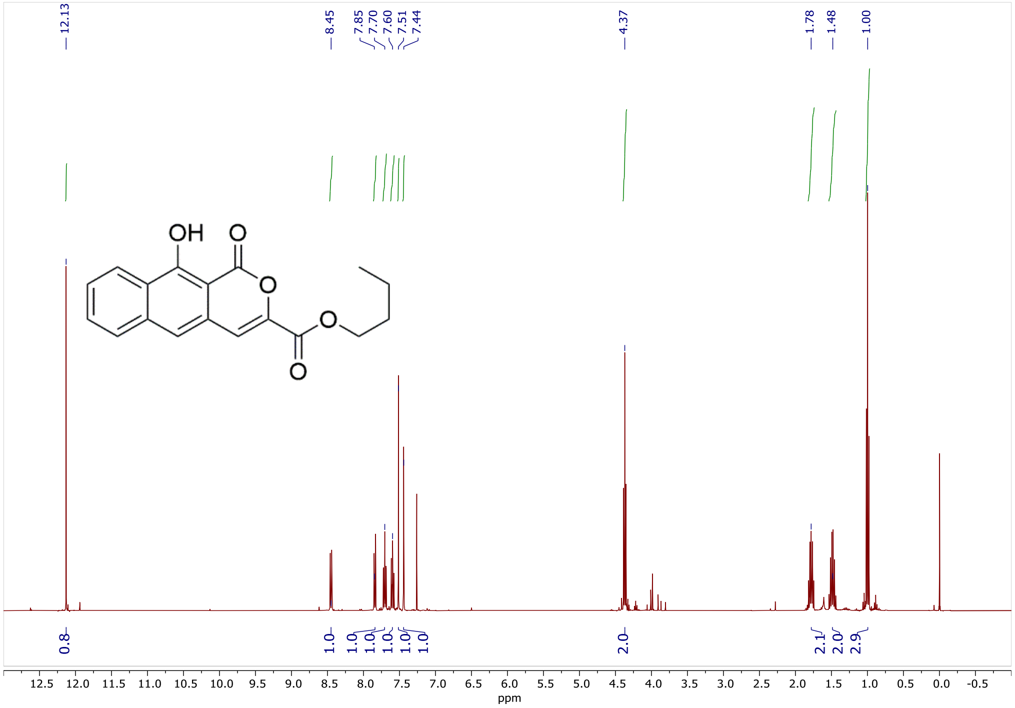

Figure S52. $^{13}C\{^{1}H\}$ NMR of **12d** (101 MHz, $CDCl_3$).

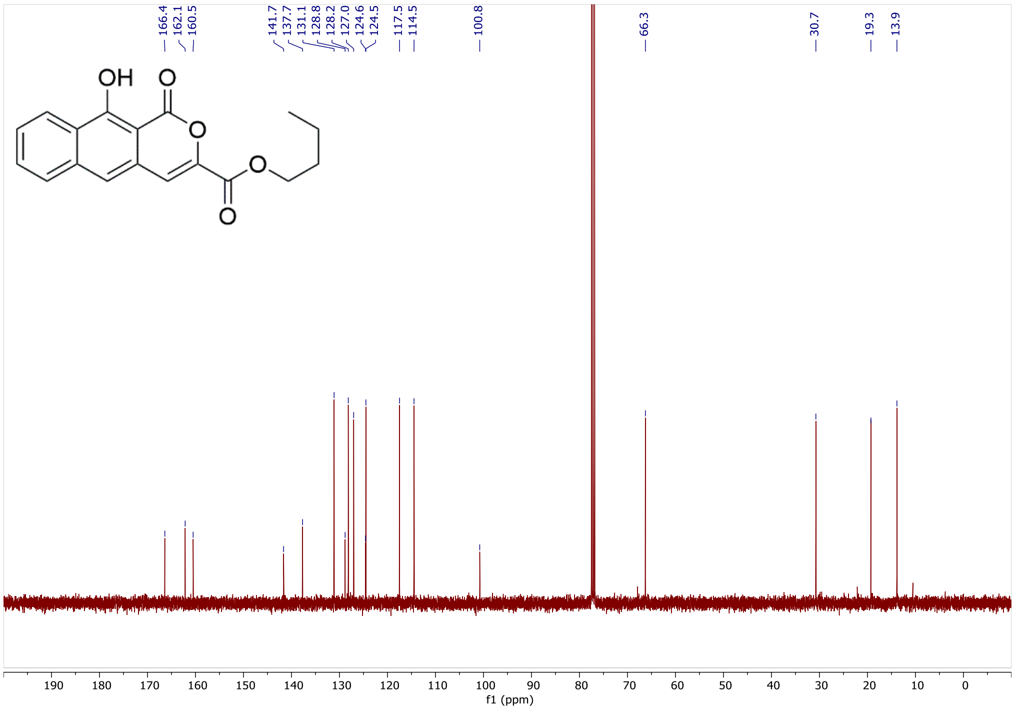

Figure S53. ^{1}H NMR of **12e** (400 MHz, $CDCl_3$).

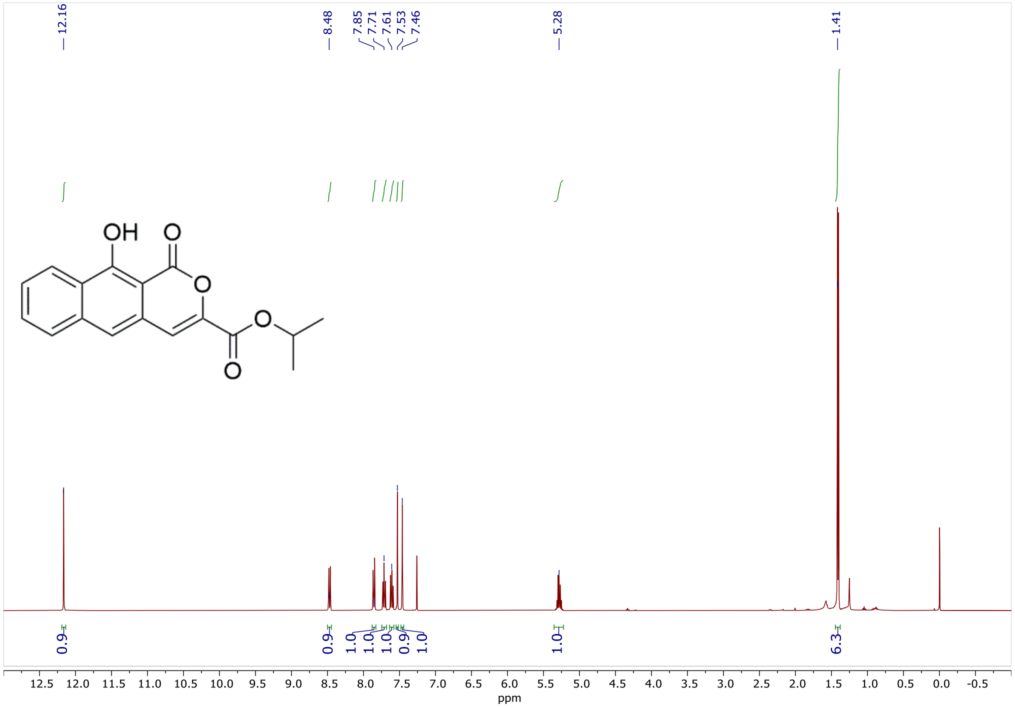

Figure S54. $^{13}C\{^{1}H\}$ NMR of **12e** (101 MHz, $CDCl_3$).

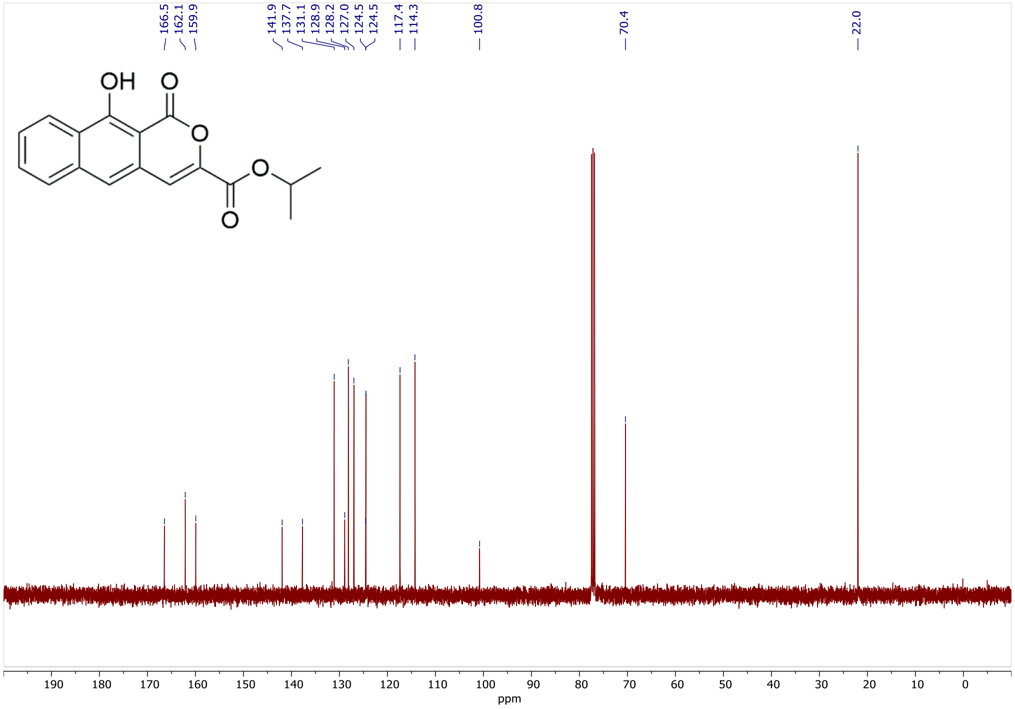

Figure S55. ^{1}H NMR of **12g** plus grease (400 MHz, $CDCl_3$).

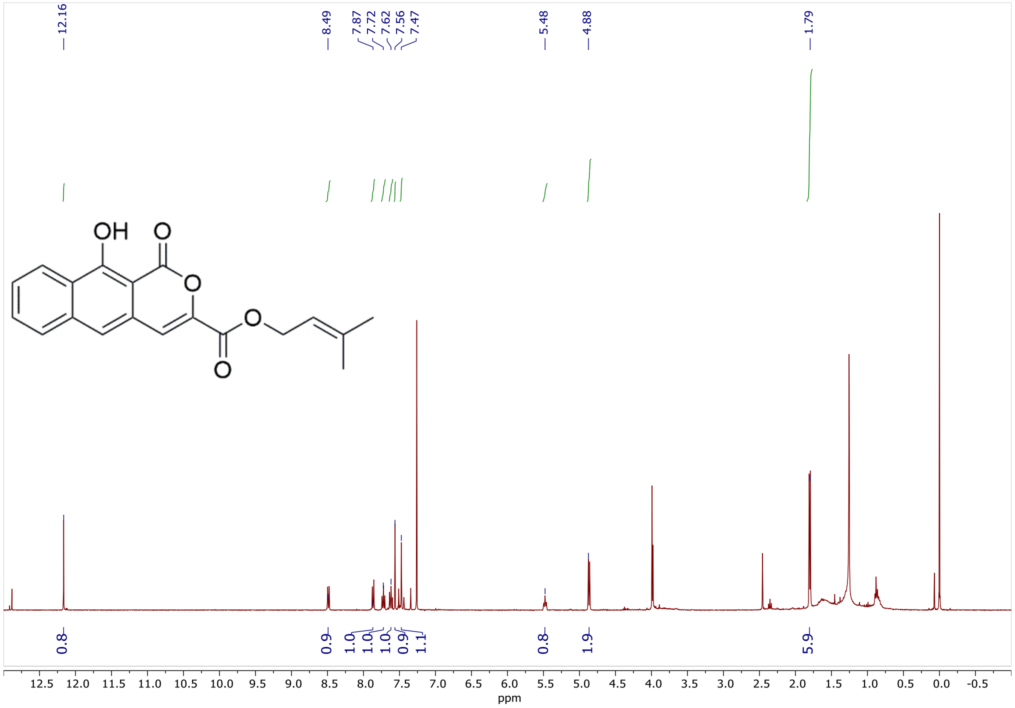

Figure S56. $^{13}C\{^{1}H\}$ NMR of **12g** plus grease (101 MHz, $CDCl_3$).

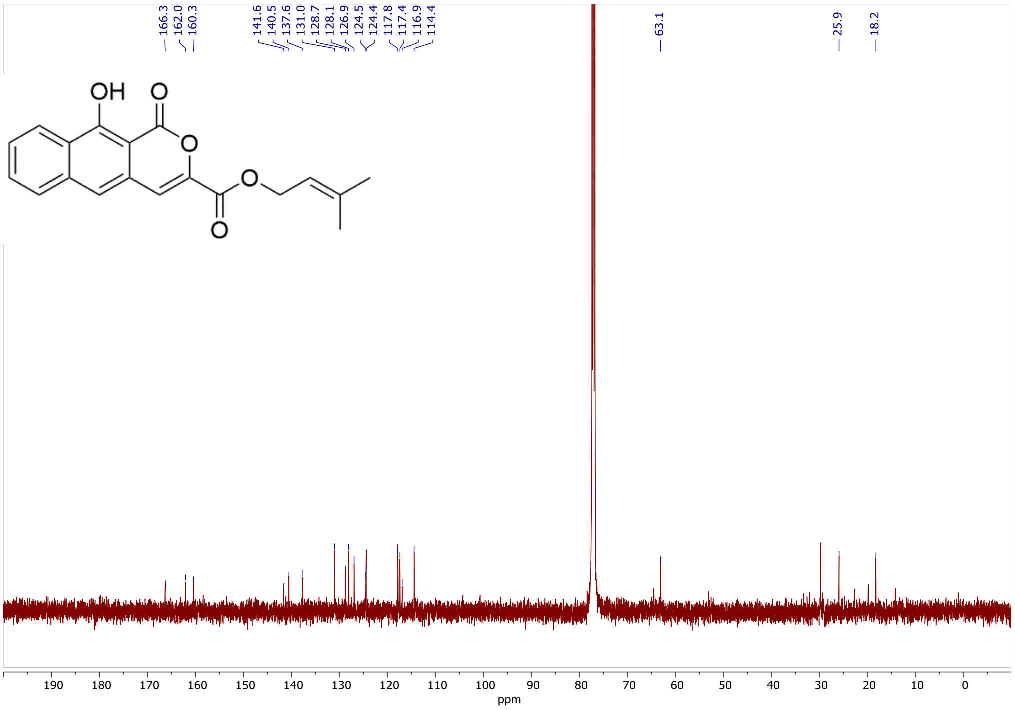

Figure S57. ^{1}H NMR of **12h** plus grease (400 MHz, $CDCl_3$).

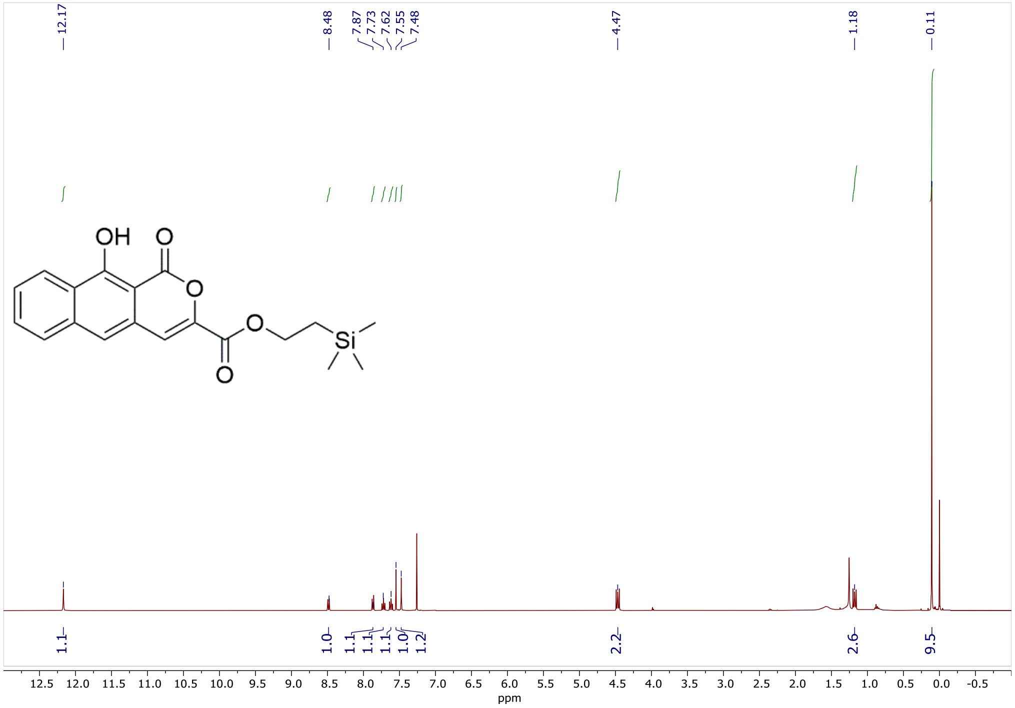

Figure S58. $^{13}C\{^{1}H\}$ NMR of **12h** plus grease (126 MHz, $CDCl_3$).

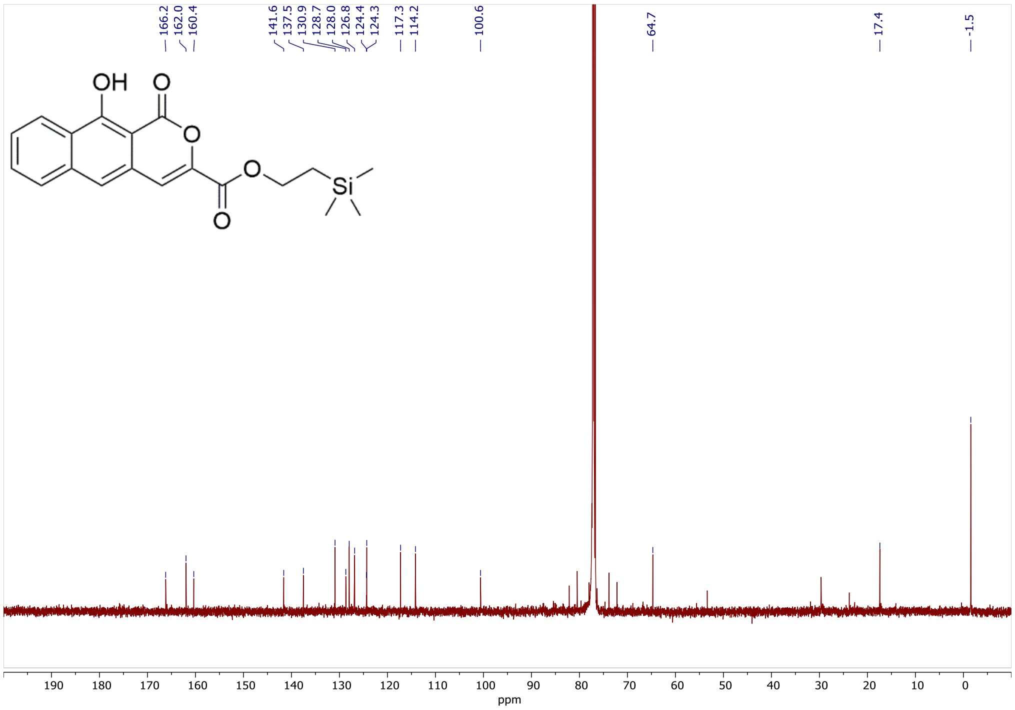

Figure S59. ^{1}H NMR of **12i** (400 MHz, $CDCl_3$).

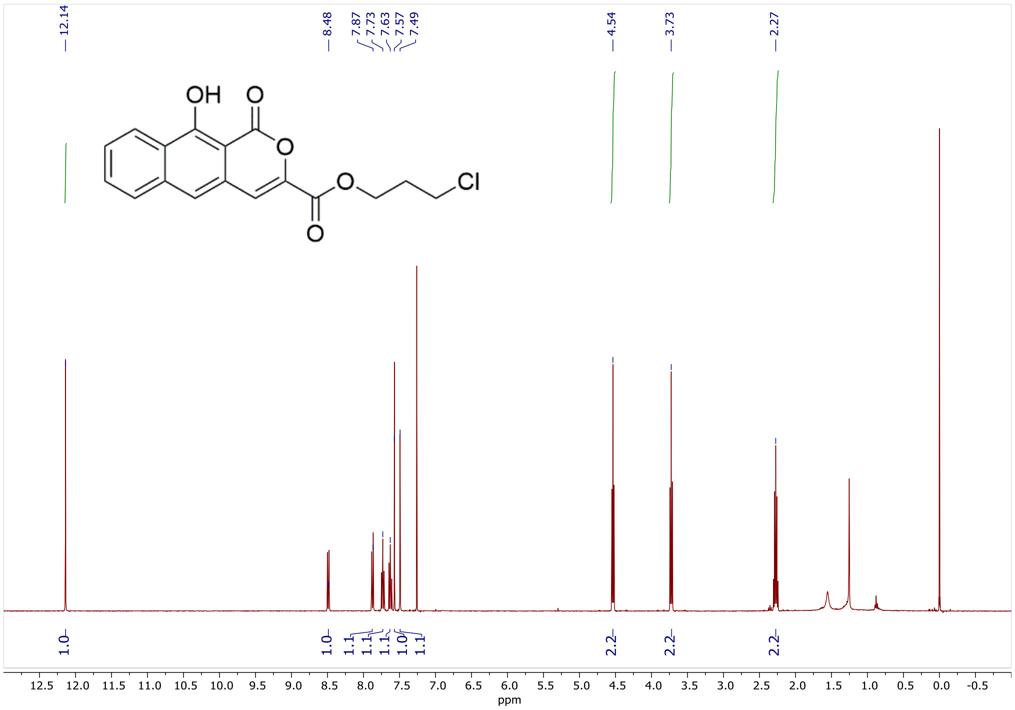

Figure S60. ^{13}C{^{1}H} NMR of **12i** (101 MHz, $CDCl_3$).

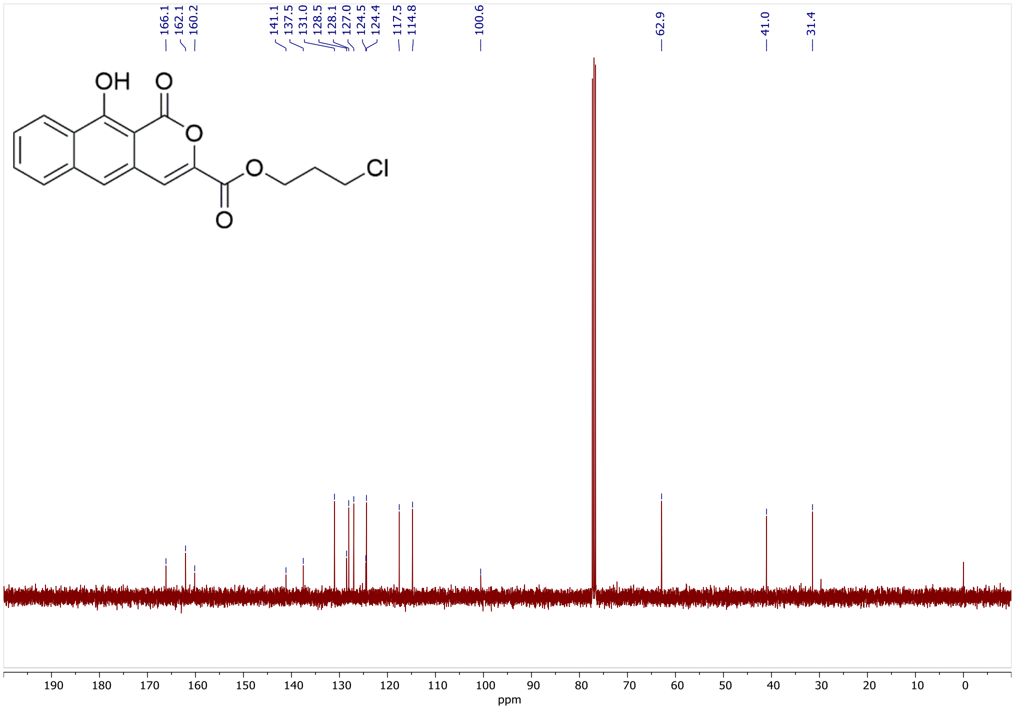

Figure S61. ^{1}H NMR of **12j** plus grease (500 MHz, $CDCl_3$).

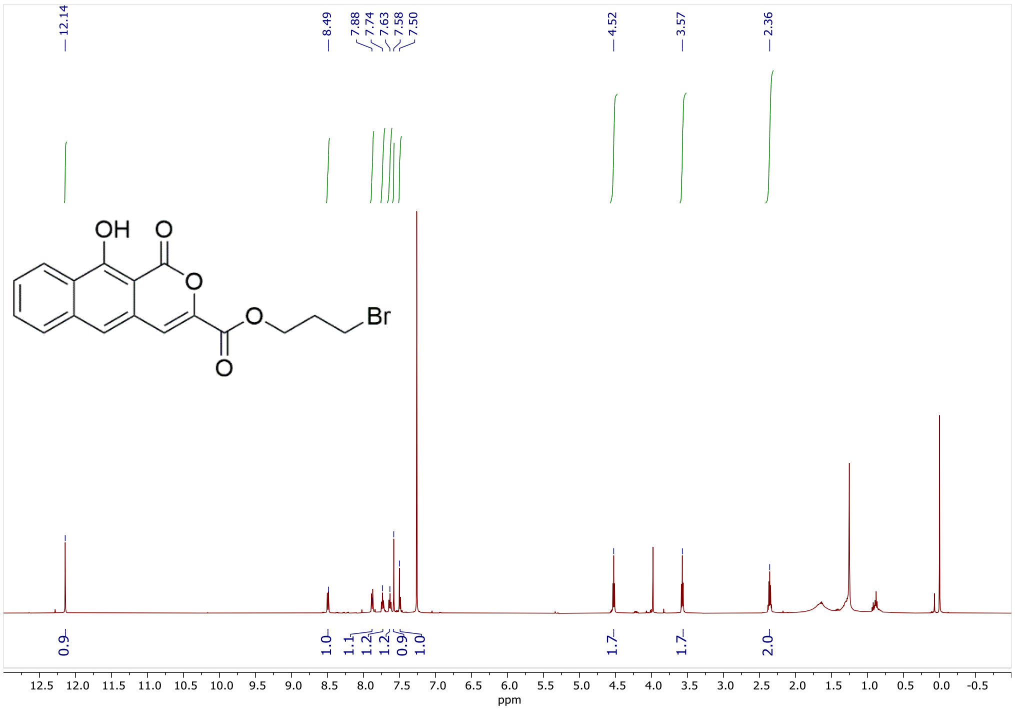

Figure S62. $^{13}C\{^{1}H\}$ NMR of **12j** plus grease (126 MHz, $CDCl_3$).

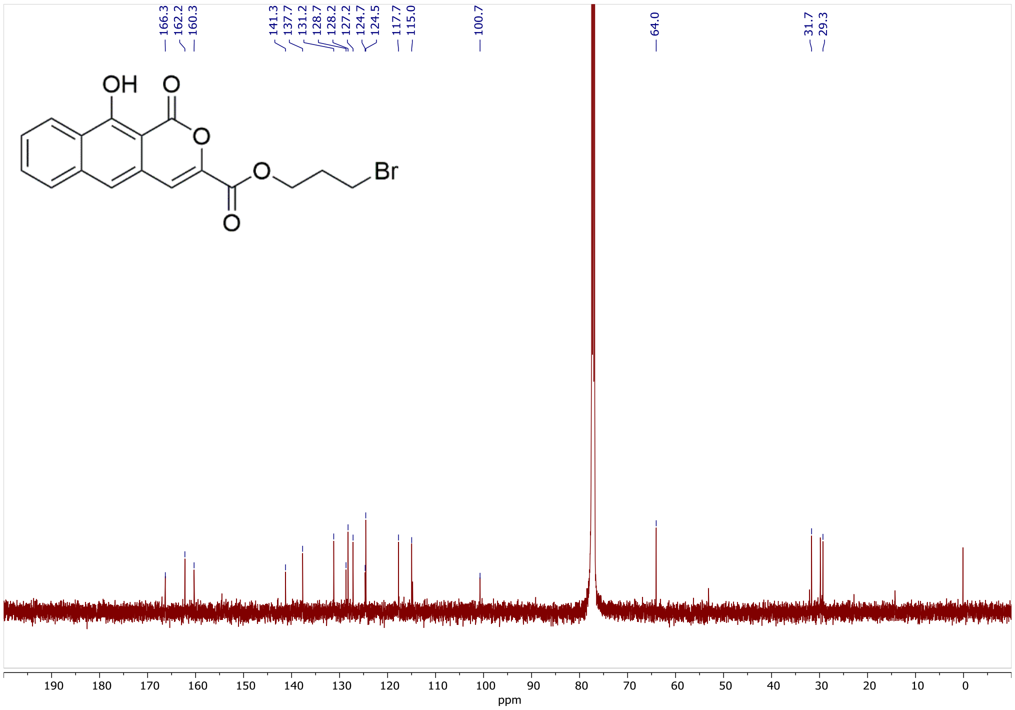

Figure S63. ^{1}H NMR of **12o** plus grease (400 MHz, $CDCl_3$).

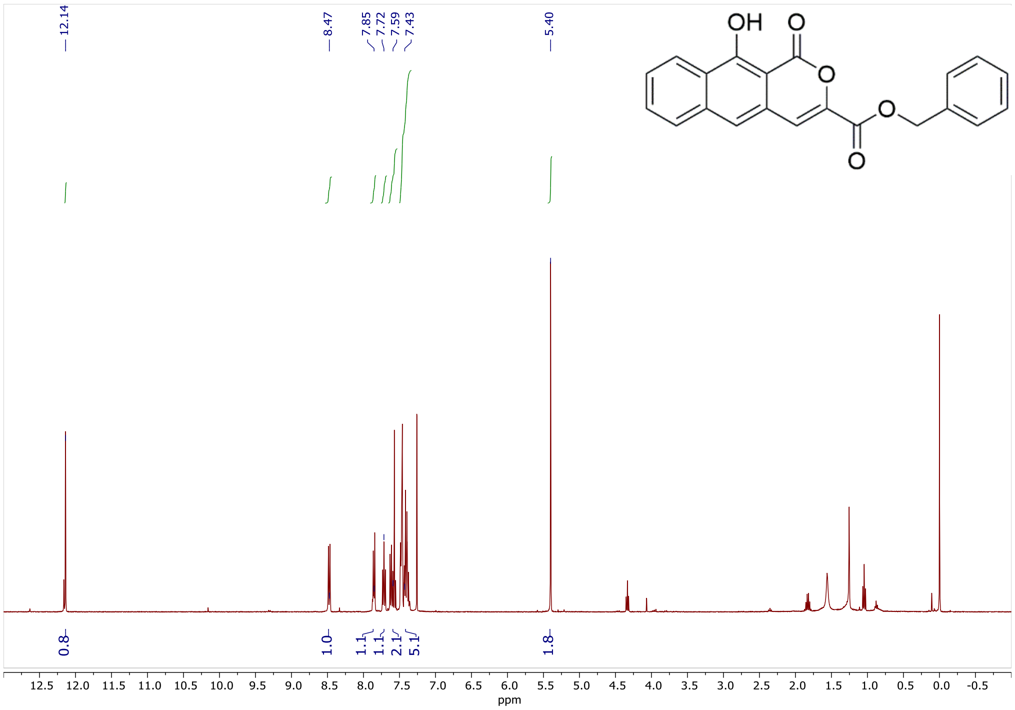

Figure S64. ^{13}C{^{1}H} NMR of **12o** plus grease (101 MHz, $CDCl_3$).

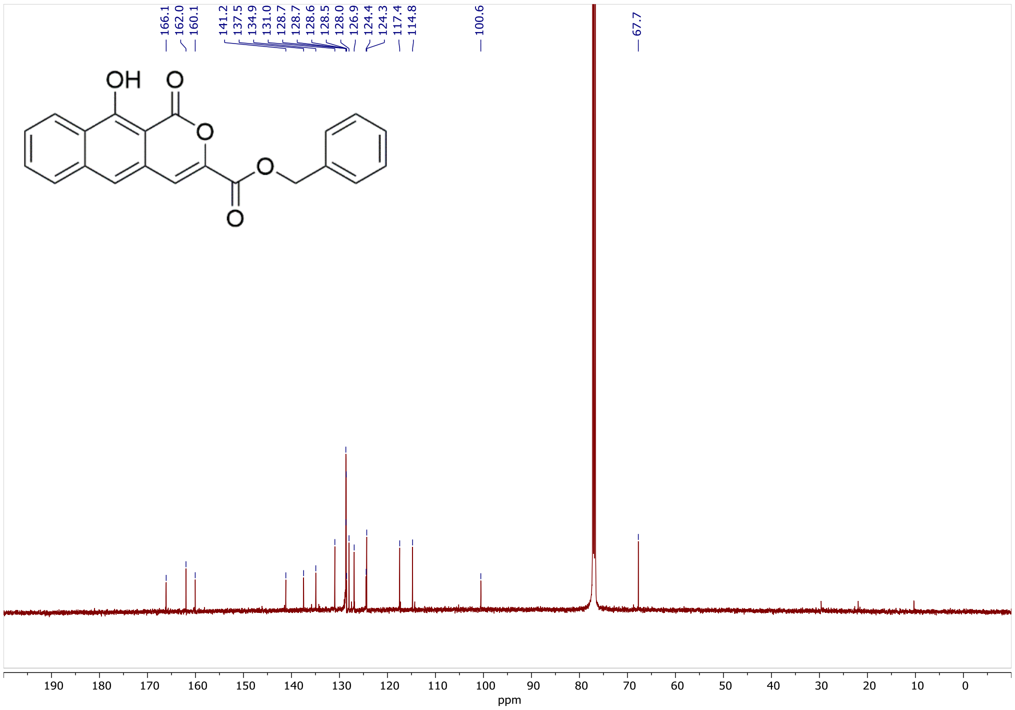

Figure S65 ^{1}H NMR of **12p** plus grease (400 MHz, $CDCl_3$).

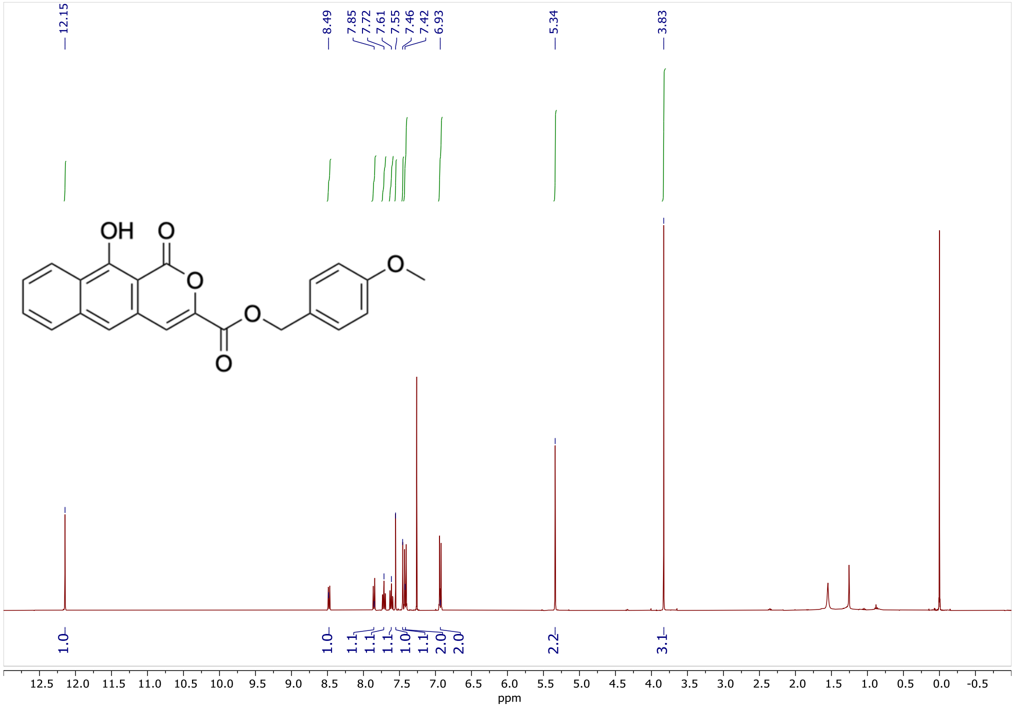

Figure S66. ^{13}C{^{1}H} NMR of **12p** plus grease (126 MHz, $CDCl_3$).

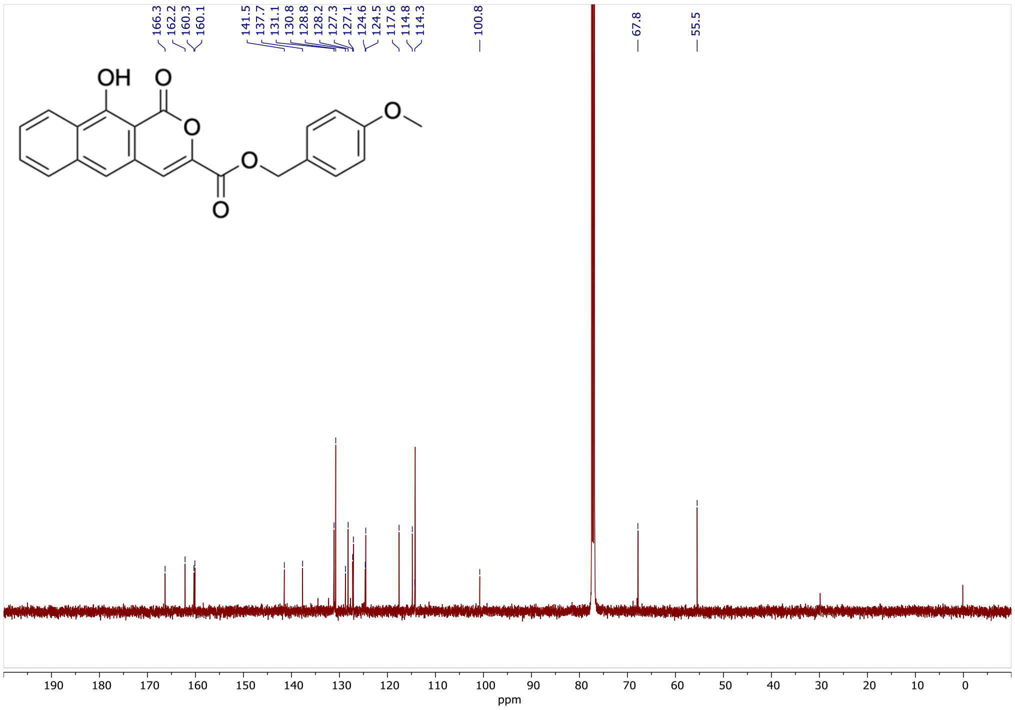

Figure S67. ^{1}H NMR of **12s** (400 MHz, $CDCl_3$).

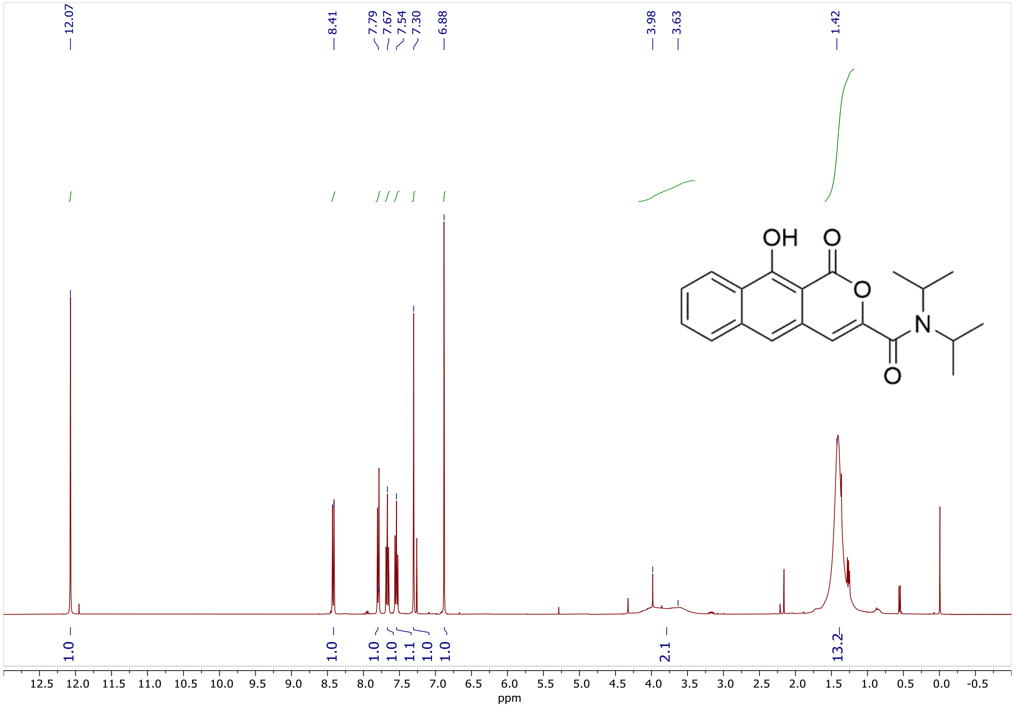

Figure S68. $^{13}C\{^{1}H\}$ NMR of **12s** (101 MHz, $CDCl_3$).

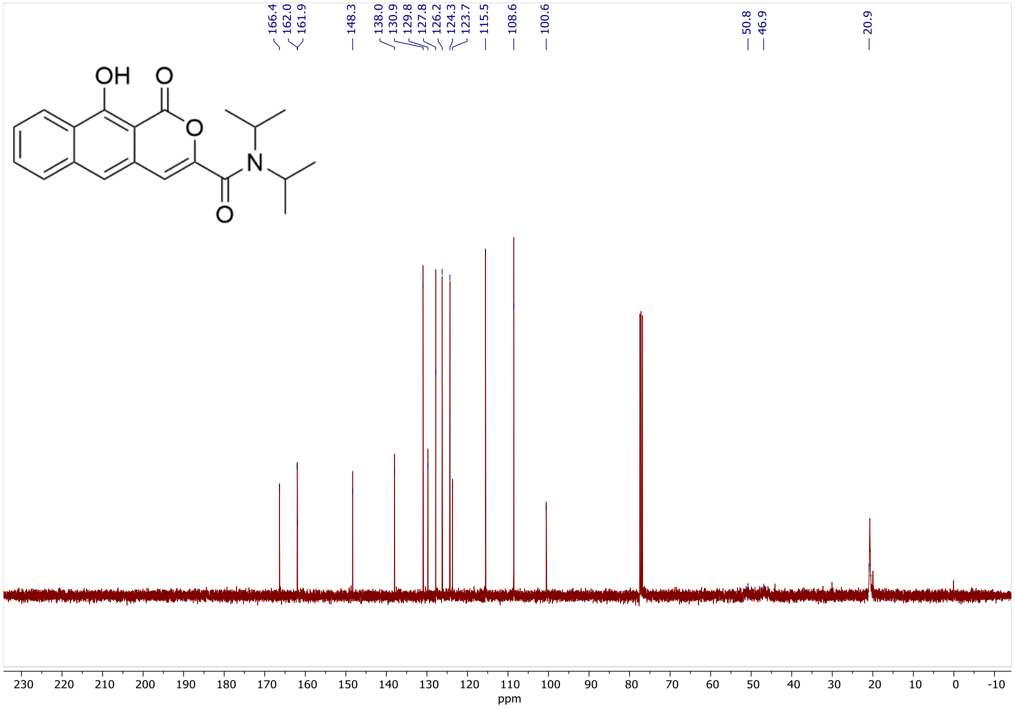

Figure S69. ^{1}H NMR of **12t** (400 MHz, $CDCl_3$).

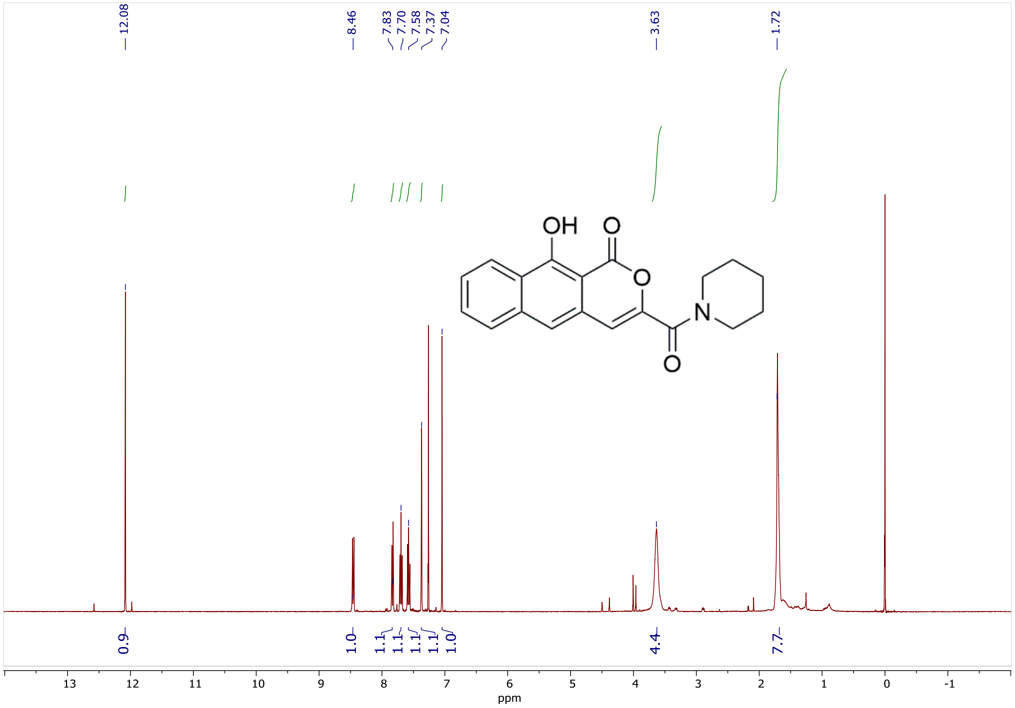

Figure S70. $^{13}C\{^{1}H\}$ NMR of **12t** (100 MHz, $CDCl_3$).

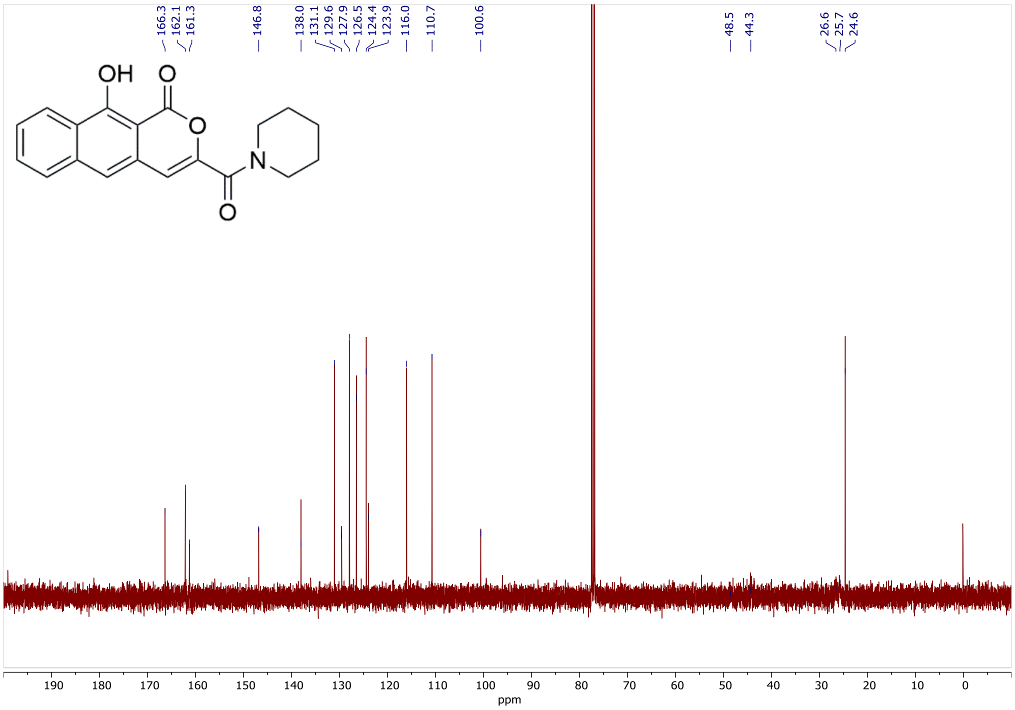

Figure S71. ^{1}H NMR of **12u** (400 MHz, $CDCl_3$).

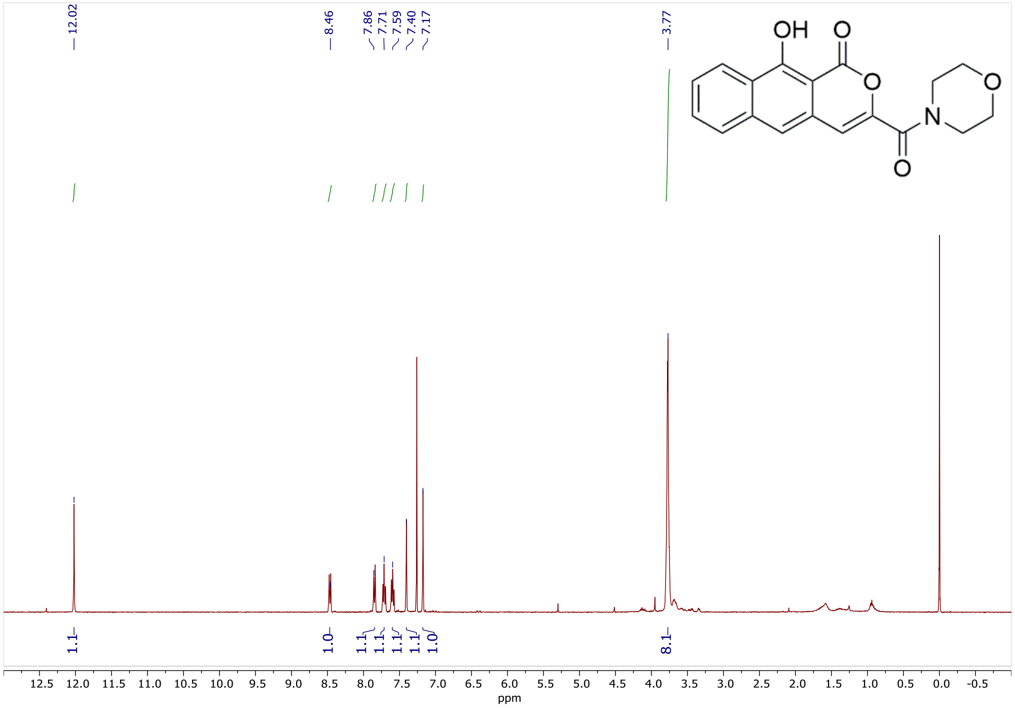

Figure S72. $^{13}C\{^{1}H\}$ NMR of **12u** (101 MHz, $CDCl_3$).

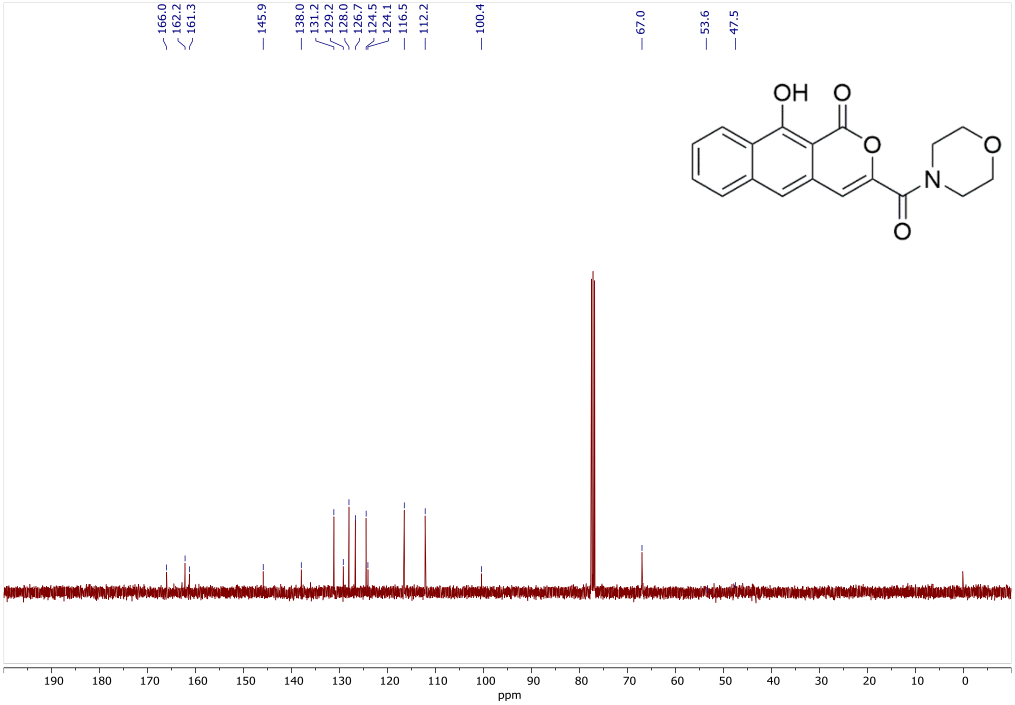

Figure S73. ^{1}H NMR of **11q*** (400 MHz, $CDCl_3$).

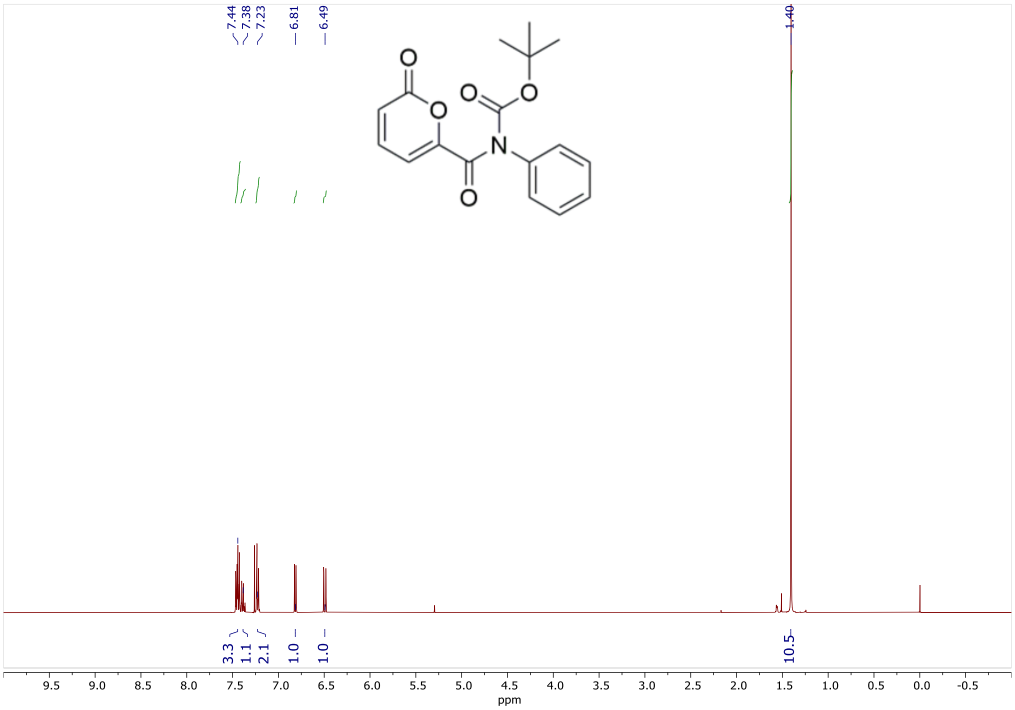

Figure S74. $^{13}C\{^{1}H\}$ NMR of **11q*** (101 MHz, $CDCl_3$).

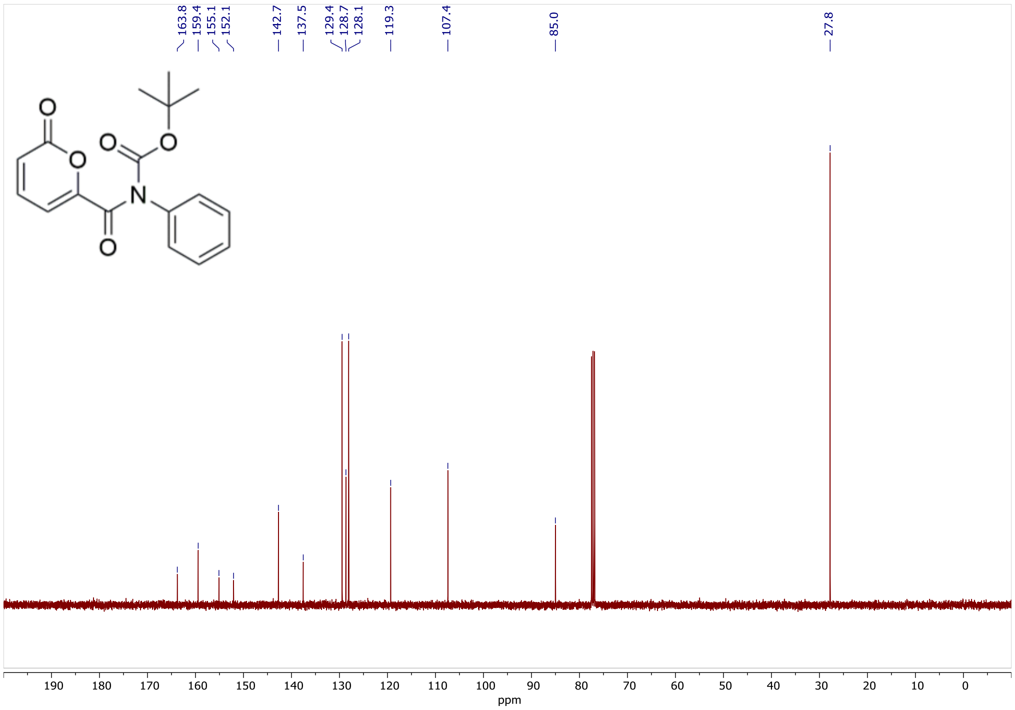

Figure S75. ^{1}H NMR of **11v*** (400 MHz, $CDCl_3$).

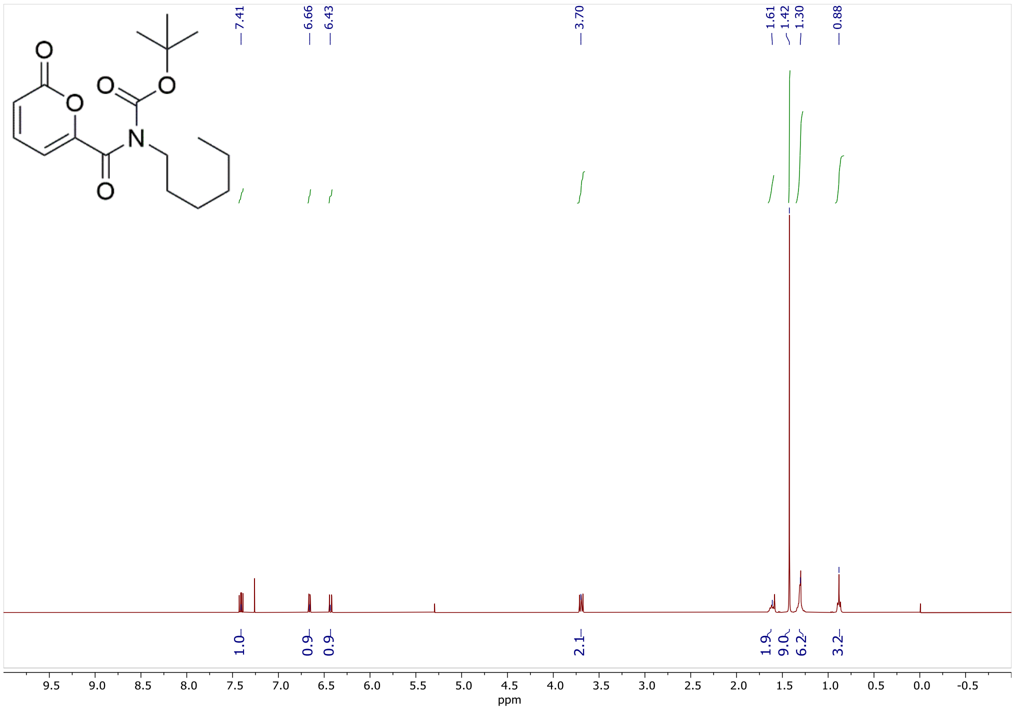

Figure S76. $^{13}C\{^{1}H\}$ NMR of **11v*** (101 MHz, $CDCl_3$).

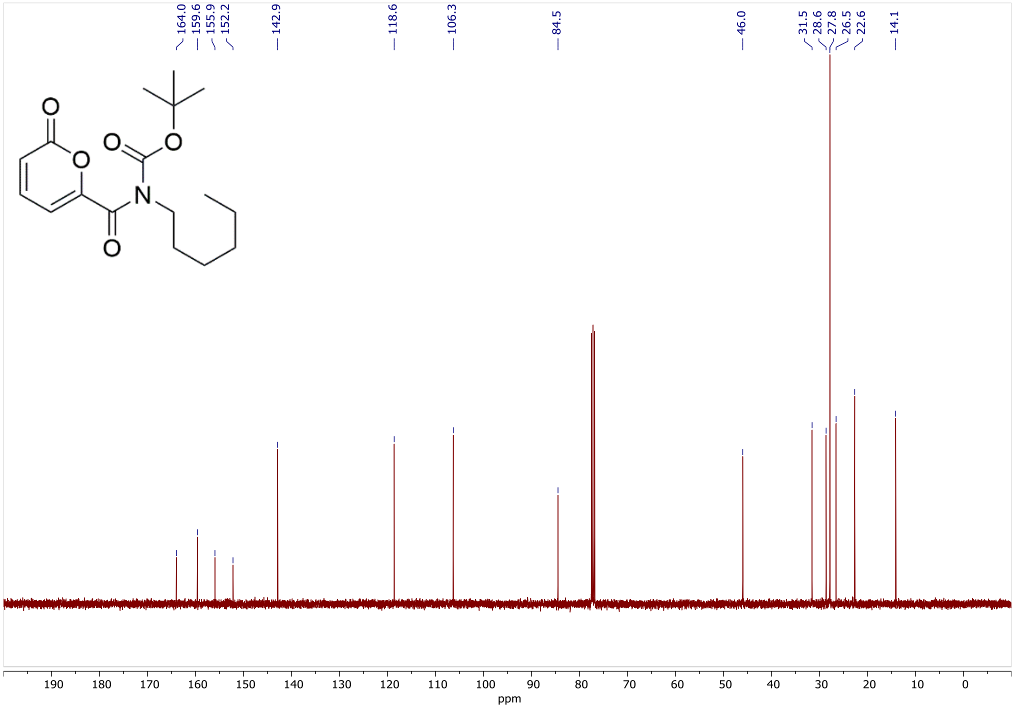

Figure S77. ^{1}H NMR of **12v*** (400 MHz, $CDCl_3$).

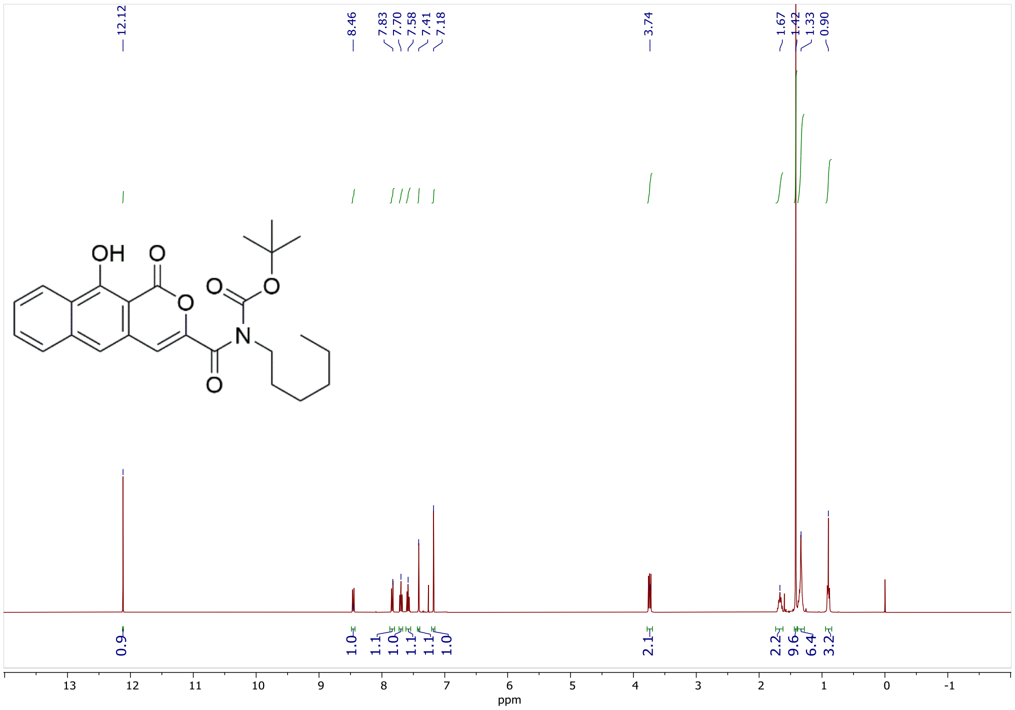

Figure S78. $^{13}C\{^{1}H\}$ NMR of **12v*** (101 MHz, $CDCl_3$).

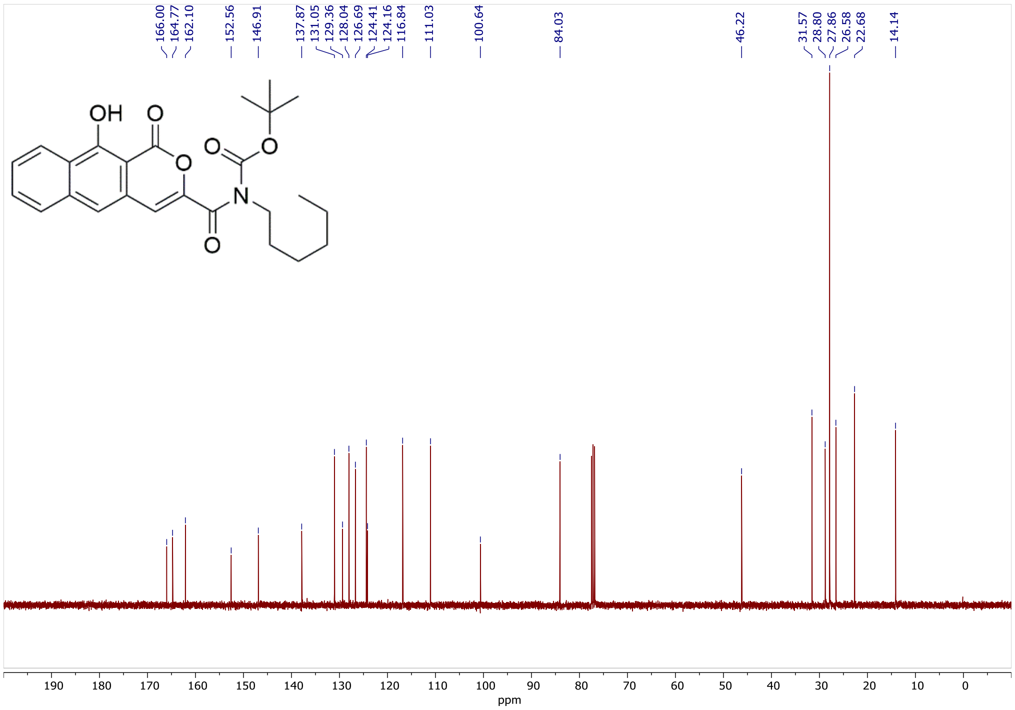

Figure S79. ^{1}H NMR of **13** (400 MHz, $CDCl_3$).

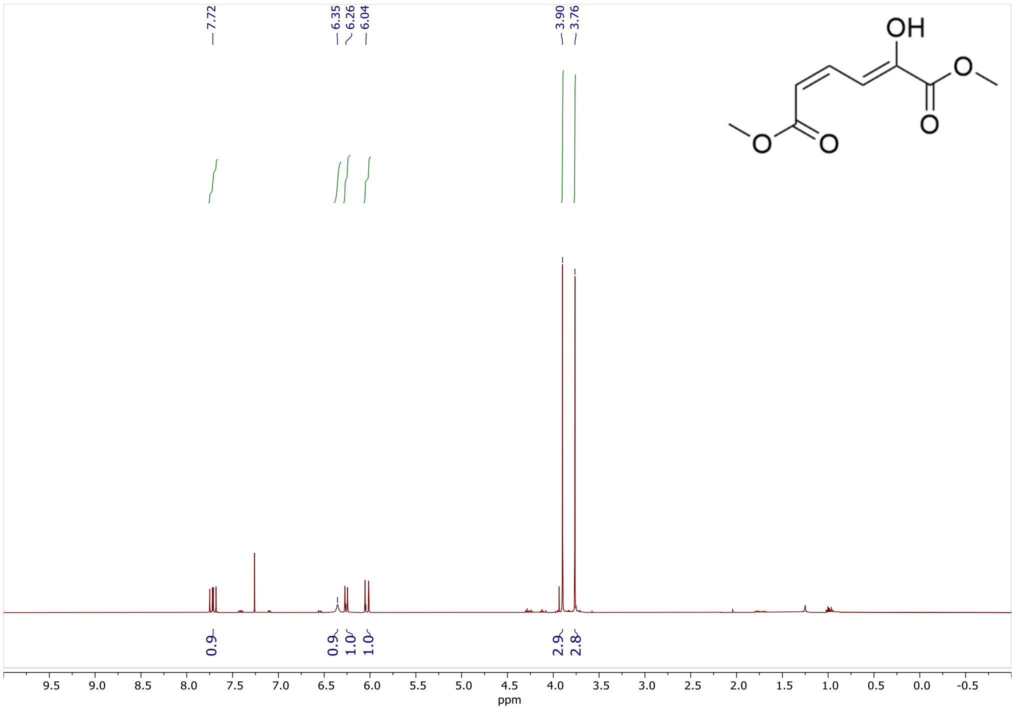

Figure S80. $^{13}C\{^{1}H\}$ NMR of **13** (101 MHz, $CDCl_3$).

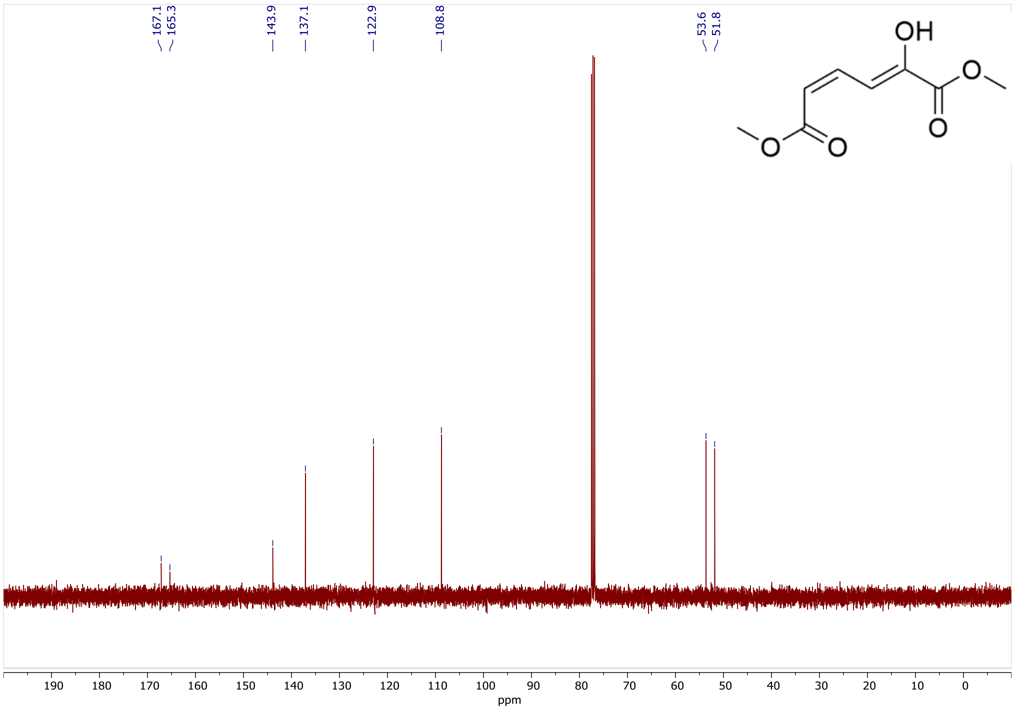

Figure S81. ^{1}H NMR of **15** (400 MHz, CD_3OD).

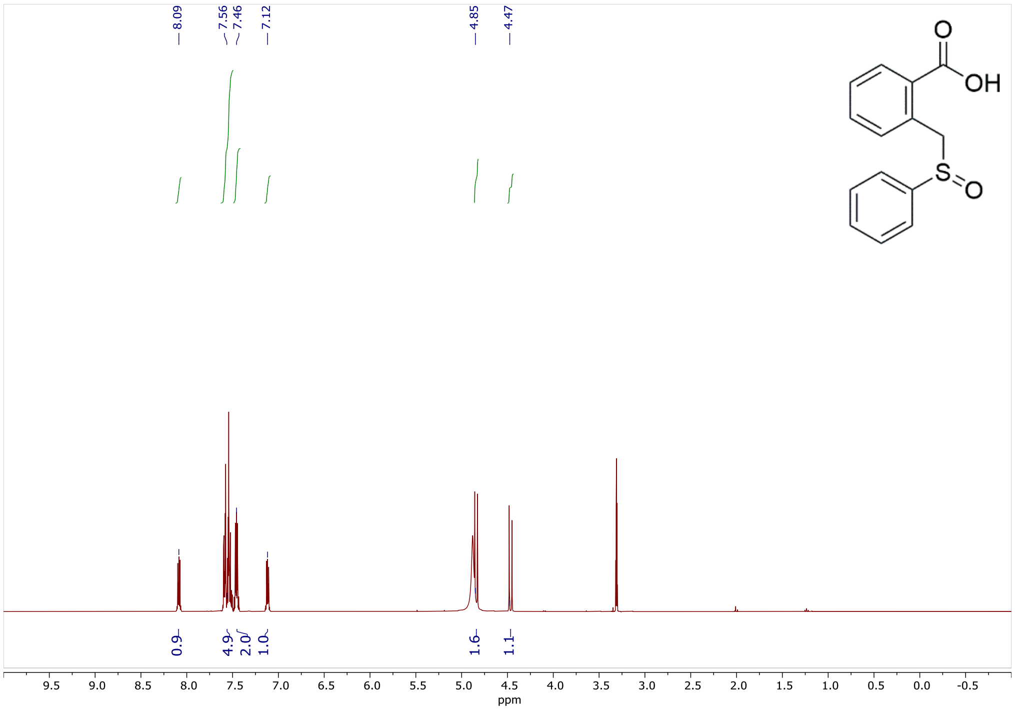

Figure S82. ^{13}C{^{1}H} NMR of **15** (101 MHz, CD_3OD).

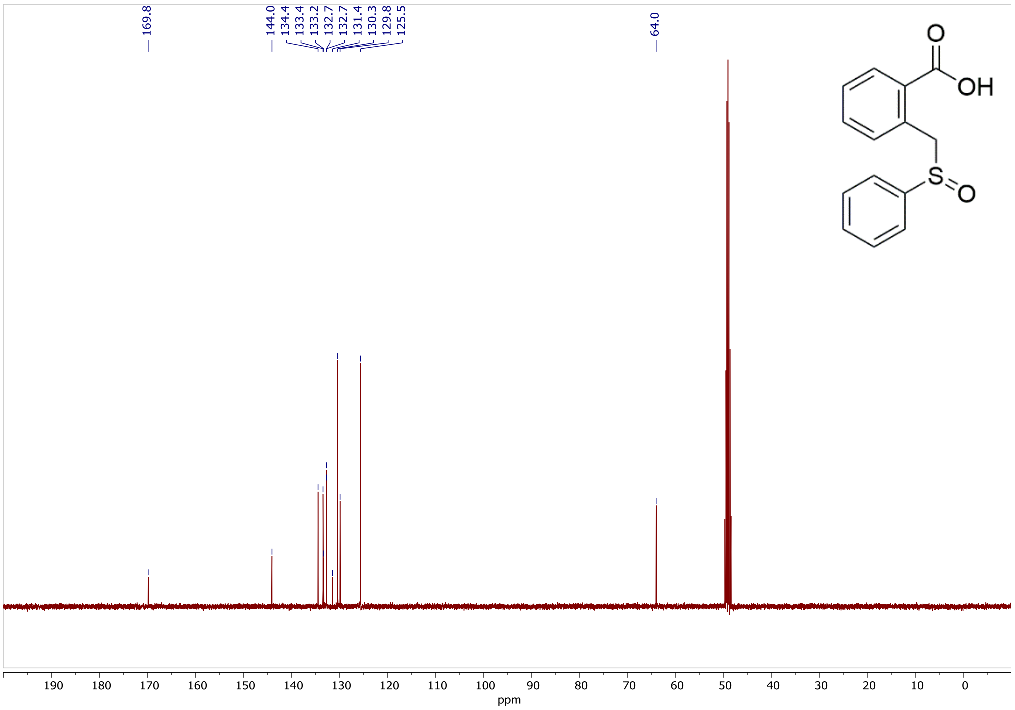

Figure S83. ^{1}H NMR of **16** (400 MHz, $CDCl_3$).

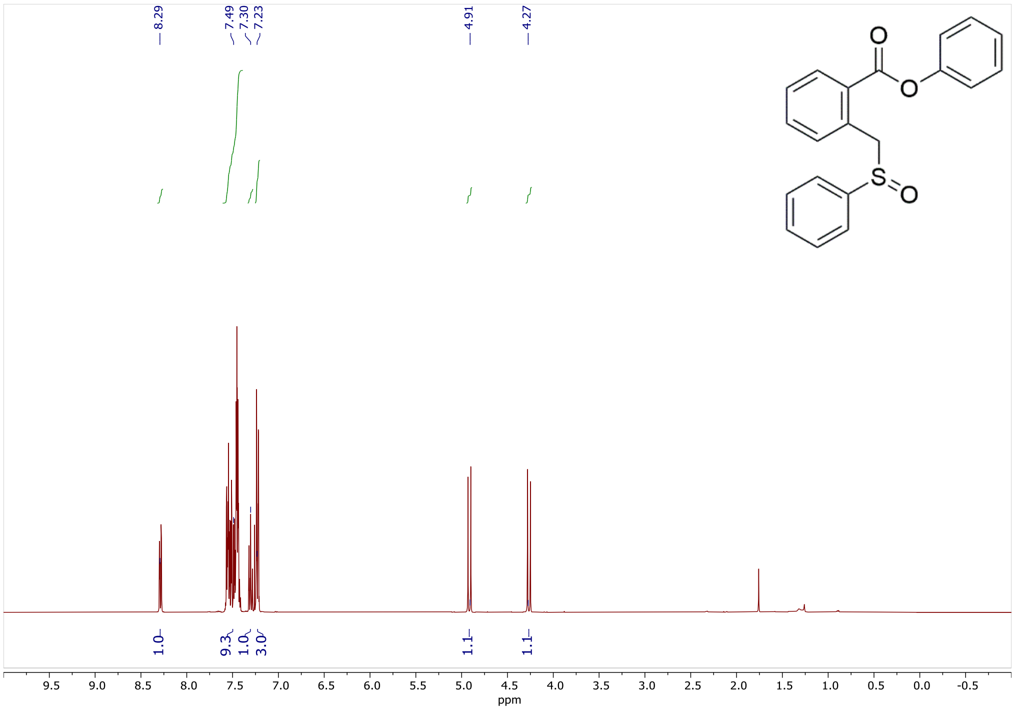

Figure S84. ^{13}C{^{1}H} NMR of **16** (101 MHz, $CDCl_3$).

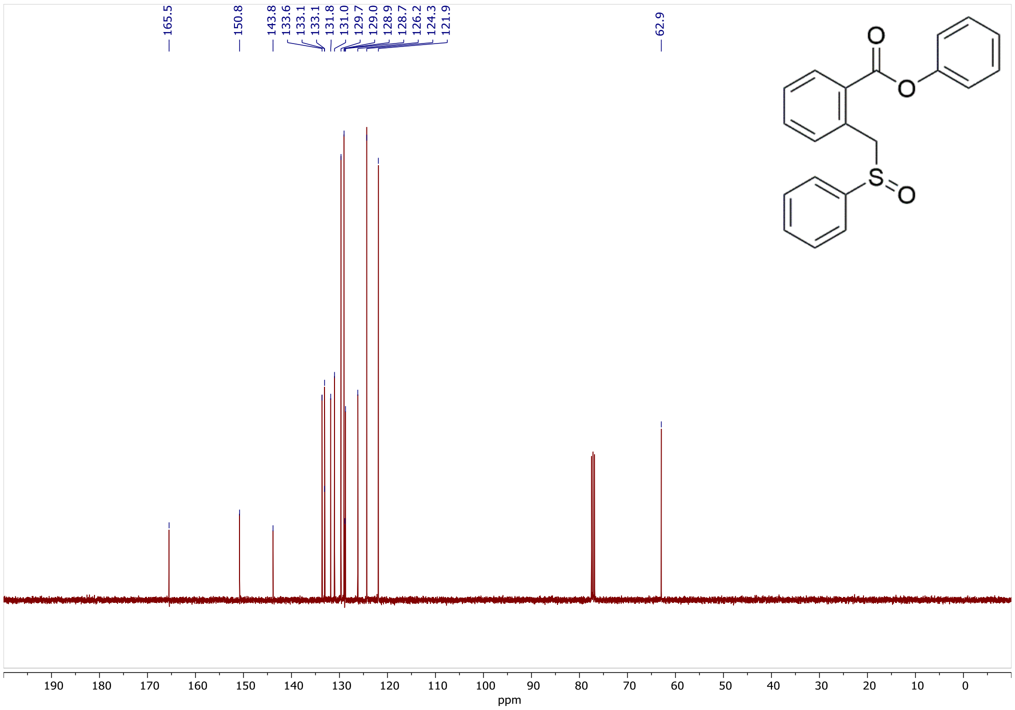

Figure S85. ^{1}H NMR of **18** plus trace acetone (400 MHz, $CDCl_3$).

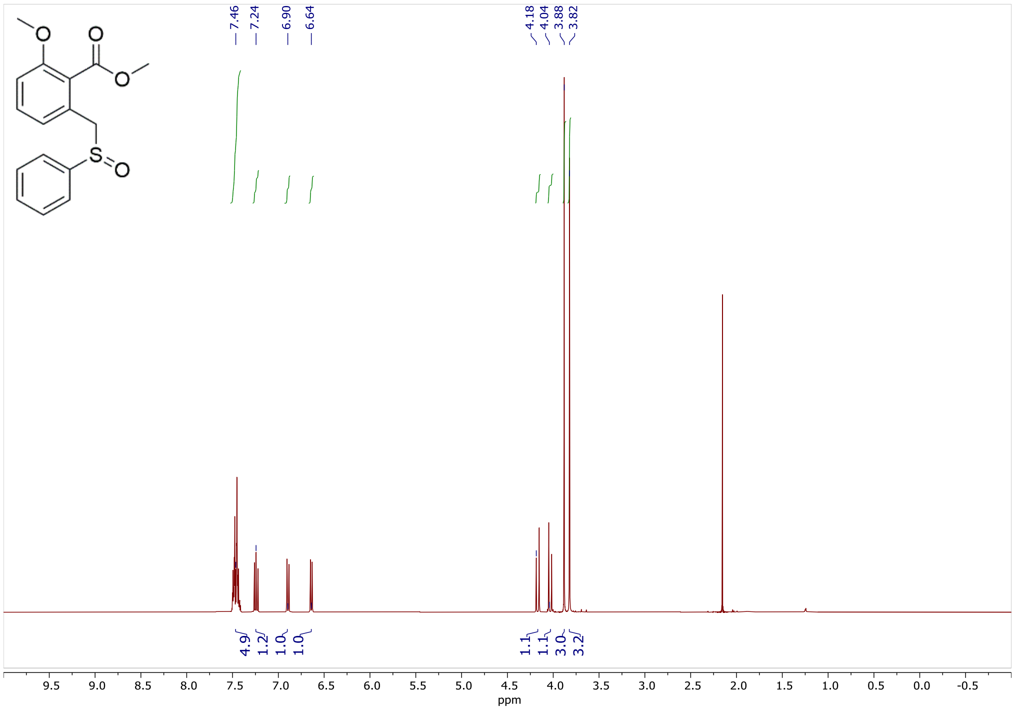

Figure S86. ^{13}C{^{1}H} NMR of **18** plus trace acetone (101 MHz, $CDCl_3$).

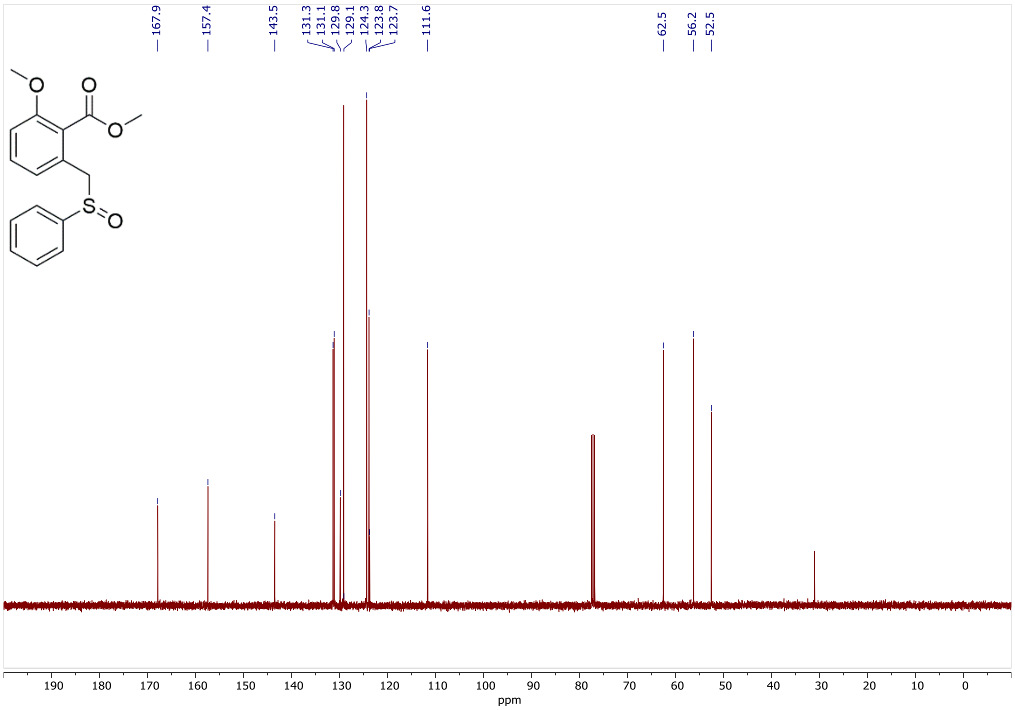

Figure S87. ^{1}H NMR of **19** plus trace water (400 MHz, $CDCl_3$).

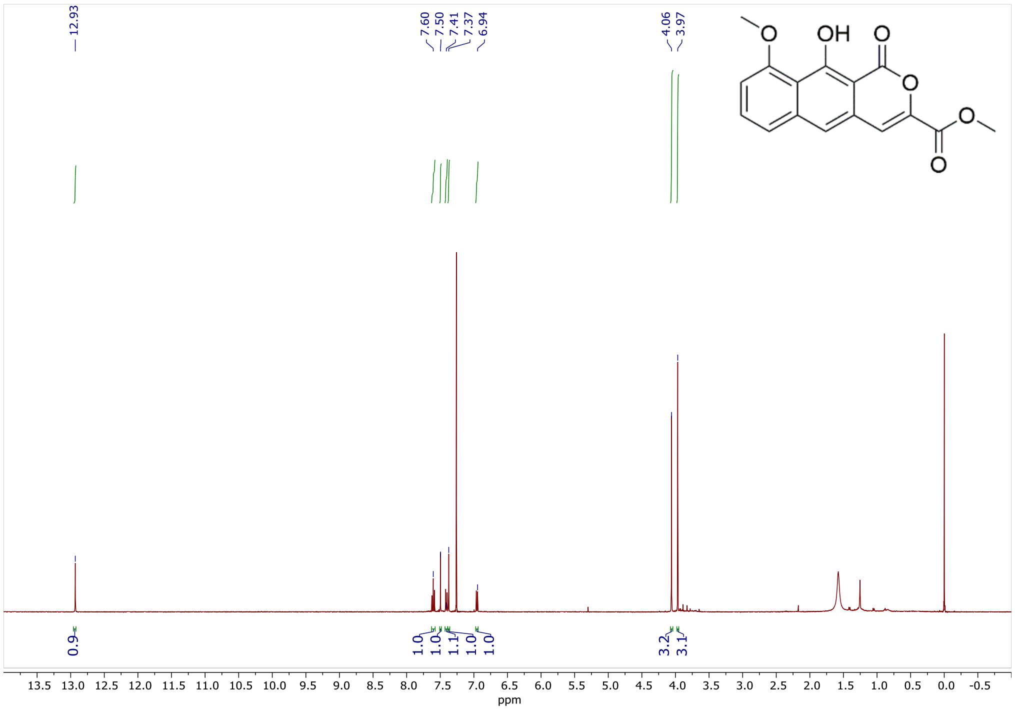

Figure S88. $^{13}C\{^{1}H\}$ NMR of **19** (151 MHz, $CDCl_3$).

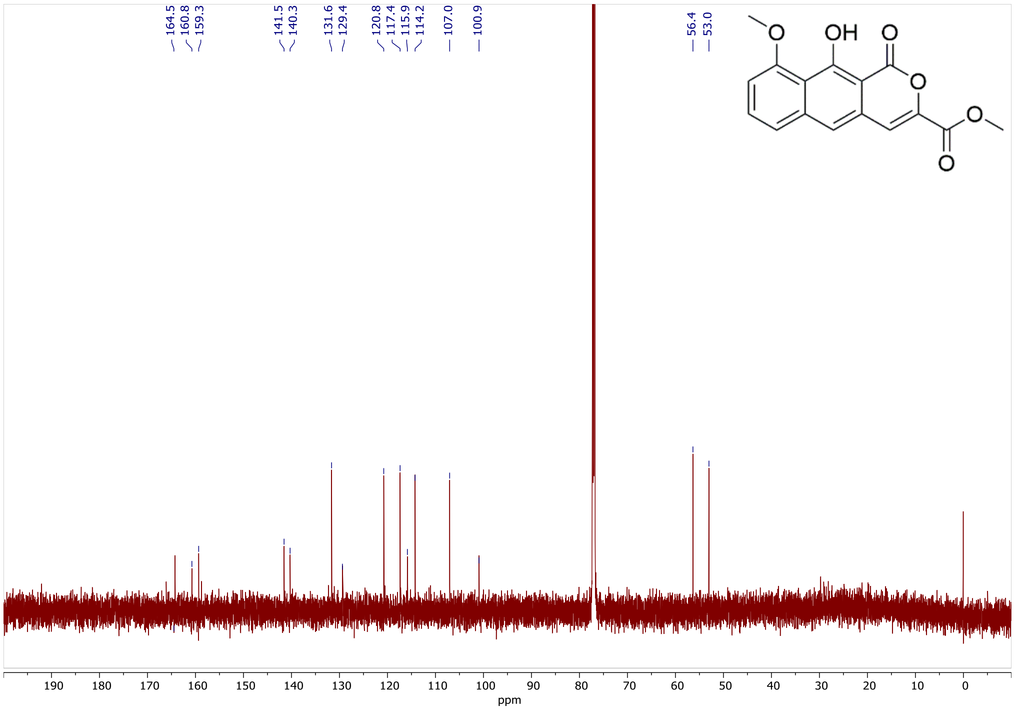

Figure S89. ^{1}H NMR of **22** (400 MHz, CDCl$_3$).

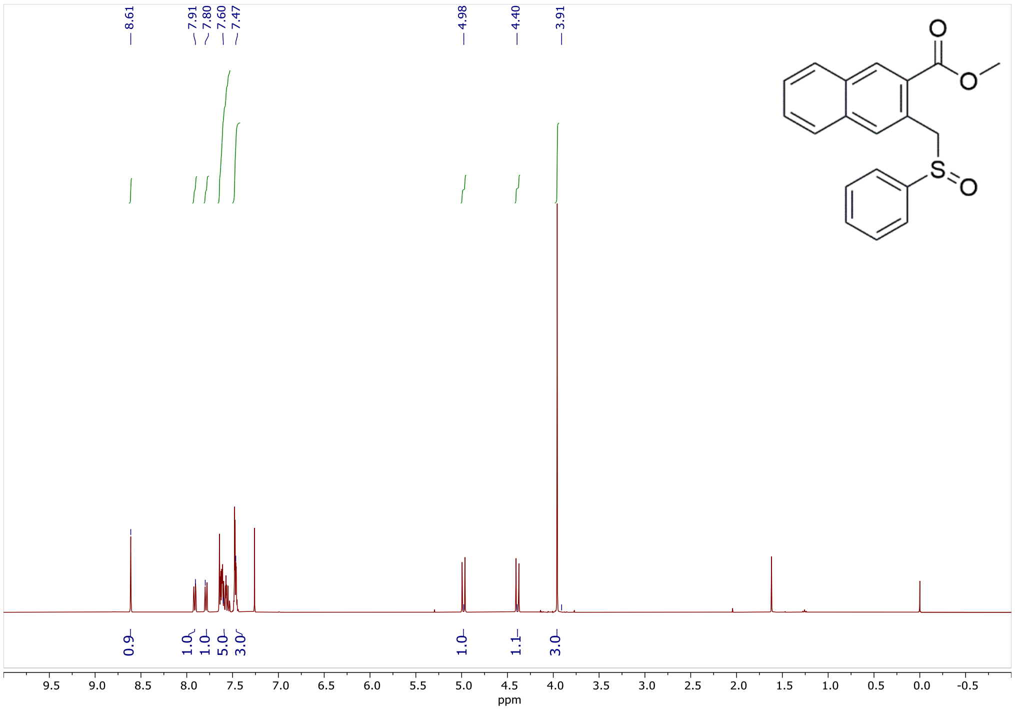

Figure S90. ^{13}C{^{1}H} NMR of **22** (100 MHz, CDCl$_3$).

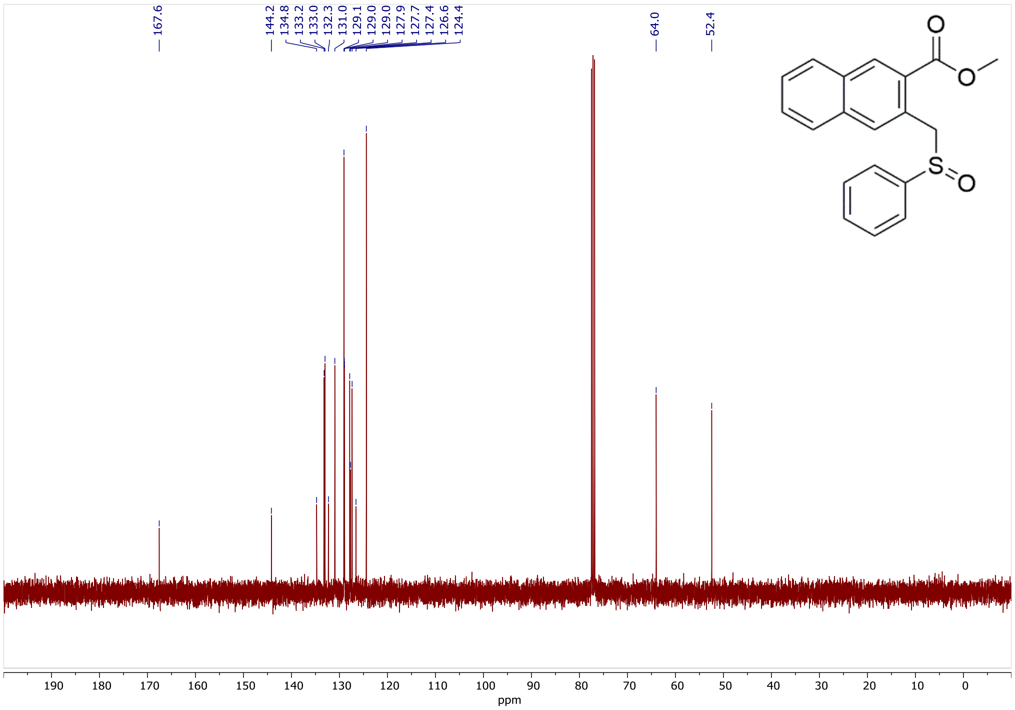

Figure S91. ^{1}H NMR of **23** (400 MHz, $CDCl_3$).

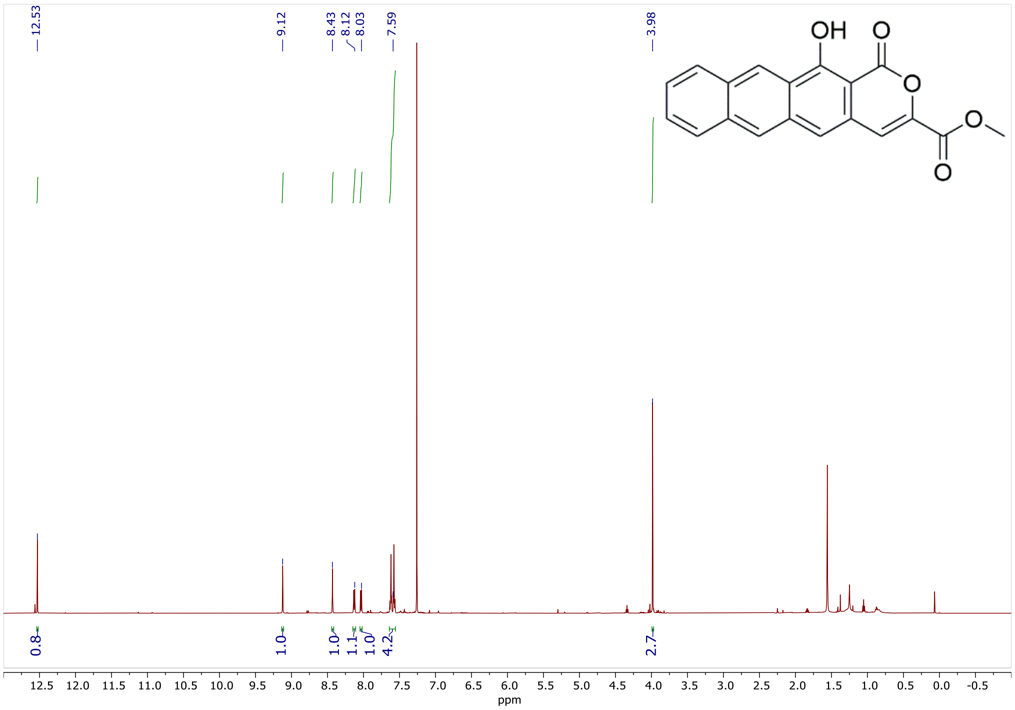

Figure S92. $^{13}C\{^{1}H\}$ NMR of **23** (151 MHz, $CDCl_3$).

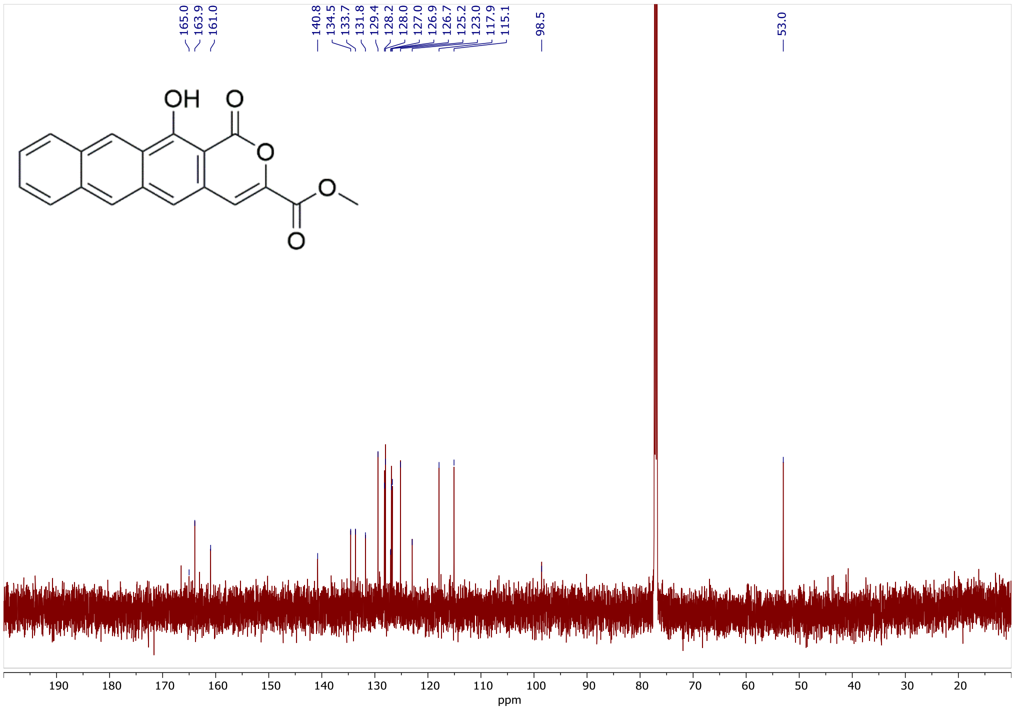

Supplement: Supplementary file 1 — jo4c01044_si_001.pdf [file jo4c01044_si_001.pdf]
